# Synthesis and Evaluation of Chloramphenicol Homodimers: Molecular Target, Antimicrobial Activity, and Toxicity against Human Cells

**DOI:** 10.1371/journal.pone.0134526

**Published:** 2015-08-12

**Authors:** Ourania N. Kostopoulou, George E. Magoulas, Georgios E. Papadopoulos, Athanasia Mouzaki, George P. Dinos, Dionissios Papaioannou, Dimitrios L. Kalpaxis

**Affiliations:** 1 Department of Biochemistry, School of Medicine, University of Patras, Patras, Greece; 2 Laboratory of Synthetic Organic Chemistry, Department of Chemistry, University of Patras, Patras, Greece; 3 Department of Biochemistry and Biotechnology, University of Thessaly, Ploutonos, Larissa, Greece; 4 Division of Hematology, Department of Internal Medicine, School of Medicine, University of Patras, Patras, Greece; University of Cambridge, UNITED KINGDOM

## Abstract

As fight against antibiotic resistance must be strengthened, improving old drugs that have fallen in reduced clinical use because of toxic side effects and/or frequently reported resistance, like chloramphenicol (CAM), is of special interest. Chloramphenicol (CAM), a prototypical wide-spectrum antibiotic has been shown to obstruct protein synthesis via binding to the bacterial ribosome. In this study we sought to identify features intensifying the bacteriostatic action of CAM. Accordingly, we synthesized a series of CAM-dimers with various linker lengths and functionalities and compared their efficiency in inhibiting peptide-bond formation in an *Escherichia coli* cell-free system. Several CAM-dimers exhibited higher activity, when compared to CAM. The most potent of them, compound **5**, containing two CAM bases conjugated via a dicarboxyl aromatic linker of six successive carbon-bonds, was found to simultaneously bind both the ribosomal catalytic center and the exit-tunnel, thus revealing a second, kinetically cryptic binding site for CAM. Compared to CAM, compound **5** exhibited comparable antibacterial activity against MRSA or wild-type strains of *Staphylococcus aureus*, *Enterococcus faecium* and *E*. *coli*, but intriguingly superior activity against some CAM-resistant *E*. *coli* and *Pseudomonas aeruginosa* strains. Furthermore, it was almost twice as active in inhibiting the growth of T-leukemic cells, without affecting the viability of normal human lymphocytes. The observed effects were rationalized by footprinting tests, crosslinking analysis, and MD-simulations.

## Introduction

The rapid and progressive prevalence of antibiotic resistance urges for intensified research in the development of compounds with potent antimicrobial activities. Along these lines, the improvement of the structural and physicochemical properties of existing antibiotics constitutes an extremely effective approach in the reduction of both toxic side effects and reported resistance.

**Fig 1:**
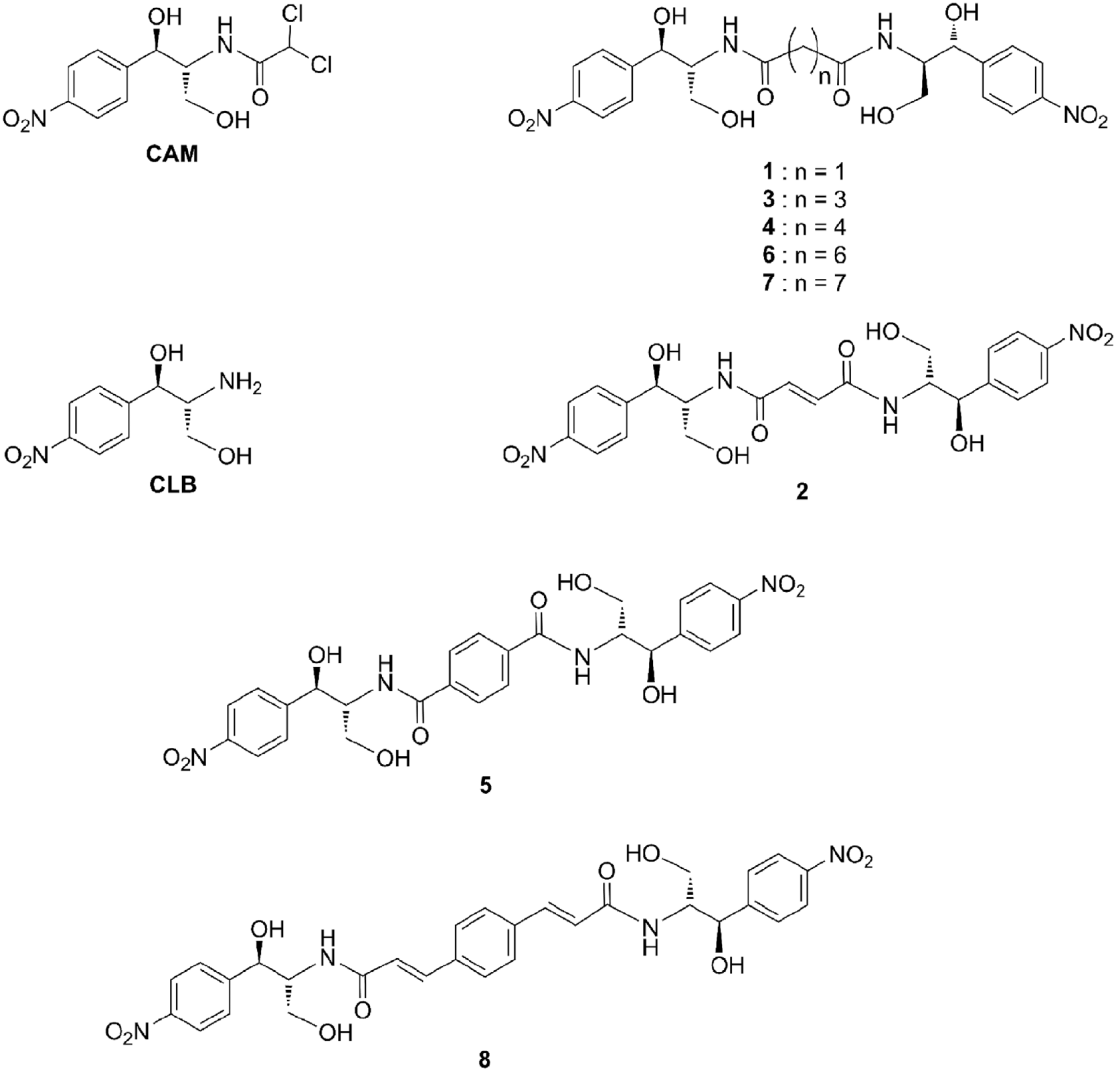
Structures of CAM, CLB, and the synthesized CAM dimers. Abbreviations: CAM, chloramphenicol; CLB, chloramphenicol base.

Peptidyl transferase (PTase) activity, i.e. the activity of ribosomes to catalyze the peptide-bond formation, resides in the large ribosomal subunit, and in prokaryotes is one of the most thoroughly validated targets for antibiotics, including chloramphenicol (CAM) [1,2]. CAM is a broad—spectrum bacteriostatic agent, consisting of a *p*-nitrophenyl ring attached to a dichloroacetyl tail via a 2-amino-1,3-propanediol moiety (Fig 1). As detected by crystallographic analysis in bacteria [3–5], it binds within the catalytic crevice of the PTase center (CAM1), blocking essential ribosomal functions, such as peptide-bond formation [6], termination of translation [7], and translational accuracy [8]. Contrary to bacteria, the chloramphenicol binding site in the archaeal *Haloarcula marismortui* 50S subunit is located at the entrance to the peptide exit-tunnel (CAM2), which is overlapping with the binding site of macrolide antibiotics [9].

Earlier equilibrium dialysis studies, reviewed by Pongs [10], have reported two binding sites for CAM on *Escherichia coli* ribosomes; a high affinity site (*K*_*D*_ = 2 *μM*) verified by earlier and recent kinetic studies [6,11], and a low affinity site (*K*_*D*_ = 200 *μM*). Cross-linking of CAM to ribosomes of the bacterium *E*. *coli* and the archaeal *Halobacterium halobium* identified interactions of the drug with nucleotides clustered around the entrance to the peptide exit-tunnel [12]. However in this study, high concentrations of CAM (1.2 mM) were required in order to produce crosslinking with 23S rRNA. Consequently, the functional significance of the second binding site of CAM (CAM2) remains elusive, whereas it has been firmly demonstrated that binding of CAM adjacent to the A-site of the PTase center inhibits the accommodation of the 3΄-aminoacyl end of tRNA within the catalytic crevice [11]. Nevertheless, the CAM2 site, if it really exists, could be exploited for the binding of CAM dimers bearing a correctly adjusted linker. Specifically, an optimally designed CAM dimer could promote binding of the first pharmocophore to the high affinity site and of the second one to the low affinity site. This could be easily achieved, since the unbound, but tethered pharmacophore acquires a very high local concentration from seeking out its cognate target within a sphere having a radius that corresponds to the length of the linker [13].

Resistance to CAM has been frequently reported, and attributed to numerous mechanisms, such as target mutations or alterations [14–17], drug modifications [18], decreased membrane permeability [19], and over-expression of efflux pumps [20]. However, the major concerns that hamper its clinical use relate to the adverse effects of causing hematologic disorders, like reversible bone marrow depression, aplastic anemia, and leukemia [21]. To define its essential functionalities and to improve its pharmacological properties, CAM has been modified in many ways [22]. Recently, we synthesized a series of CAM-polyamine conjugates and demonstrated that addition of the polyamine moiety provided enhanced binding properties and increased membrane permeability to the constructs [11]. To extend these findings, we have synthesized and evaluated the biological properties of a series of CAM homodimers. The potential benefits of this strategy, which has been proved useful in several other applications [13,23–26], include: (i) improvement of the biological activity of CAM, since the presence of dimers can occupy multiple functional sites of the target, (ii) enhancement of the binding affinity, because CAM dimers are capable of simultaneously binding two separated RNA sites, and (iii) better potency against resistant bacterial strains. Nevertheless, a number of drawbacks need to be considered when developing such antibacterials, like cell permeability problems and unexpected binding to additional targets, as has been reported in previous studies [11,23–26]. Fig 1 provides a schematic representation of the constructs used in the present study. These include, two CAM free base units (CLB) attached on dicarboxylic acids, through amide bonds. The chain of dicarboxylic acids was either aliphatic of variable length (compounds **1**, **3**, **4**, **6** and **7**), olefinic (compound **2**), or aromatic (compounds **5** and **8**). With these particular dimers, we wanted to examine the effect of (i) the length of the aliphatic chain (linker) connecting the two CLB units (e.g. **1** and **7**) and (ii) the nature and the flexibility of the linker (e.g. **4** and **5**) on the inhibitory activity of the homodimers on peptide bond formation in a cell-free system and the antibacterial activity against wild type or resistant bacterial strains. Compound **5**, ranking among the most potent members in the group of CAM dimers *in vitro*, was further studied for its ability to reduce the viability of human peripheral blood cells and to restrain the proliferation of human leukemic cells. The promising findings for compound **5** show that its structure can be fruitfully used for designing more potent, but less toxic antibacterials.

## Results and Discussion

### Synthesis of ribosome-targeting CAM dimers

**Fig 2:**
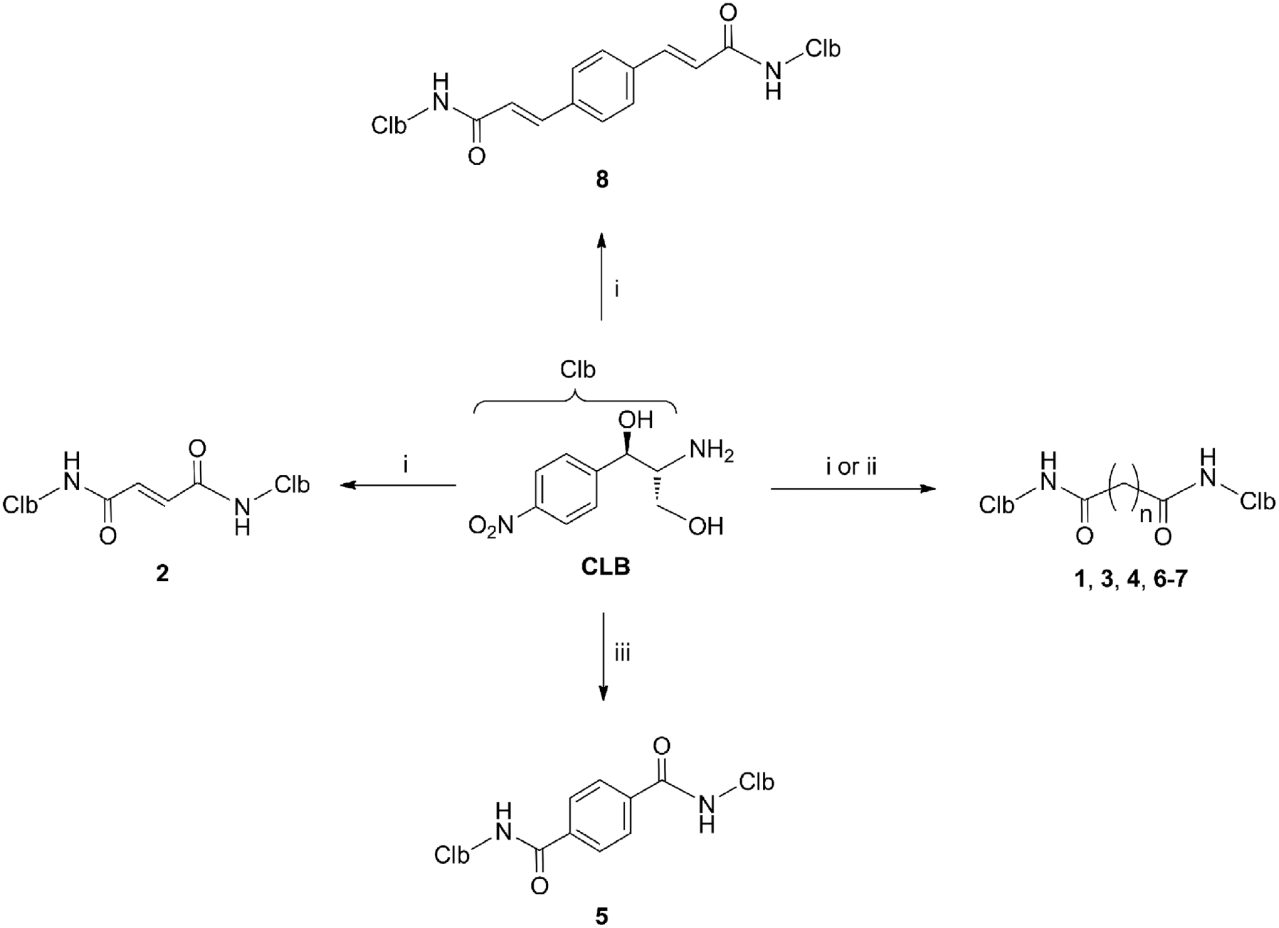
Synthesis of compounds studied in the present work. Reagents and conditions: (i) malonic acid (for compound 1), fumaric acid (for compound 2), adipic acid (for compound 4), suberic acid (for compound 6), azelaic acid (for compound 7), 1,4-phenylenediacrylic acid (for compound 8), HBTU, ^i^Pr_2_NEt, DMF, 0°C then RT, 1–3 h; yield: 75% (1), 55% (2), 85% (4), 67% (6), 89% (7), and 83% (8); (ii) (a) glutaric anhydride, DMF, RT, 2h; (b) HBTU, ^i^Pr_2_NEt, DMF, 0°C then RT, 1 h; yield: 80% (3); (iii) terephthaloyl chloride, Et_3_N, DMF, 0°C then RT, 1h; yield: 80% (5). See also S1 Supplemental Procedures for details.

The synthesis of CAM dimers **1**–**8** is depicted in Fig 2 (see also S1 Supplemental Procedures). Compounds **1**, **2**, **4** and **6**–**8** were readily assembled by the condensation of the commercially available CLB with the corresponding carboxylic acids malonic, fumaric, adipic, suberic, azelaic and 1,4-phenylenediacrylic, in the presence of the coupling agent *O*-(benzotriazol-1-yl)-*N*,*N*,*N*’,*N*’-tetramethyluronium hexafluorophosphate (HBTU) and ethyldiisopropylamine, in 55–89% yields. Compound **5** was obtained in 80% yield by condensing CLB and terephthaloyl chloride in the presence of triethylamine. Finally, compound **3** was assembled in 80% yield by first acylating CLB with glutaric anhydride and then coupling the resulting acid with additional CLB in the presence of HBTU and ethyldiisopropylamine.

### Inhibition of peptide-bond formation by CAM dimers

The inhibitory effect of CAM dimers on peptide-bond formation was studied using the puromycin reaction, a model reaction between puromycin and a post-translocation ribosomal complex (complex C) derived from *E*. *coli* [6]. Since puromycin, a pseudo-substrate of PTase which binds to the A-site of the catalytic center, was present in excess, the reaction obeyed first-order kinetics. The first-order rate constant, *k*_obs_, at each concentration of puromycin was determined by fitting the *x* values into Eq 1,

$$ln\frac{100}{100-x}=k_{obs}t$$ (1)

where *x* is the product AcPhe-puromycin, expressed as the percentage of complex C added in the reaction mixture, and *t* is the time of the reaction.

**Fig 3:**
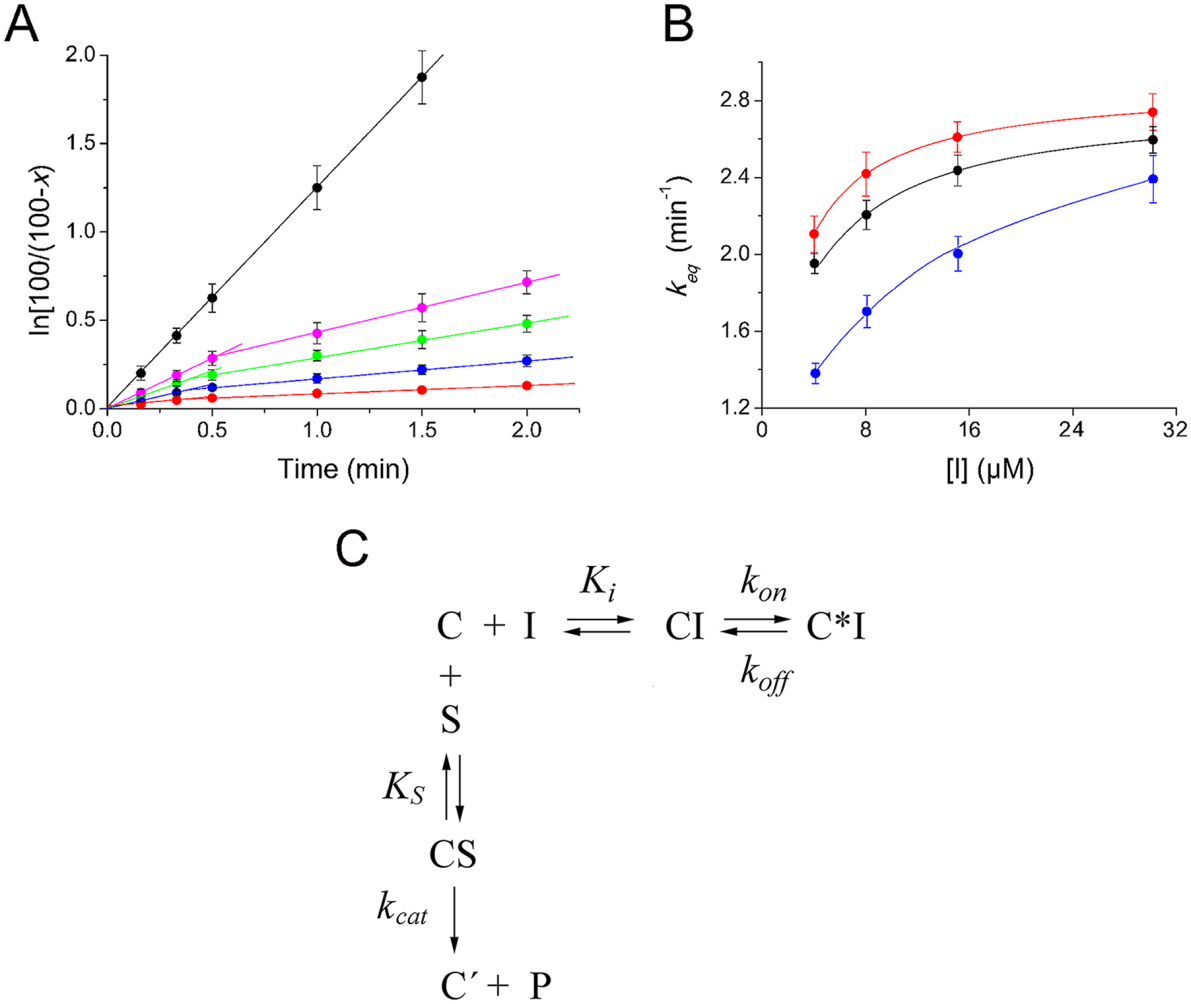
AcPhe-puromycin synthesis in the presence or absence of compound 5. (A) First-order time plots; complex C reacted at 25°C in buffer A, with (black) 400 μM puromycin or with a mixture containing 400 μM puromycin and compound 5 at concentrations of 4 μM (magenta), 8 μM (green), 15 μM (blue), and 30 μM (red). (B) Variation of the apparent equilibration rate constant, *k*_eq_, as a function of compound 5 concentration (I). The reaction was carried out in buffer A, in the presence of puromycin at concentrations of 200 μM (red), 400 μM (black), or 2 mM (blue). The *k*_eq_ values were determined by non linear regression fitting of the kinetic data to Eq 2 [11]: (C) Kinetic model for the inhibition of the puromycin reaction by CAM dimers. Symbols: C, poly(U)-programmed ribosomes from *E*. *coli*, bearing AcPhe-tRNA^Phe^ at the P-site of the catalytic center and tRNA^Phe^ at the E-site; I, CAM dimer; S, puromycin; C’, ribosomal complex not recycling; P, AcPhe-puromycin. See also S1 Fig.

A representative time plot obtained at 400 μM puromycin, in the absence of inhibitor, is illustrated in Fig 3A (upper line) and, as expected, is characterized by linearity. However, when the reaction proceeded in the presence of a CAM dimer, e.g. compound **5**, the time plots were characterized by two unique features that distinguish them from a typical kinetic behavior. First, biphasic logarithmic time plots were obtained, with the second phase exhibiting stronger inhibition characteristics than the first one. Second, the slopes of both progress curves varied as a function of the inhibitor concentration (Fig 3A, four lower curves). When analyzed by double reciprocal plotting (1/*k*_obs_ versus 1/[S]; [S] is the concentration of puromycin), both phases exhibited characteristics of simple competitive inhibition (S1 Fig, panels A and B). These kinetic results are consistent with compound **5** operating through an induced fit mechanism (Fig 3C), in which the inhibitor first binds rapidly to complex C to form the encounter complex CI, which then undergoes a slow conformational change to produce a final, tighter complex C*I. Corroborative evidence for the consistency of this model is provided by the hyperbolic shape of the equilibration plots (*k*_eq_ versus [I]), in which *k*_eq_ represents the apparent rate constant for the attainment of equilibrium among C, CI, and C*I (Fig 3B). If one-step mechanism of inhibition was applicable (*C* + *I* ⇄ C*I), *k*_eq_ should be a linear function of [I] [27]. Yet the apparent association rate constant, (*k*_on_ + *k*_off_)/*K*_i_, was found to be 3×10^4^M^1^s^-1^ that is lower than the upper limit 10^6^M^-1^s^-1^ set for the characterization of a compound as a slow-binding inhibitor [27]. Because the isomerization constant *k*_on_/*k*_off_ was calculated to be 3.6, the inhibition process was finally associated with high overall inhibition of peptide-bond formation (Ki* = 0.3 *μM*; S1 Fig, panel C). In addition, the slow *k*_off_ rate (0.64 min^-1^) provided prolonged residence time for compound **5** at the ribosome, a behavior potentially predicting good efficacy *in vivo* [28]. Although a direct comparison is not accurate, compound **5** is ~10-fold more potent than homodimers of CAM previously synthesized by Berkov-Zrihen et al. [25].

$$ln\frac{100}{100-x}=k_{obs\left(late\right)}t+\frac{\left[k_{obs\left(early\right)}-k_{obs\left(late\right)}\right]}{k_{eq}}\left(1-e^{k_{eq}t}\right)$$ (2)

Except for compound **8** that was inactive, all compounds shown in Fig 1, including CAM, exhibited a similar kinetic behavior to that adopted by compound **5**. The values of the kinetic parameters involved in the inhibition of the puromycin reaction by these compounds are summarized in Table 1. To examine if the effects seen are due to the presence of CAM dimers, we synthesized (see S1 Supplemental Procedures) three additional derivatives in which only one CAM molecule is attached to one molecule of linker, i.e. glutaric-CAM (**9**), adipoyl-CAM (**10**), and terephthaloyl-CAM (**11**), and tested them as inhibitors of peptide bond formation. Approximately, three- to six-fold lower inhibitory activity was recorded for these compounds (S1 Table), justifying the necessity of the presence of both CAM molecules for optimal potency. Consistently, compounds **9**–**11** were almost inactive in inhibiting the growth of *Staphylococcus aureus* or *E*. *coli* cells, at concentrations up to 100 μM. Taking into account that in a closed system, like the cell-free system used in our study, the inhibitory constant is an adequate metrics for differentiating compound potency [28], we used the *K*_i_* constant for ranking compounds **1**–**8**; *K*_i_* by definition (Eq 3) represents the overall inhibition constant engaged in both sequential reactions of the two-step mechanism shown in Fig 3C.

**Table 1:** Equilibrium and kinetic constants involved in the inhibition of AcPhe-puromycin synthesis by CAM dimers^a^.

Compound	*K*_*i*_ (μM)	*K*_i_* (μM)	*k*_on_/*k*_off_ ^b^	*k*_on_ (min^-1^)^c^	*k*_off_ (min^-1^)^c^
**CAM** ^d^	3.10 ± 0.30	0.88 ± 0.07	2.52 ± 0.44	2.29 ± 0.13	0.99 ± 0.04
**1**	2.40 ± 0.18	0.60 ± 0.05	3.00 ± 0.46	1.80 ± 0.16	0.60 ± 0.05
**2**	2.70 ± 0.25	0.75 ± 0.06	2.60 ± 0.44	1.70 ± 0.15	0.70 ± 0.05
**3**	4.50 ± 0.36	1.20 ± 0.09	2.75 ± 0.41	2.90 ± 0.23	1.00 ± 0.03
**4**	6.00 ± 0.54	1.87 ± 0.15	2.21 ± 0.38	2.14 ± 0.16	1.00 ± 0.04
**5**	1.40 ± 0.12	0.30 ± 0.03	3.67 ± 0.61	2.23 ± 0.10	0.63 ± 0.03
**6**	10.50 ± 0.90	2.30 ± 0.20	3.56 ± 0.56	2.80 ±0.25	0.80 ± 0.04
**7**	12.00 ± 1.20	2.80 ± 0.27	3.28 ± 0.59	2.89 ± 0.23	0.84 ± 0.06
**8** ^e^	-	-	-	-	-

^*a*^Data denote the mean ± S.E. values obtained from three independently performed experiments, with two replicates per experiment.

^*b*^The *k*_on_/*k*_off_ ratio was calculated through Equation: *k*_*on*_/*k*_*off*_ = *K*_*i*_/*K*_i_* − 1, [11].

^*c*^The individual values of *k*_on_ and *k*_off_ were calculated by nonlinear regression fitting of the kinetic data to Equation: $k_{eq}=k_{off}+k_{on}\frac{\frac{\left[I\right]}{K_{i}}}{1+\frac{\left[S\right]}{K_{S}}+\frac{\left[I\right]}{K_{i}}}$

^*d*^Data taken from Xaplanteri et al., [6]

^*e*^In the range of concentrations 1–20 μM, compound **8** was not active in inhibiting the puromycin reaction.

$$K_{i}^{*}=K_{i}\frac{k_{off}}{k_{on}+k_{off}}=\frac{\left[C\right]\left[I\right]}{\left[CI\right]+\left[C^{*}I\right]}$$ (3)

Accordingly, we estimated that compound **5** is 3-fold more potent than CAM. The rest of CAM dimers exhibited either comparable (compounds **1** and **2**), lower (compounds **3**, **4**, **6**, and **7**), or no activity (compound **8**). At a first glance, it is surmised that CAM dimers possessing a rigid aromatic linker of 6–7 Å, estimated by *in silico* analysis to equal the distance between the—NH- groups of CAM bound at the CAM1 and CAM2 sites, display the best activity in inhibiting the puromycin reaction. Among the CAM dimers tested, only compound **5** meets by the best balance of these structural properties. Compound **8** also bears a rigid linker. However, its length exceeds the ideal distance of 6–7 Å and therefore its ability to engage the CAM2 binding site is compromised. Compound **4**, possessing an aliphatic linker of similar length, functioned 6-fold less efficiently than compound **5**. In terms of the free energy of binding, compound **4** needs to pay a higher entropic cost upon binding than compound **5**. This is because multiple rotatable bonds in compound **4** allow more conformational degrees of freedom than those of compound **5**. A similar hypothesis can be adopted in explaining the low potency of compounds **3** and **6**. Compounds **1** and **2** that possess a short linker cannot simultaneously bind the catalytic crevice (CAM1) and the entrance to exit-tunnel (CAM2) and show a comparable activity to CAM.

### Structural characterization of the RI and R*I complexes by time-resolved footprinting analysis and MD simulations

The interactions between compound **5**, the most potent inhibitor of the puromycin reaction among the tested CAM dimers, and the *E coli* ribosome were dissected by time-resolved footprinting analysis. The behavior of compound **5** was compared to that of compound **4**, which is bearing a flexible linker of the same length. The footprinting analysis exploits the slow-binding character of the dimers and has been successfully applied in studying various slow-binding inhibitors of the PTase [11,29–31]. To overcome potential drawbacks resulting from protections caused by natural PTase substrates, naked 70S ribosomes were used instead of complex C. To footprint the RI complex, compounds **4** or **5** used in excess, and ribosomes were incubated at 25°C for 2 s, and then treated with chemical probes for 3 min to modify accessible nucleosides in 23S rRNA. Because the first step of binding, *R* + *I* ⇄ *RI*, equilibrates rapidly while the formation of R*I occurs slowly, the main product formed during such a short time interval was complex RI (>93%). To footprint the R*I complex, each dimer and ribosomes were incubated for 10 min, a time interval that is over than ten half-lives required for the attainment of the steady state. Because the isomerization constant, *k*_on_/*k*_off_, is at least 2.2 (see Table 1), most of the ribosomes added in the reaction mixture (>70%) were in the form of R*I complex at the end of this time interval.

Representative autoradiograms, achieved by primer extension analysis of the probed complexes, are shown in S2 Fig, alongside respective data obtained using CAM. The relative intensities of the bands of interest are presented in Table 2. As shown, the foot-printing patterns of RI complex for compounds **4** and **5** qualitatively resemble one another and do not significantly differ from that previously published for CAM [11,32–34]. This similarity suggests that both compounds occupy, via one of their symmetrical CAM portions, a pocket near the A-site of the PTase catalytic center (CAM1). Compound **5**, compared with **4**, exhibits stronger protections at nucleosides A2451 and U2506, a fact that is consistent with its lower *K*_i_ value (Table 1). However, larger differences were recorded when footprinting analysis was performed in the R*I complex; a protection seen at A2058 by compound **5** did not appear in the footprinting pattern of compound **4**. Moreover, all the protections due to compound **4** were generally weaker than those caused by **5**. This may be associated with the flexibility of the linker tethered by each compound.

**Table 2:** Relative reactivity of nucleosides in the central loop of Domain V of 23S rRNA, when a CAM dimer (I) binds to *E*. *coli* ribosomes (R) in the initial (RI) and the final (R*I) binding sites^a^.

	Compound 4			Compound 5		
23S rRNA residue	R	RI	R*I	R	RI	R*I
A2058	1	0.99 ± 0.07	0.90 ± 0.09	1	0.93 ± 0.06	0.32 ± 0.04 ^b^ ^,^ ^c^
A2059	1	0.94 ± 0.06	0.70 ± 0.07^b^ ^,^ ^c^	1	0.96 ± 0.08	0.33 ± 0.08 ^b^ ^,^ ^c^
A2062	1	0.88 ± 0.08^b^	0.67 ± 0.05^b^ ^,^ ^c^	1	0.80 ± 0.06 ^b^	0.40 ± 0.09 ^b^ ^,^ ^c^
A2451	1	0.67 ± 0.04^b^	0.77 ± 0.05^b^	1	0.40 ± 0.03^b^	0.41 ± 0.05^c^
G2505	1	0.43 ± 0.03^b^	0.44 ± 0.03^b^	1	0.38 ± 0.05^b^	0.35 ± 0.06^c^
U2506	1	0.70 ± 0.05^b^	0.70 ± 0.04^b^	1	0.47 ± 0.05^b^	0.33 ± 0.05^b^ ^,^ ^c^
U2609	1	1.00 ± 0.10	1.00 ± 0.07	1	1.00 ± 0.08	0.79 ± 0.08 ^b^ ^,^ ^c^

^*a*^Relative reactivity of nucleosides denotes the ratio between the normalized intensity of a band of interest and the normalized intensity of the homologous band in the control lane (R) (see also S2 Fig).

^*b*^Significantly different in relation to R (*P*<0.05).

^*c*^Significantly different in relation to RI (*P*<0.05).

**Fig 4:**
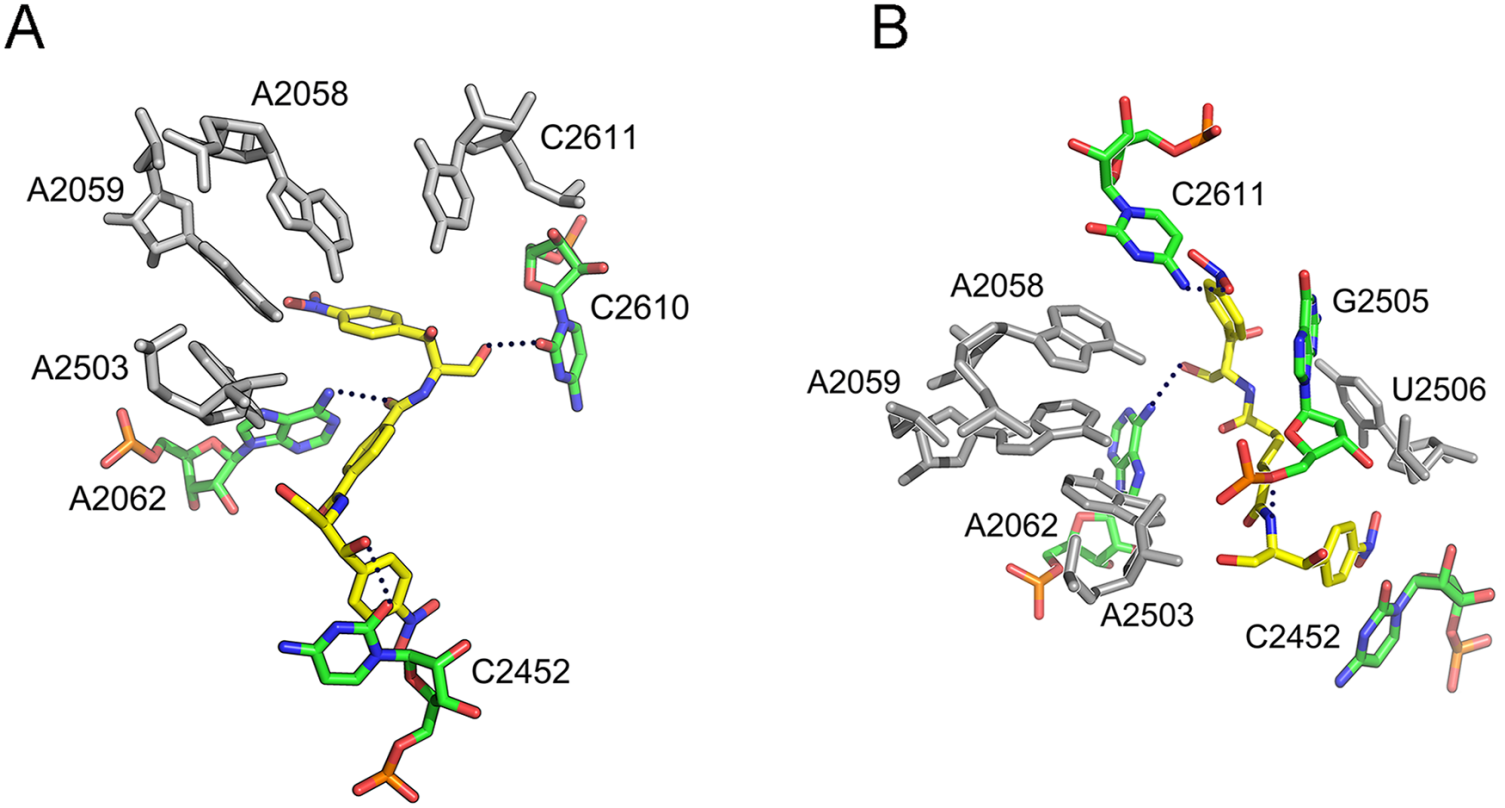
Binding positions of compounds 4 and 5 on the *E*. *coli* ribosome, as detected by Molecular Dynamics simulations. Compounds 4 and 5 have been docked into the 50S ribosomal subunit, by positioning one of their CAM moieties within the CAM crystallographic pocket [4]. (A) Binding position of compound 5 (yellow); hydrogen bonding with residues of the catalytic center is shown by black dots. Other residues of 23S rRNA placed adjacently to the binding pocket of 5 are ignored for clarity. (B) Binding position of compound 4 (yellow).

MD simulations confirmed that both compounds bind through one of their edges to the catalytic crevice (Fig 4). Compound **5** was additionally hydrogen bonded through its second CAM moiety to the 2OH- group of C2610, a fact compelling the *p*-nitrophenyl ring of this edge to bend and insert a hydrophobic crevice, formed by nucleosides A2058 and A2059 at the entrance to the exit tunnel (Fig 4A). Noteworthy, nucleoside C2610 has been considered as a part of a signal relay pathway linking the exit tunnel sensors to the PTase active site [35]. In contrast, compound **4** was revealed to bind C2611. Nevertheless, this interaction is not stable enough, nor it orientated the edge of compound **4** towards the A2058-A2059 crevice (Fig 4B). Due to technical limitations, certain interactions detected by MD simulations cannot be revealed by footprinting analysis; C2610 does not react with dimethyl sulfate (DMS), while C2611 is based paired with G2057 [36]. Binding models for the remaining CAM dimers, as generated by MD simulations, are presented in S3 Fig.

**Fig 5:**
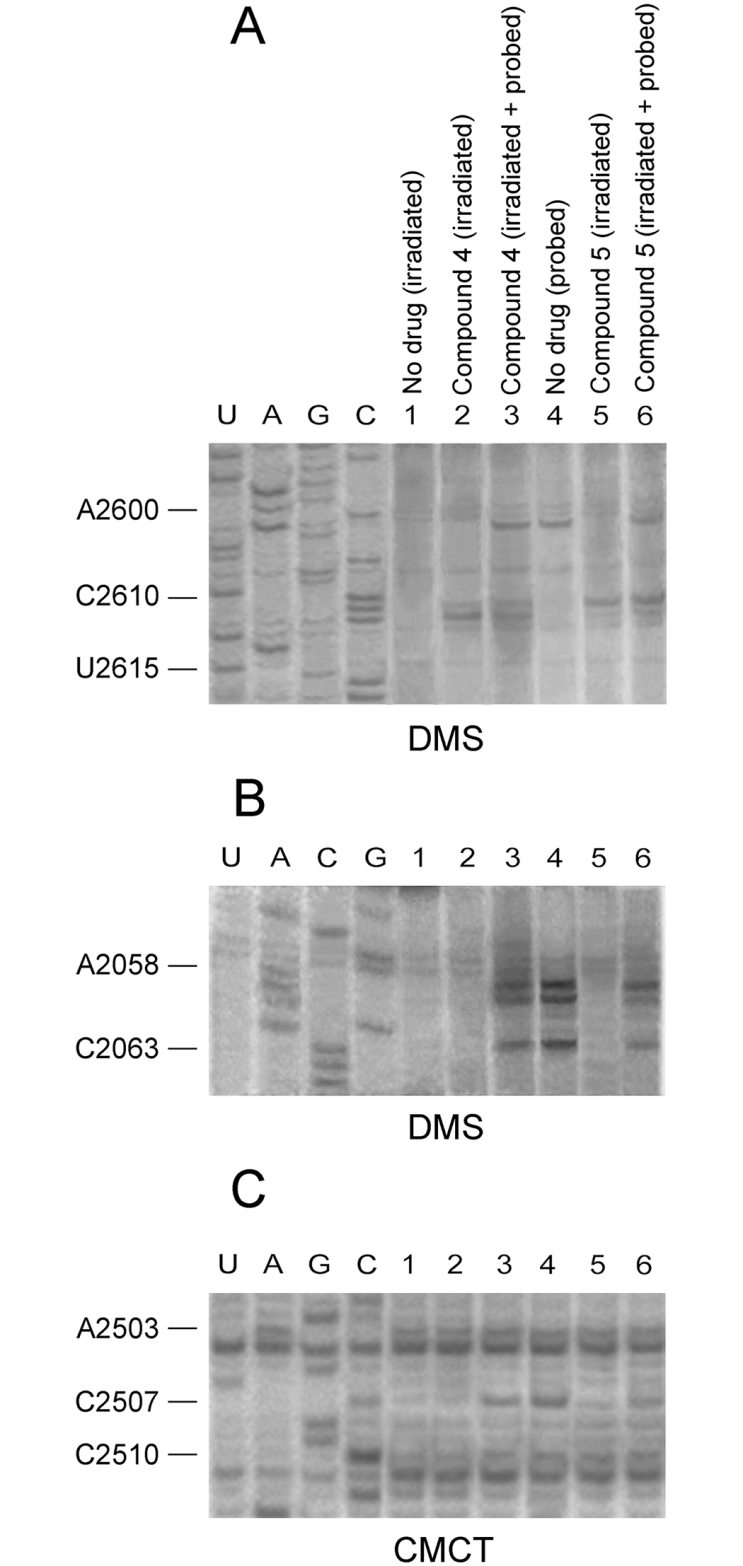
CAM dimer crosslinking at the entrance to the exit tunnel, upon UV-irradiation. Ribosomes from *E*. *coli* were irradiated with 365 nm light for 30 min (panels A-C), in the absence (lane 1) or the presence of compound 4 (lanes 2 and 3) or compound 5 (lanes 5 and 6). The irradiation products were analyzed by probing with DMS (panels A and B) or CMCT (panel C) and primer extension, before (lanes 3 and 6) or after discharging from excess CAM dimer (lanes 2 and 5). Probing and primer extension analysis were also applied to non-treated ribosomes (lane 4). Numbering of nucleosides for the sequencing lanes is indicated at the left. (A) Analysis of the A2600-U2615 region of 23S rRNA. (B) Analysis of the C2055-A2065 region (entrance to the exit tunnel) of 23S rRNA. (C) Analysis of the A2500-U2506 region (PTase catalytic center) of 23S rRNA.

To experimentally demonstrate that compounds **4** and **5** are capable of binding nucleosides C2611 and C2610, respectively, a crosslinking approach was applied. Specifically, a mixture of *E*. *coli* ribosomes together with either **4** or **5**, each added to the incubation mixture at concentration equal to 10×*K*_i_, were irradiated for 30 min with 365 nm light. Following purification, the irradiation products were analyzed by primer extension. As shown in Fig 5 (panels A and B), footprints of R*I complex having bound compound **5** mapped to nucleoside C2610, and less to nucleosides A2058 and A2059. Footprinting analysis of the whole irradiated mixture, before purification, indicated that compound **5** was firmly attached to the catalytic crevice of PTase, but did not form crosslinks with this region (Fig 5, panel C). In contrast, compound **4** crosslinked to C2611, without raising any modification signal in the A2058-A2059 region. It should be mentioned that the concentrations used for compounds **4** and **5** in this series of experiments were much lower (<60 μM) than those of CAM utilized previously by Long & Porse [12]. The most plausible explanation for this enhanced affinity is that binding of a dimer to the primary high-affinity site (catalytic crevice) facilitates targeting of a cryptic, low-affinity site (entrance to the exit tunnel) via the second edge of the homodimer. Such a site cannot be easily detected by kinetic analysis of CAM binding to the ribosome, due to its high *K*_i_ value (~300 μM) [12], and it is the first time that such a position is revealed by using the drug at micromolar concentrations.

### Antibacterial activity of the CAM dimers and correlation with inhibitory activity on the puromycin reaction

The antimicrobial potency of the synthesized CAM dimers was tested against a panel of Gram-negative and Gram-positive bacteria. Two laboratory strains of *E*. *coli* possessing the A2058G or A2503C mutations in 23S rRNA, the Rosetta(DE3)pLysS *E*. *coli* strain expressing the CAM acetyltransferase (*cat*) gene, and one clinical isolate of *Pseudomonas aeruginosa* exhibiting resistance against CAM due both to a constitutively expressed efflux system (MexAB-OprM) and an inducible efflux system (MexXY) [37] were included as representative CAM-resistant strains. In addition, wild-type *Enterococcus faecium*, *E*. *coli* and *Staphylococcus aureus* strains, along with two multi-drug resistant (MDR) *S*. *aureus* isolates (MRSA), were examined in our study. Compared to the parent antibiotic, none of the CAM dimers exhibited stronger inhibitory activity on the growth of wild-type *E*. *faecium*, *S*. *aureus* and *E*. *coli* cells (Table 3). However, better results were obtained when compound **5** was examined against the two multi-drug resistant MRSA isolates. The resistance of these isolates against a variety of antibiotics including methicillin, is reported in S2 Table. Interestingly, both MRSA isolates showed comparable susceptibility to compound **5** and CAM. Given that compound **5** causes less severe toxicity than CAM in human neutrophils (see below), this dimer seems to be a well promising lead candidate for the design of efficacious drugs against MDR Gram-positive bacteria. Intriguingly, compound **5** was approximately 2-fold more active than CAM in inhibiting the A2503C mutant and equivalent to CAM in inhibiting the growth of the A2058G mutant. Notably, previous studies have demonstrated that the incorporation of an interactive side chain into a macrolide scaffold can significantly improve the efficacy of this drug against bacteria that exhibit resistance conferred by changes in the PTase catalytic center [38].

**Table 3:** Determination of EC_50_ for CAM and CAM dimers, that indicates how much concentration of each compound is needed to produce 50% of the maximal inhibitory effect of that compound^a^.

EC_50_ (μM)										
	*E*. *faecium*	MRSA^b^	MRSA^b^	*S*. *aureus*	*E*. *coli*	*E*. *coli* ^c^	*E*. *coli* ^c^	*E*. *coli* ^d^	*E*. *coli* ^e^	*P*. *aeruginosa* ^f^
Compound	(GRE5152)	(GRE2272)	(GRE2691)	(WT)	(WT)	(A2058G)	(A2503C)	(Δ*tolC*)	Rosetta(DE3)pLysS	(GRE5288)
**CAM**	3.5 ± 0.3	8.4 ± 0.7	12.8 ± 1.5	2.9 ± 0.1	4.4 ± 0.2	15.9 ± 1.1	24.9 ± 2.5	2.2 ± 0.1	114.8 ± 16.3	25.6 ±2.2
**1**	>200	>200	>200	32.2 ± 1.0	>200	>200	>200	>200	>200	>100
**2**	>200	>200	>200	44.4 ± 2.9	>200	>200	>200	>200	>200	>100
**3**	69.8 ± 14.8	20.8 ± 2.5	27.2 ± 3.4	17.4 ± 1.3	52.4 ± 6.8	47.3 ± 6.9	68.1 ± 13.8	43.7 ± 5.2	65.1 ± 6.6	42.4 ± 8.7
**4**	99.8 ± 46.9	>200	76.9 ± 8.6	28.3 ± 1.8	83.6 ± 6.2	97.0 ±3.3	>100	98.0 ± 16.3	>200	72.6 ± 13.7
**5**	40.9 ± 3.8	16.9 ± 4.4	24.0 ± 3.1	15.4 ± 1.9	25.3 ± 1.9	18.0 ± 1.5	16.4 ± 1.5	23.7 ± 1.5	23.5 ± 1.3	15.2 ± 1.3
**6**	>200	>200	>200	49.6 ± 5.4	>200	>200	>200	>200	>200	>100
**7**	>200	>200	>200	72.7 ± 11.8	>200	>200	>200	>200	>200	>100
**8**	>200	>200	>200	>200	>200	>200	>200	>200	>200	>100

^*a*^Data represent the mean±SE values obtained from three independently performed experiments, with two replicates per experiment. EC_50_ values were determined by nonlinear regression fitting of the observed optical density values (Y) into Hill Equation, $y=min+\frac{max-min}{1+{\left(\frac{x}{EC_{50}}\right)}^{-n}}$ (see Materials and Methods).

^*b*^Methicillin-resistant *S*. *aureus* (MRSA) isolates belong to the ST80 clone and exhibit multi drug resistance behavior (see also S2 Table)

^*c*^ *E*. *coli* TA531 cells lacking chromosomal *rrn* alleles, but containing pKK35 plasmids that possess wild-type 23S rRNA display the same EC_50_ value for each drug, like those of wild-type (WT) *E*. *coli* K12 cells. However, when pKK35 plasmids possess mutated 23S rRNA (A2058G or A2503C), the cells are resistant to CAM because of target mutation [14,15].

^*d*^ Deletions in *tolC* gene result in an increased sensitivity of *E*. *coli* to a wide range of antibiotics, including CAM [39].

^*e*^Rosetta(DE3)pLysS *E*. *coli* cells express the *cat* gene that encodes CAM acetyltransferase, an enzyme that inactivates CAM [40] (see also Fig 6).

^*f*^ *P*. *aeruginosa* exhibits reduced susceptibility to CAM, in part due to the intrinsically expressed MexAB-OprM efflux system, and additionally to an inducible by CAM efflux system (MexXY) [37].

**Fig 6:**
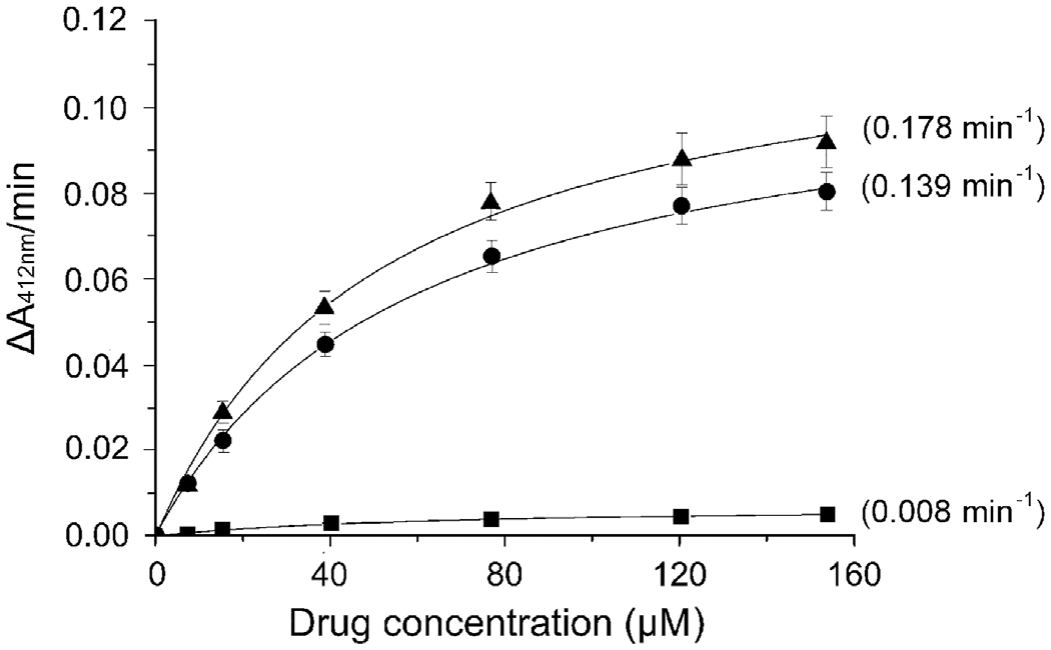
Kinetic analysis of the CAM acetyltransferase reaction using CAM or compounds 4 and 5 as substrates. The reaction was carried out in 3 ml of 94 mM Tris/HCl pH 7.8, containing 0.083 mM 5,5’-dithio-bis(2-nitrobenzoic acid), 0.16 mM acetyl coenzyme A, 25 units CAM acetyltransferase, and either CAM (●), compound 4 (▲), or compound 5 (■) at the concentrations indicated. The product of the enzymatic reaction, coenzyme A, reacted with 5,5’-dithio-bis(2-nitrobenzoic acid) to yield 5-thio-2-nitrobenzoate which absorbs at 412 nm, with a micromolar extinction coefficient equal to 0.0136. The V_max_ and K_m_ values were determined by fitting the substrate concentrations [S] and the obtained ΔA_412nm_/min (V_o_) values into equation *V*_0_ = *V*_*max*_[*S*]/(*K*_*m*_ + [*S*]). The obtained V_max_ values were divided by 0.0136 to convert their units in μM·min^-1^ (http://www.sigmaaldrich.com/technical-documents/protocols/biology/enzymatic-assay-of-chloramphenicol-acetyltransferase.html). The ratio V_max_/K_m_ for each curve is given in parenthesis.

Compared to CAM, compound **5** displayed a five-fold higher activity in inhibiting the growth of Rosetta(DE3)pLysS *E*. *coli* cells expressing the *cat* gene. This was a truly interesting finding, immediately raising the question of how this would work from a biochemical point of view. Expression of CAM acetyltransferase, encoded by the *cat* gene, is a major mechanism by which bacteria become resistant to CAM. This enzyme catalyzes transfer of the acetyl moiety from acetyl coenzyme A to CAM [40]. The O-acetoxy derivatives of CAM fail to act as antibiotics, because they do not bind to bacterial ribosomes. Therefore, we tested compounds **4** and **5** against the purified enzyme, by calculating the ratio V_max_/K_m_. These calculations allowed the direct evaluation of the capacity of each compound to behave as acceptor of acetyl groups. As shown in Fig 6, compound **4** behaves like CAM as substrate of CAM acetyltransferase, while compound **5** was almost inactive. However, it should be noted that the number of available hydroxyl groups in both CAM dimers is twice that of CAM. Therefore, they should confer double initial velocity to the reaction at low substrate concentration, if they were just as efficient as CAM.

The preferential activity of CAM dimers against the growth of *S*. *aureus* cells than *E*. *coli* cells is the first evidence that penetration of the outer cellular membrane may be a significant limitation in the efficacy of CAM dimers as antibiotics. It is known that CAM gains access to the periplasm through pore-forming porins [19], and that utilization of the porin pathway by antibiotics depends on the molecular dimensions of the drugs [41]. Therefore, we suggested that CAM dimers are too large for effective diffusion through porins. The second evidence was provided when the antimicrobial activity of CAM dimers was correlated with their ability to inhibit *in vitro* peptide-bond formation. By using IC_50(puro)_ as a criterion of the efficiency of compounds in targeting the ribosome, where IC_50(puro)_ is defined as the compound concentration causing 50% inhibition in peptide-bond formation at the presence of 2 mM puromycin, and calculating the ratio EC_50(cell growth)_/IC_50(puro)_, we realized that the value of this ratio is much lower for CAM than for any CAM dimer (S3 Table). This suggests that CAM dimers are prone to transport limitations. It should be kept in mind that a second bacterial barrier, the plasma membrane, may also contribute in obstructing CAM dimers from accumulating into the cells. There are more than seven efflux systems in *E*. *coli* that can pump out toxic compounds, such as antibiotics, detergents, organic solvents etc [39]. An important efflux system in *E*. *coli* is the AcrAB-TolC multidrug resistance tripartite pump [42]. Deletions in *acrAB* and/or *tolC* genes result in an increased sensitivity of *E*. *coli* to a wide range of antibiotics, including CAM [39]. To investigate the effect of this efflux system on the intracellular accumulation of our compounds, we determined the EC_50_ values against an *E*. *coli* strain BL21 DE3 lacking the *tolC* gene that codes TolC, the outer membrane component of the AcrAB-TolC efflux pump. We observed that this efflux system does not appear to affect the antibacterial activity of CAM dimers, as the EC_50_ values regarding this strain were similar to those of wild-type *E*. *coli* (Table 3). Notably, *E*. *coli* BL21 DE3 (Δ*tolC*) strain was approximately 2-fold more sensitive to CAM than wild-type *E*. *coli* (Table 3). In contrast, another Gram-negative bacterium, *P*. *aeruginosa*, possessing both inducible and constitutively expressed efflux pumps showed 6-fold higher EC_50_ value for CAM than wild-type *E*. *coli*, maintaining the EC_50_ values for compounds **3**, **4**, and **5** almost unchanged. This means that CAM dimers are neither recognized by the constitutively expressed MexAB-OprM efflux system, nor induce the MexXY efflux system of this bacterium [37].

### Toxicity of CAM dimers against Human peripheral blood cells and leukemic cell lines

**Fig 7:**
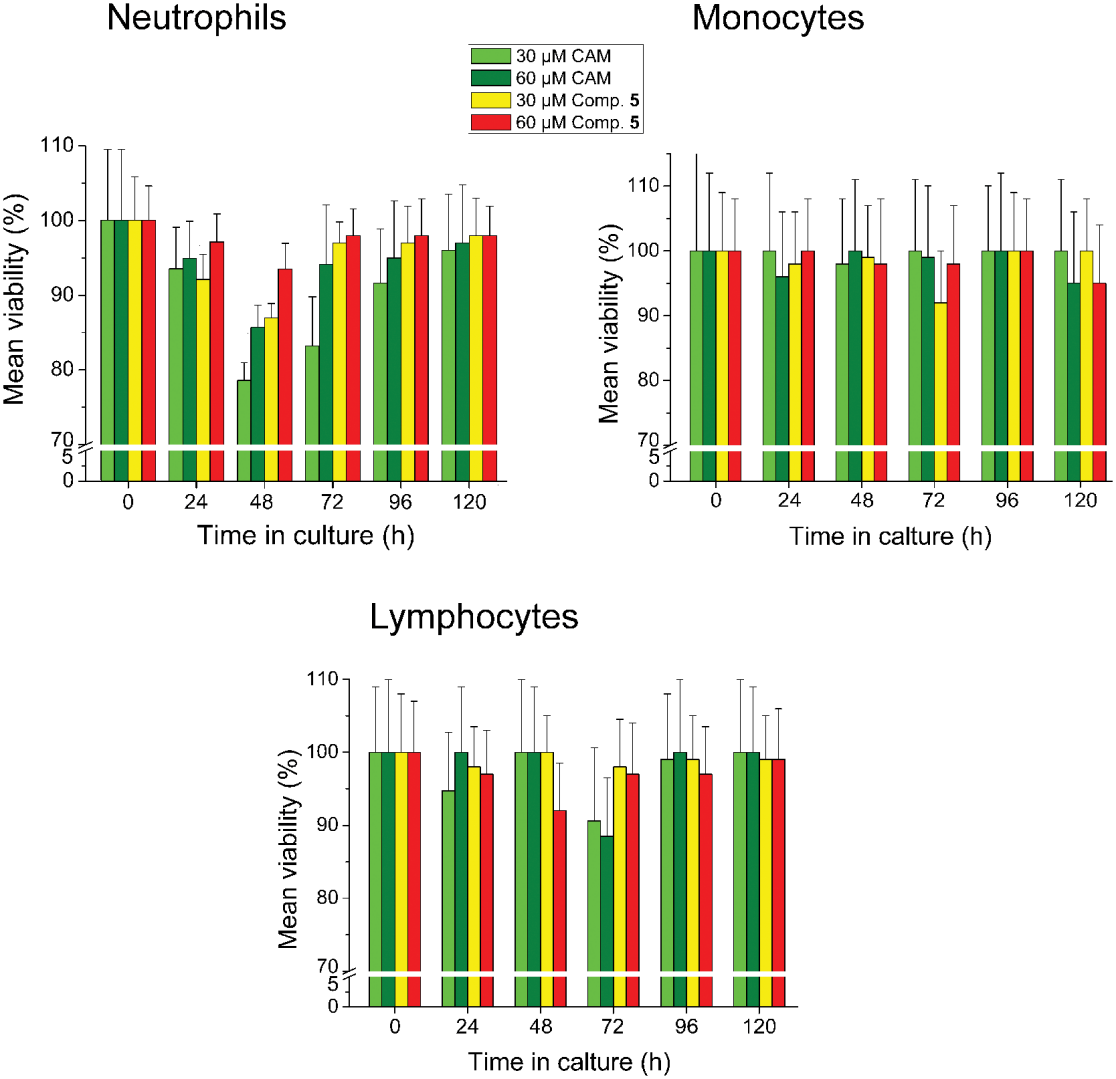
Toxicity assays in human peripheral blood cells. Peripheral blood was collected in EDTA-coated tubes from 5 healthy volunteers (age range: 25–30 years). Concentration was adjusted to 1.8×10^9^ cells/L using RPMI-1640 medium containing 1% penicillin/streptomycin. Cells were cultured in triplicate in the presence or the absence of 30 or 60 μM CAM or compound 5, under a humidified 5% CO_2_ atmosphere for 5 days, at 37°C. Cultures were counted daily by a CELL-DYN 3700 Hematology Analyzer and values were expressed as a percentage of cells measured in controls.

Accumulating evidence has shown that CAM causes adverse effects to the hematopoietic system [21,43–45]. This prompted us to test the CAM dimers for potential toxicity against human peripheral blood cells and leukemic cell lines. Compound **5**, the most potent member of the synthesized CAM dimers, displayed a mild and transient toxicity on neutrophils during a 120 h exposure of blood cells to this compound, peaked at 48 h (Fig 7). Nevertheless, this toxicity was less severe when compared with that caused by CAM. Toxicity of compound **5** against other types of leukocytes was negligible.

**Fig 8:**
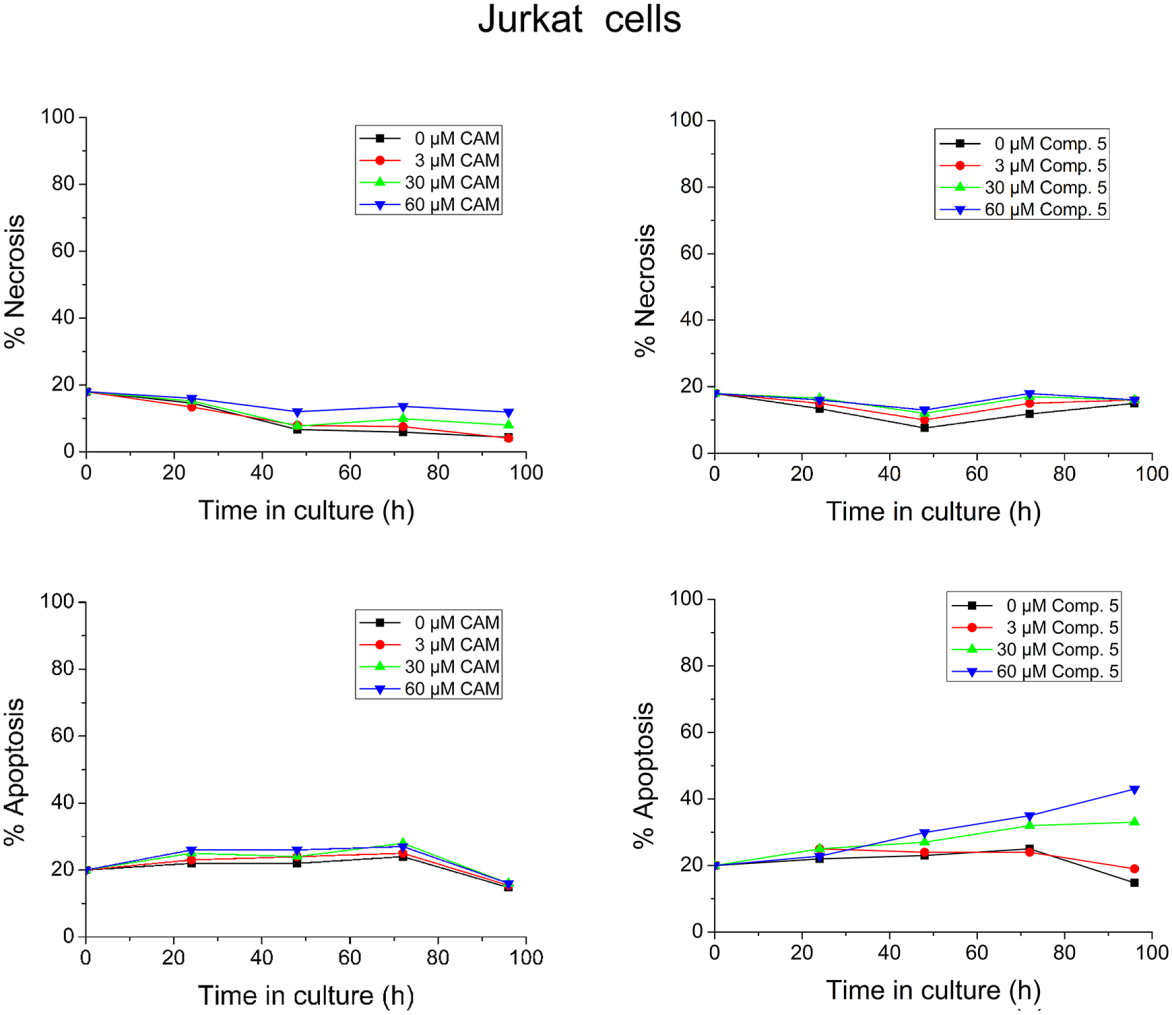
Toxicity assays in Jurkat cells. Jurkat cells were adjusted to 1×10^9^ cells/L in RPMI-1640 medium containing 1% Penicillin/Streptomycin and 10% fetal bovine serum. The cells were grown in triplicate in the presence or absence of compound 5 at the indicated concentrations for 4 days at 37°C, under a humidified 5% CO_2_ atmosphere. CAM was used as a reference compound. For cell necrosis and apoptosis assays, samples (10^6^ cells) were collected daily and determined by flow cytometry. Apoptotic and necrotic cells were expressed as a percentage of total cells.

The toxicity effects of compound **5** on leukemic cell lines were tested, using HS-Sultan, Jurkat and U937 cells. Preliminary records, produced by counting daily the cells in a CELL-DYN 3700 Hematology Analyzer, showed that Jurkat cells grew exponentially at all the tested concentrations of compound **5**; however, the rate of growth was reduced proportionally to the concentration of compound **5** (S4 Fig). In contrast, HS-Sultan or U937 cells were insensitive to compound **5**. Therefore, the effect of compound **5** on Jurkat cells was further studied by flow cytometric analysis. The results showed that compound **5** at 60 μM failed to induce necrosis, but did induce 43% apoptosis to Jurkat cells, expressed as a percentage of total cells (Fig 8). In comparison, CAM at 60 μM did not induce any necrosis/apoptosis effect to these cells under the same conditions of treatment. This different response confers compound **5** a comparatively significant advantage over CAM, since the anti-apoptotic behavior of CAM plays a critical role in CAM-induced leukemogenesis by allowing proliferating cells to continuously survive [21].

### Conclusions

To explore the existence and utilization of multiple binding sites of CAM within the central loop of domain V of 23S rRNA, we constructed eight homodimers of CAM, tethered via a linker of varying length and flexibility. Compared to CAM, three of the CAM dimers inhibited the AcPhe-puromycin synthesis, a model reaction for peptide-bond formation, more efficiently. Footprinting and crosslinking analysis, combined with computational modeling, revealed that the enhanced binding affinity exhibited by these constructs resulted from their unique architecture and ability to recognize multiple binding sites within the ribosome’s three-dimensional structure. It was realized that multiple interactions synergize in order to enhance the apparent affinity and lead to a prolonged residence time of the constructs at their targets. Specifically, dissection of the mechanism of action of compound **5** binding to the ribosome allowed us to verify a kinetically cryptic binding site of CAM at the entrance to the exit tunnel and gave us the opportunity to clarify previous uncertainties related to the number and exact localization of CAM binding sites in the ribosome. The remarkable *in vitro* inhibitory activity of compound **5** on bacterial protein synthesis, combined with its ability to bypass some resistance mechanisms, its low toxicity against human peripheral blood cells and promising activity against human T-leukemic cells provide the impetus to further improve its design. Realizing the serious problems met in internalizing CAM dimers into bacterial cells, future efforts will focus on strengthening the capacity of these compounds in penetrating the outer and plasma membrane barriers.

## Materials and Methods

### Materials, bacterial strains, peripheral blood samples, leukemic cell lines, biochemical preparations, and instrumentation

CAM free base [D-(-)*threo*-1-(*p*-nitrophenyl)-2-amino-1,3-propanediol]), tRNA^Phe^ from *E*. *coli*, dimethyl sulfate (DMS), DMS stop solution, puromycin dihydrochloride, and tRNA^Phe^ from *E*. *coli* were from Sigma-Aldrich. 1-Cyclohexyl-3-(2-morpholinoethyl)-carbodiimide metho-*p*-toluene sulfate (CMCT) and kethoxal were purchased from Fluka Biochemicals and MP Biomedicals, respectively. AMV reverse transcriptase was supplied by Roche, dNTPs by HT Biotechnology, and ddNTPs by Jena Bioscience. L-[2,3,4,5,6 -^3^H] Phenylalanine was from Amersham Biosciences and [α-^32^P] ATP from Izotop. The HS-Sultan (Burkitt’s lymphoma) cell line was purchased from the European Collection of Cell Cultures, Salisbury, UK, while the Jurkat (T-cell acute lymphoblastic leukemia) and U937 (histiocytic lymphoma) cell lines were supplied by the American Type Culture Collection Manassas, USA. Peripheral blood was collected in EDTA-coated tubes from 5 healthy volunteers (age range: 25–30 years, members of the research personnel of the Division of Hematology) following the principles expressed in the Declaration of Helsinki and the data were analyzed anonymously. Volunteers gave verbal consent to use their blood samples in the *in vitro* experiments of this work and to publish the results obtained. Human experimentation guidelines were submitted to and approved by the Internal Review Board and the Scientific Advisory Committee of Patras University Hospital (PUH). Consents were verbal because the blood donors were members of the research personnel of the Division of Hematology, AM included, who participated in the study by assisting the performance of the flow cytometry assays. Before blood collection, the volunteers were informed in detail about the intended use of their blood samples and the way of publishing the obtained results. The Advisory Committee of PUH abiding by the Helsinki Declaration on ethical principles for medical research involving human subjects approved this consent procedure. Ac[^3^H]Phe-tRNA^Phe^ charged to 80% and a post-translocation complex of poly(U)-programmed ribosomes (complex C) from *E*. *coli* K12, bearing tRNA^Phe^ at the E-site and Ac[^3^H]Phe-tRNA at the P-site were prepared, as previously described [46]. The percentage of active ribosomes in AcPhe-tRNA binding was 75%.

Melting points were determined with a Buchi SMP-20 apparatus and are uncorrected. IR spectra were recorded as KBr pellets on a Perkin Elmer 16PC FT-IR spectrophotometer. ^1^H NMR spectra were obtained at 400.13 MHz and ^13^C NMR spectra at 100.62 MHz on a Bruker DPX spectrometer. Chemical shifts are reported in *δ* units, parts per million (ppm) downfield from TMS. Electron-spray ionization (ESI) mass spectra were recorded at 30V, on a Micromass-Platform LC spectrometer using MeOH as solvent. Analytical HPLC was used to determine the purity of final products, confirming ≥ 95% purity. Analytical RP-HPLC was performed on a Waters system (2695 Alliance). Elution of the compounds was determined from the absorbance at 254 nm (Waters 2996 Photodiode array detector). Compound purity was assessed using a LiChrospher C8 column (5 μm, 125 x 4.0 mm) and a linear gradient of 5%-60% acetonitrile (containing 0.05% TFA) in water (containing 0.05% TFA) over 20 min at a flow rate of 1 ml/min. Flash column chromatography (FCC) was performed on Merck silica gel 60 (230–400 mesh) and TLC on 60 Merck 60F_254_ films (0.2 mm) precoated on aluminium foil. Spots were visualized with UV light at 254 nm and charring agents. All solvents were dried and/or purified according to standard procedures prior to use. All reagents employed in synthesis were purchased from either Aldrich or Alfa-Aesar. CLB and the required dicarboxylic acids, glutaric anhydride and terephthaloyl dichloride were obtained from Aldrich. The synthesis of 1,4-phenylenediacrylic acid and compounds **1**–**11** as well as physical and spectra data for the synthesized compounds are presented in S1 Supplemental Procedures.

### Inhibition of peptide-bond formation by CAM dimers

The puromycin reaction, i.e. the reaction between complex C and excess puromycin (S), was carried out at 25°C in buffer A [100 mM Tris-HCl pH 7.2, 6 mM (CH_3_COO)_2_Mg, 100 mM NH_4_Cl and 6 mM 2-mercaptoethanol]. Under these conditions, the puromycin reaction obeys

$$\text{C }+\text{ S }\overset{{\text{K}}_{\text{S}}}{⇄}\text{ CS}\overset{{\text{k}}_{\text{cat}}}{\to }\;{\text{C}}^{\prime }\;+\text{ S}$$

pseudo-first-order kinetics and was analyzed as previously described [11].

In the presence of CAM dimers, biphasic semi-logarithmic time plots were obtained. The slope of the straight line through the origin was seen as the value of the apparent rate constant, *k*_obs(early)_, at the early phase of the reaction. Similarly, the slope of the second straight line was taken as the apparent rate constant, *k*_obs(late)_, at the late phase of the puromycin reaction.

### Time-resolved binding of CAM dimers to *E*. *coli* ribosomes and characterization of RI and R*I complexes by footprinting analysis

70S ribosomes from *E*. *coli* (100 nM) were incubated either alone or with each CAM dimer at concentration equal to 50×*K*_i_ in 100 μl of buffer B [Hepes/KOH, pH 7.2, 6 mM Mg(CH_3_COO)_2_, 100 mM NH_4_Cl, and 5 mM dithiothreitol] at 25°C, either for 2 s (RI probing) or longer than 10×*t*_1/2_ min (R*I probing). Chemical modification of complexes RI and R*I with DMS, kethoxal, or CMCT, primer extension analysis, and gel electrophoresis of the primer extension products were performed as previously described [47]. The primers used were complementary to the sequences 2102–2119, 2561–2578, and 2680–2697 of 23S rRNA to scan primarily domain V, provided that one of the CAM units in CAM dimers binds within the catalytic center of PTase and the size of each CAM dimer does not exceed 30 Å. Quantitative scanning of the gels, and normalization of the band intensities were made as previously shown [11]. Values indicated in Table 2 denote the ratio between the normalized intensity of a band of interest and the normalized intensity of the corresponding band in the control lane (ribosomes non-treated with CAM or CAM dimers).

### Crosslinking of CAM dimers to *E*. *coli* ribosomes

70S ribosomes from *E*. *coli* (100 nM) were incubated either alone or with compound **4** or **5** at concentration equal to 50×*K*_i_ in 100 μl of buffer B [Hepes/KOH, pH 7.2, 6 mM Mg(CH_3_COO)_2_, 100 mM NH_4_Cl, and 5 mM dithiothreitol] at 25°C, for 5 min. Following formation of R*I complexes, the samples were irradiated at 365 nm, for 30 min, in a Vilber Lourmat UV Cabinet (VL-215.LC-30W) light source. The light source was placed ~5 cm over a microtiter tray containing the sample on an ice-water bath. Half of the sample was probed with DMS or CMCT, and then analyzed by primer extension as shown above, while the other half was extracted with phenol, phenol-chloroform (1:1), and chloroform, followed by ethanol precipitation to remove non-crosslinked agents. The isolated rRNA was then subjected to primer extension analysis.

### Molecular Dynamics simulations

3D models for compounds **1** to **8** and their parameterization for the CHARMM Force field were achieved, as previously described [11], starting with the 3D structure of CAM derived from crystallographic data (PDB: 3OFC). The CAM dimers were docked into the 50S ribosomal subunit structure, by positioning one of their CAM moieties within the drug crystallographic pocket. All groups of 50S subunits in a distance of 10 Å around CAM dimers were selected, solvated with TIP3 water molecules, and then neutralized with sodium ions using the VMD program [48].

All systems derived as above were energy minimized and then subjected to canonical ensemble Molecular Dynamics (MD) simulations for 10 ns at 300K, with Particle Mesh Ewald (PME) algorithm and rigid bonds assigned using the NAMD software [49]. During MD simulations, all nucleic acid backbone atoms were positionally restrained. Finally, an average structure over the last 100 frames of each simulation trajectory was energy minimized and used for further analysis. An H-bond was considered as existing, if hydrogen donor and acceptor atoms were closer than 0.35 nm and the angle between the line connecting these atoms and the hydrogen bond was lower than 30°. All molecular visualizations were produced with the PyMOL Molecular Graphics System, Version 1.5.0.4 Schrödinger, LLC.

### Biological evaluation of CAM dimers in bacterial cells containing wild-type or mutant ribosomes

The antibacterial activity of CAM dimers was assessed in CAM-sensitive *E*. *faecium*, *S*. *aureus* and *E*. *coli* strains, as well in two CAM-resistant strains of *E*. *coli* lacking chromosomal *rrn* alleles, but containing pKK35 plasmids possessing mutated 23S rRNA (A2058G or A2503C), kindly offered by Prof. A.S. Mankin (University of Illinois). *E*. *coli ΔtolC* strain BL21 DE3 with impaired AcrAB-TolC, a proton-dependent MDR efflux pump causing multidrug resistance, was offered by Dr D.N. Wilson (University of Munich) and included in our study to test if this mechanism of resistance affects the efficacy of CAM dimers. In addition, two methicillin-resistant *S*. *aureus* (MRSA) isolates belonging to the ST80 clone and one *P*. *aeruginosa* clinical isolate, all exhibiting multi drug resistance behavior (S2 Table), were kindly offered by Prof. I. Spiliopoulou (National Reference Laboratory for Staphylococci, School of Medicine, University of Patras, Greece). Finally, *E*. *coli* strain Rosetta(DE3)pLysS, containing a chloramphenicol resistant gene in the pLysS plasmid, was purchased from Novagen and was also included in our study. Briefly, *S*. *aureus* or *E*. *coli* cells (200 μl of a 0.700 OD_560_ preculture) containing wild-type or mutant ribosomes were added in 3.8 ml of LB (Luria-Bertani) medium and grown at 37°C in the presence or absence of CAM or CAM dimers until the optical density of the control culture (grown in the absence of drug) reached the value 0.700 at 560 nm. For *E*. *faecium* and *P*. *aeruginosa* cultures, TSB (Tryptic Soy Broth) medium was used, instead of LB medium, in order to achieve exponential doubling times between 25 and 35 minutes. From dose-response curves, the half-maximal effective concentration (EC_50_) for each compound and strain was estimated. EC_50_ represents the molar concentration of a compound that produces 50% of the maximal possible effect [50]. The EC_50_ values were mathematically determined by non linear regression fitting of the observed culture optical-density values, expressed as the percentage of 0.700 (*y*), into Hill Equation,

$$y=min+\frac{max-min}{1+{\left(\frac{x}{EC_{50}}\right)}^{-n}}$$

where *min* and *max* are the lowest and highest observed values of the culture optical density, respectively, *x* the concentration of the tested compound, and *n* the Hill coefficient that represents the largest absolute value of the curve slope. EC_50_ is equal to the *x*- value of the sigmoid’s midpoint. Fitting was performed using the Four Parameter Logistic Curve of the SigmaPlot Program Version 11.0 (Systat Software, Inc) for Exact Graphs and Data Analysis.

### CAM acetyltransferase assay

The activity of CAM acetyltransferase (CAT) employing CAM or CAM dimers as acetyl-acceptor substrates was assayed by using purified enzyme from *E*. *coli* (Sigma-Aldrich), following the manufacturer’s protocols (http://www.sigmaaldrich.com/technical-documents/protocols/biology/enzymatic-assay-of-chloramphenicol-acetyltransferase.html).

### Toxicity assays in Human peripheral blood cells and leukemic cell lines

Peripheral blood was collected in EDTA-coated tubes from 5 healthy volunteers (age range: 25–30 years). Cell concentration was adjusted to 5×10^8^ cells/l using RPMI-1640 medium (GIBCO BRL) containing 1% penicillin/streptomycin. Cells were cultured in triplicate under a humidified 5% CO_2_ atmosphere for 5 days, at 37°C, in the absence (control cultures) or the presence of CAM or CAM dimers. Counting of cells was performed daily in a CELL-DYN 3700 Hematology Analyzer (Abbott, USA) and values were expressed as a percentage of cells measured in control cultures.

Human leukemic cell lines, HS-Sultan (Burkitt’s lymphoma), Jurkat (T-cell acute lymphoblastic leukemia), and U937 (histiocytic lymphoma), were adjusted to 1×10^9^ cells/l in RPMI-1640 medium supplemented with 10% fetal bovine serum and 1% penicillin/streptomycin. The cells were grown in triplicate in the absence (control cultures) or presence of CAM or CAM dimers for 4 days at 37°C, under a humidified 5% CO_2_ atmosphere. The medium was changed daily; CAM or CAM dimer addition was repeated after medium change. Aliquots were collected daily and counted in a CELL-DYN 3700 Hematology Analyzer. For cell necrosis and apoptosis assays, samples (10^6^ cells) were collected and determined using the Annexin V-PE Apoptosis Detection Kit I (BD Pharmingen) for flow cytometry [51], according to the manufacturers’ instructions. Flow cytometry data were analyzed using the FlowJo flow cytometry analysis software. Necrotic and apoptotic cells were expressed as a percentage of total cells.

### Statistical analysis

All data presented in the Figures and Tables denote the mean values obtained from three independently performed experiments, with two replicates per experiment, and are expressed as means±standard error. Significant differences between mean values were measured at p < 0.05 by the F-Scheffe test (SPSS program 20.0 for Windows).

## Supporting Information

S1 Fig Kinetic plots for the AcPhe-puromycin synthesis in the presence or absence of compound 5. (DOCX)

S2 Fig Protections against chemical probes in nucleotides of the central loop of domain V of 23S rRNA, caused by binding of CAM or CAM dimers (compounds 4 and 5) to *E*. *coli* ribosomes. (DOCX)

S3 Fig Binding positions of CAM dimers in the *E*. *coli* ribosome, as detected by Molecular Dynamics Simulations. (DOCX)

S4 Fig Toxicity assays in Human leukemic cell lines. (DOCX)

S1 Supplemental Procedures General experimental procedure for the synthesis of compounds 1–8. Synthesis of 1,4-phenylenediacrylic acid, ^1^H- and ^13^C-NMR spectra and RP-HPLC chromatograms of CAM dimers 1–8. General procedure for the synthesis of compounds 9 and 10. Synthesis of compound 11. ^1^H- and ^13^C-NMR spectra of compounds 9–11. Supplemental references.
(DOCX)

S1 Table Kinetic parameters of the puromycin reaction carried out in the presence of CAM attached to linkers indicated by red. (DOCX)

S2 Table Bacterial strains and mechanism of resistance or hypersensitivity to antibiotics. (DOCX)

S3 Table Determination of the ratio EC_50(cell growth)_/IC_50(puro)_ in wild-type *E*. *coli* for CAM and CAM dimers. (DOCX)
