# Supplementary material for: Synthesis and Evaluation of Chloramphenicol Homodimers: Molecular Target, Antimicrobial Activity, and Toxicity against Human Cells
Source: PLoS One. 2015 Aug 12;10(8):e0134526. doi: 10.1371/journal.pone.0134526 (PMC4533973; doi:10.1371/journal.pone.0134526)
Supplement: S1 Fig — (DOCX) [file pone.0134526.s001.docx]

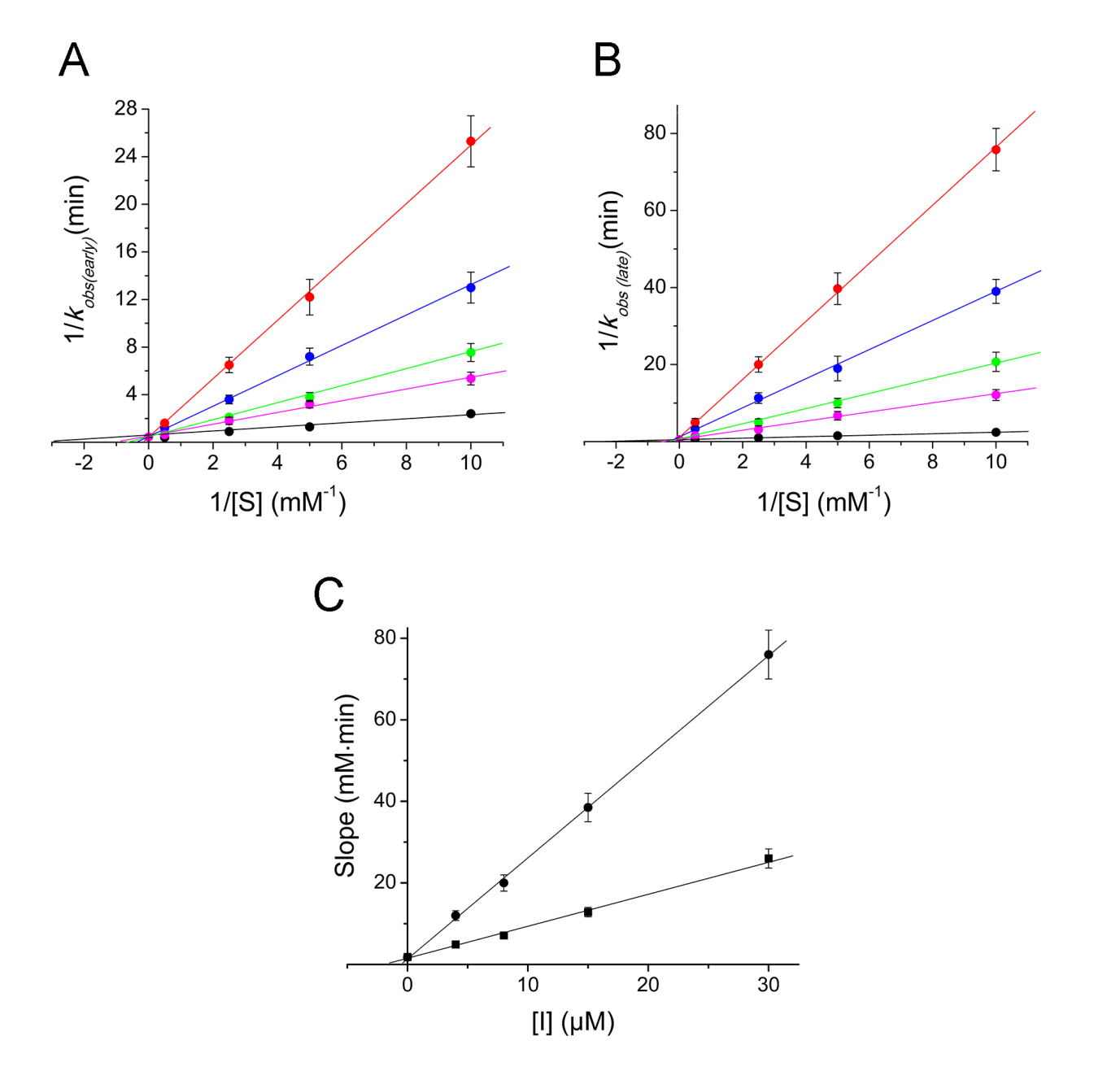


**S1 Fig. Kinetic plots for the AcPhe-puromycin synthesis in the presence or absence of compound 5**. (A) and (B), Double-reciprocal plots; kinetic data were collected from the early and the late phases of semi-logarithmic plots, respectively, such as those presented in in the text (Fig. 2A). Compound **5** concentrations are denoted as follows: 0 μΜ (black), 4 μΜ (magenta), 8 μΜ (green), 15 μΜ (blue), and 30 μΜ (red). (C), Slope replots (slopes of the double-reciprocal plots versus compound **5** concentration). The slope values were estimated from the plots shown in (squares) panel A or (circles) panel B. The plots presented in panels A, B, and C denote that the inhibition at both phases is of competitive type and that only one ribosomal binding site is involved at each phase of the inhibition process. The straight lines shown in panel C, when extrapolated, meet the horizontal axis of the plot at a point pertaining to the inhibition constant, which is for the early phase of the reaction *K*_i_ and the late phase of the reaction *K*_i_^*^.
