# Supplementary material for: Synthesis and Evaluation of Chloramphenicol Homodimers: Molecular Target, Antimicrobial Activity, and Toxicity against Human Cells
Source: PLoS One. 2015 Aug 12;10(8):e0134526. doi: 10.1371/journal.pone.0134526 (PMC4533973; doi:10.1371/journal.pone.0134526)
Supplement: S2 Fig — (DOCX) [file pone.0134526.s002.docx]

**S2 Fig. Protections against chemical probes in nucleotides of the central loop of domain V of 23S rRNA, caused by binding of CAM or CAM dimers (compounds 4 and 5) to *E. coli* ribosomes**. Ribosomes were incubated in the presence or absence of each compound at 25°C for 2 s or 3 min. The resulting complexes were then probed with DMS (panel A), CMCT (panel B) or Kethoxal (panel C). U, A, G and C, dideoxy sequencing lanes; lanes 1 and 5, unmodified ribosomes; lanes 2, 6, and 9, ribosomes probed in the absence of CAM dimers or CAM; lanes 3, 7, and 10, ribosomes pre-incubated with each CAM dimer or CAM for 2 s and then probed; lanes 4, 8, and 11, ribosomes pre-incubated with each CAM dimer or CAM for 3 min and then probed. Results obtained with CAM, although published previously [11], were repeated and are presented in lanes 9-11 for the sake of comparison. Numbering of nucleosides for the sequencing lanes is indicated at the left. Nucleosides with accessibility affected by bound CAM dimers are shown by arrows at the right, while reference bands whose intensity is not affected by CAM dimers or CAM binding are indicated by an asterisk.
