# Supplementary material for: Synthesis and Evaluation of Chloramphenicol Homodimers: Molecular Target, Antimicrobial Activity, and Toxicity against Human Cells
Source: PLoS One. 2015 Aug 12;10(8):e0134526. doi: 10.1371/journal.pone.0134526 (PMC4533973; doi:10.1371/journal.pone.0134526)
Supplement: S3 Fig — (DOCX) [file pone.0134526.s003.docx]

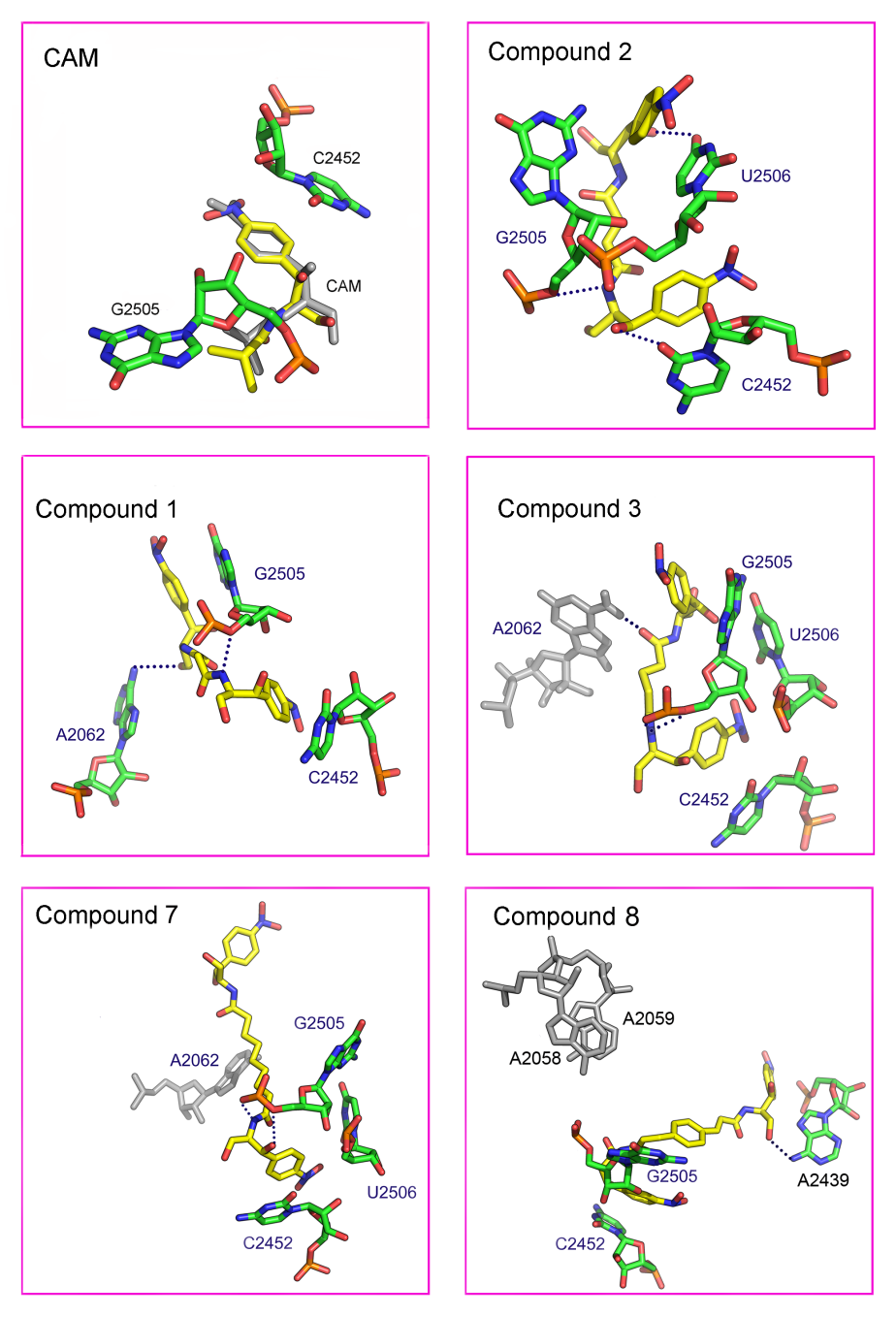


**S3 Fig. Binding positions of CAM dimers in the *E. coli* ribosome, as detected by Molecular Dynamics Simulations.** CAM: Overlay of CAM structures from MD simulation (yellow) and crystallographic analysis (grey; PDB:3OFC). Other residues of the 23S rRNA placed adjacently to the binding pocket of CAM**,** except for C2452 and G2505**,** are ignored for clarity. Compounds 1-3, 7, and 8: These dimers deviate from the classical binding site of CAM and do not reach the entrance to the exit tunnel. However, they make additional hydrogen bonds with nucleosides surrounding the catalytic center of PTase.
