# Supplementary material for: Synthesis and Evaluation of Chloramphenicol Homodimers: Molecular Target, Antimicrobial Activity, and Toxicity against Human Cells
Source: PLoS One. 2015 Aug 12;10(8):e0134526. doi: 10.1371/journal.pone.0134526 (PMC4533973; doi:10.1371/journal.pone.0134526)
Supplement: S4 Fig — (DOCX) [file pone.0134526.s004.docx]

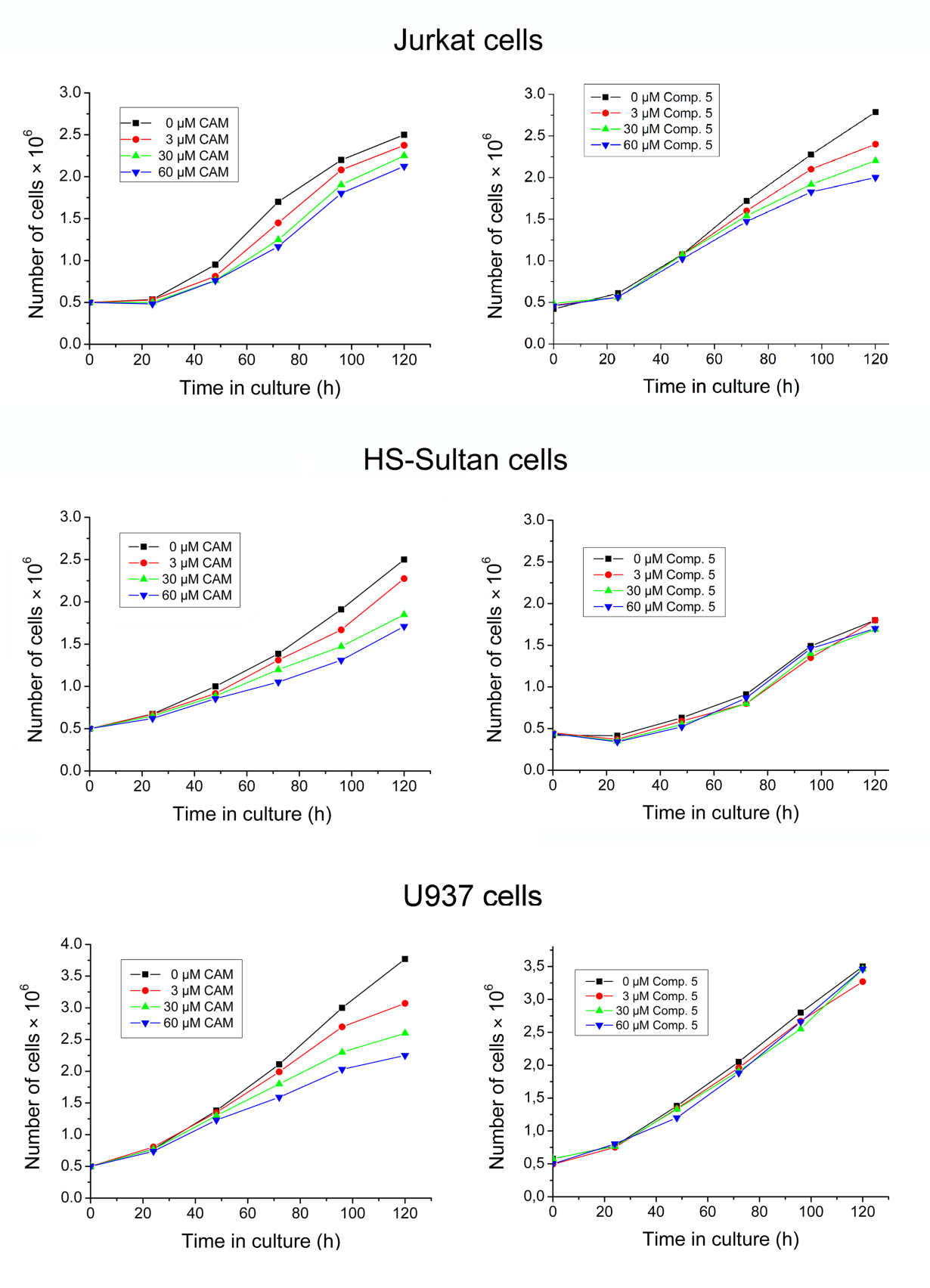


**S4 Fig.** **Toxicity assays in Human leukemic cell lines.** HS-Sultan, Jurkat, and U937 cell lines were adjusted to 5×10^5^ cells/L in RPMI-1640 medium containing 1% Penicillin/Streptomycin and 10% fetal bovine serum. Cells were grown in triplicate in the presence or absence of CAM or compound **5** at the indicated concentrations for 5 days at 37°C, under a humidified 5% CO_2_ atmosphere. Aliquots were collected daily and counted in a CELL-DYN 3700 Hematology Analyzer.
