# Supplementary material for: Synthesis and Evaluation of Chloramphenicol Homodimers: Molecular Target, Antimicrobial Activity, and Toxicity against Human Cells
Source: PLoS One. 2015 Aug 12;10(8):e0134526. doi: 10.1371/journal.pone.0134526 (PMC4533973; doi:10.1371/journal.pone.0134526)
Supplement: S1 Supplemental Procedures — Synthesis of 1,4-phenylenediacrylic acid, 1H- and 13C-NMR spectra and RP-HPLC chromatograms of CAM dimers 1–8. General procedure for the synthesis of compounds 9 and 10. Synthesis of compound 11. 1H- and 13C-NMR spectra of compounds 9–11. Supplemental references. (DOCX) [file pone.0134526.s005.docx]

# S1 Supplemental Procedures

1. **General Experimental Procedure for the Synthesis of Compounds 1, 2, 4 and 6-8.** To an ice-cold solution of CAM base (CLB) (0.21 g, 1.0 mmol), the appropriate dicarboxylic acid (0.5 mmol) in DMF (1.0 mL), HBTU (0.52 g, 1.3 mmol) and ^i^Pr_2_NEt (0.44 mL, 2.5 mmol) were added. The reaction mixture was stirred at ambient temperature until consumption of reactants. The reaction mixture was then diluted with EtOAc, and the organic phase was washed once with a 5% aqueous NaHCO_3_ solution, twice with brine, dried over Na_2_SO_4_ and finally evaporated to dryness. The CAM dimers obtained were purified by FCC.

**1.1. *N*^1^,*N*^3^-bis((1*R*,2*R*)-1,3-dihydroxy-1-(4-nitrophenyl)propan-2-yl)malonamide (1).** Reaction time: 1h; Yield: 0.18 g (75%); White solid; mp.:142-143 ^o^C; R*_f_* (CHCl_3_/MeOH 8:2):0.22; IR (KBr, cm^-1^): 3374, 3282, 1655, 1636, 1516, 1348, 1082, 842; MS (ESI, 30eV): *m/z* 531.29 [M+K], 515.30 [M+Na], 493.38 [M+H]; ^1^H NMR (*d_6_*-DMSO): *δ* 8.12 (d, *J* = 8.8 Hz, 4H), 7.87 (d, *J* = 9.2 Hz, 2H), 7.54 (d, *J* = 8.8 Hz, 4H), 5.88 (d, *J* = 4.4 Hz, 2H), 4.99 (br.s, 2H), 4.85 (unresolved dd, 2H), 3.97-3.87 (m, 2H), 3.54-3.44 (m, 2H), 3.28-3.22 (m, 2H), 2.92 (s, 2H); ^13^C NMR (*d_6_*-DMSO): *δ* 167.3 (two C), 152.2 (two C), 146.8 (two C), 127.8 (four C), 123.3 (four C), 69.4 (two C), 60.7 (two C), 56.7 (two C), 46.2.

**1.2. *N*^1^,*N*^4^-bis((1*R*,2*R*)-1,3-dihydroxy-1-(4-nitrophenyl)propan-2-yl)fumaramide (2).** Reaction time: 1h; Yield: 0.14 g (55%); White solid; mp.:207-208 ^o^C; R*_f_* (CHCl_3_/MeOH 9:1):0.15; IR (KBr, cm^-1^): 3522, 3412, 3296, 3274, 1632, 1616, 1544, 1514, 1350, 1048, 858; MS (ESI, 30eV): *m/z* 543.14 [M+K], 527.26 [M+Na], 505.35 [M+H]; ^1^H NMR (*d_6_*-DMSO, 60^o^C): *δ* 8.14 (d, *J* = 8.8 Hz, 4H), 7.92 (d, *J* = 9.2 Hz, 2H) 7.58 (d, *J* = 8.8 Hz, 4H), 6.73 (s, 2H), 5.68 (d, *J* = 4.4 Hz, 2H), 5.03 (br.s, 2H), 4.72-4.65 (m, 2H), 4.12-4.04 (m, 2H), 3.63-3.55 (m, 2H), 3.37-3.30 (m, 2H); ^13^C NMR (*d_6_*-DMSO): *δ* 164.2 (two C), 152.3 (two C), 146.8 (two C), 133.2 (two C), 127.8 (four C), 123.3 (four C), 69.9 (two C), 60.9 (two C), 56.8 (two C).

**1.3. *N*^1^,*N*^6^-bis((1*R*,2*R*)-1,3-dihydroxy-1-(4-nitrophenyl)propan-2-yl)adipamide (4).** Reaction time: 1h; Yield: 0.23 g (85%); White foam; R*_f_* (CHCl_3_/MeOH 8:2): 0.25; IR (KBr, cm^-1^): 3402, 1652, 1646, 1636, 1552, 1348, 1072, 848; MS (ESI, 30eV): *m/z* 573.24 [M+K], 557.31 [M+Na], 535.33 [M+H]; ^1^H NMR (*d_6_*-DMSO): *δ* 8.14 (d, *J* = 8.8 Hz, 4H), 7.56 (d, *J* = 8.8 Hz, 4H), 7.42 (dd, *J* = 1.6 and 8.8 Hz, 2H), 5.83 (dd, *J* = 1.8 and 4.8 Hz, 2H), 5.01 (dd, *J* = 1.8 and 4.4 Hz, 2H), 4.87-4.82 (m, 2H), 4.02-3.93 (m, 2H), 3.66-3.58 (m, 2H), 3.56-3.50 (m, 2H), 3.32-3.24 (dt, *J* = 4.8 and 10.4 Hz, 2H), 3.17-3.09 (m, 2H), 1.93-1.84 (m, 4H); ^13^C NMR (*d_6_*-DMSO): *δ* 172.4 (two C), 152.5 (two C), 146.7 (two C), 127.7 (four C), 123.2 (four C), 69.7 (two C), 60.9 (two C), 56.2 (two C), 35.0 (two C), 24.9 (two C).

**1.4. *N*^1^,*N*^8^-bis((1*R*,2*R*)-1,3-dihydroxy-1-(4-nitrophenyl)propan-2-yl)octane-diamide (6).** Reaction time: 1h; Yield: 0.19 g (67%); White solid; mp.:131-132 ^o^C; R*_f_* (CHCl_3_/MeOH 9:1): 0.11; IR (KBr, cm^-1^): 3484, 3322, 3275, 2930, 1659, 1642, 1531, 1428, 1348, 1048, 846; MS (ESI, 30eV): *m/z* 585.27 [M+Na]; ^1^H NMR (*d_6_*-DMSO): *δ* 8.15 (d, *J* = 8.8 Hz, 4H), 7.57 (d, *J* = 8.8 Hz, 4H), 7.44 (d, *J* = 8.8 Hz, 2H), 5.82 (d, *J* = 4.8 Hz, 2H), 5.02 (dd, *J* = 2.4 and 4.0 Hz, 2H), 4.84 (dd, *J* = 4.8 and 6.0 Hz, 2H), 4.03-3.95 (m, 2H), 3.57-3.49 (m, 2H), 3.31-3.25 (m, 2H), 1.92 (t, *J* = 6.8 Hz, 4H), 1.20-1.11 (m, 4H), 0.90-0.79 (m, 4H); ^13^C NMR (*d_6_*-DMSO): *δ* 172.5 (two C), 152.6 (two C), 146.7 (two C), 127.8 (four C), 123.2 (four C), 69.7 (two C), 61.0 (two C), 56.1 (two C), 35.4 (two C), 28.6 (two C), 25.5 (two C).

**1.5. *N*^1^,*N*^9^-bis((1*R*,2*R*)-1,3-dihydroxy-1-(4-nitrophenyl)propan-2-yl)nonane-diamide (7).** Reaction time: 1h; Yield: 0.26 g (89%); White solid; mp.:137-139 ^o^C; R*_f_* (CHCl_3_/MeOH 8:2): 0.32; IR (KBr, cm^-1^): 3410, 2932, 1642, 1518, 1348, 848; MS (ESI, 30eV): *m/z* 1175.45 [2M+Na], 599.55 [M+Na], 577.57 [M+H]; ^1^H NMR (*d_6_*-DMSO): *δ* 8.15 (d, *J* = 8.8 Hz, 4H), 7.58 (d, *J* = 8.8 Hz, 4H), 7.42 (d, *J* = 9.2 Hz, 2H), 5.80 (d, *J* = 4.8 Hz, 2H), 5.03 (unresolved dd, 2H), 4.82 (unresolved dd, 2H), 4.04-3.96 (m, 2H), 3.59-3.50 (m, 2H), 3.33-3.25 (m, 2H), 1.99-1.89 (m, 4H), 1.25-1.15 (m, 4H), 1.03-0.94 (m, 2H), 0.92-0.79 (m, 4H); ^13^C NMR (*d_6_*-DMSO): *δ* 172.5 (two C), 152.6 (two C), 146.7 (two C), 127.8 (four C), 123.2 (four C), 69.8 (two C), 61.0 (two C), 56.1 (two C), 35.5 (two C), 29.0, 28.7 (two C), 25.7 (two C).

**1.6. *N*,*N^’^*-bis((1*R*,2*R*)-1,3-dihydroxy-1-(4-nitrophenyl)propan-2-yl)-1,4-phenylenediacryl- diamide (8).** Reaction time: 1h; Yield: 0.25 g (83%); White solid; mp.:144-145 ^o^C; R*_f_* (CHCl_3_/MeOH 9:1): 0.18; IR (KBr, cm^-1^): 3435, 3338, 2938, 1660, 1616, 1522, 1348, 1220, 1058, 844; MS (ESI, 30eV): *m/z* 629.36 [M+Na], 607.31 [M+H]; ^1^H NMR (*d_6_*-DMSO, 50 ^o^C): *δ* 8.15 (d, *J* = 8.8 Hz, 4H), 7.70 (d, *J* = 8.8 Hz, 2H), 7.63 (d, *J* = 8.8 Hz, 4H), 7.52 (s, 4H), 7.25 (d, *J* = 16 Hz, 2H), 6.81 (d, *J* = 16 Hz, 2H), 5.89-5.74 (m, 2H), 5.09 (s, 2H), 4.87-4.70 (m, 2H), 4.19-4.10 (m, 2H), 3.62 (unresolved dd, 2H), 3.40 (dd, *J* = 5.6 and 10 Hz, 2H); ^13^C NMR (*d_6_*-DMSO): *δ* 165.5 (two C), 152.2 (two C), 146.8 (two C), 138.6 (two C), 136.2 (two C), 128.5 (four C), 127.8 (four C), 123.3 (four C), 119.0 (two C), 69.9 (two C), 61.0 (two C), 56.7 (two C).

**2. Synthesis of *N*^1^,*N*^5^-bis((1*R*,2*R*)-1,3-dihydroxy-1-(4-nitrophenyl)propan-2yl)- glutaramide (3).** To an ice-cold solution of CLB (0.21 g, 1.0 mmol) in DMF (2.5 mL), glutaric anhydride (0.13 g, 1.1 mmol) was added. The reaction mixture was stirred at ambient temperature for 2h, and then cooled at 0 ^o^C HBTU (0.44 g, 1.1 mmol) and ^i^Pr_2_NEt (0.38 mL, 2.2 mmol) were sequentially added. After 1h at ambient temperature, the reaction mixture was diluted with EtOAc and then washed once with a 5% aqueous NaHCO_3_ solution and twice with brine. The organic phase was dried over Na_2_SO_4_ and evaporated to dryness. Compound **3** was purified by FCC. Yield: 0.42 g (80%); White foam; R*_f_* (CHCl_3_/MeOH 85:15): 0.18; IR (KBr, cm^-1^): 3332, 2926, 1644, 1520, 1348, 1074, 848; MS (ESI, 30eV): *m/z* 559.34 [M+K], 543.20 [M+Na], 521.28 [M+H]; ^1^H NMR (*d_6_*-DMSO): *δ* 8.14 (d, *J* = 8.4 Hz, 4H), 7.56 (d, *J* = 8.4 Hz, 4H), 7.41 (d, *J* = 9.2 Hz, 2H), 5.79 (d, *J* = 4.8 Hz, 2H), 5.00 (dd, *J* = 2.4 and 4.8 Hz, 2H), 4.82 (unresolved dd, 2H), 4.00-3.92 (m, 2H), 3.57-3.49 (m, 2H), 3.31-3.24 (dt *J* = 4.8 and 10.4 Hz, 2H), 1.86-1.79 (m, 4H), 1.36 (quintet, *J* = 7.2 Hz, 2H); ^13^C NMR (*d_6_*-DMSO): *δ* 172.2 (two C), 152.5 (two C), 146.8 (two C), 127.8 (four C), 123.2 (four C), 70.0 (two C), 60.9 (two C), 56.2 (two C) 35.0 (two C), 18.5.

**3. Synthesis of *N*^1^,*N*^4^-bis((1*R*,2*R*)-1,3-dihydroxy-1-(4-nitrophenyl)propan-2-yl)- terephthalamide (5).** To an ice-cold solution of CLB (0.21 g, 1.0 mmol), Et_3_N (0.17 mL, 1.2 mmol) in DMF (1.5 mL) and terephthaloyl chloride (0.091 g, 0.45 mmol) were added. The reaction mixture was stirred at ambient temperature for 2 h and then diluted with EtOAc. The organic phase was washed once with a 5% aqueous NaHCO_3_ solution, twice with brine, dried over Na_2_SO_4_, evaporated to a minimum volume and finally refrigerated overnight. The precipitate was filtered, washed twice with Et_2_O and dried under vacuo. Yield: 0.2 g (80%); White solid; mp.:239-240 ^o^C; R*_f_* (CHCl_3_/MeOH 9:1): 0.15; IR (KBr, cm^-1^): 3552, 3387, 3316, 2930, 1636, 1516, 1350, 1048, 860; MS (ESI, 30eV): *m/z* 577.51 [M+Na], 555.53 [M+H]; ^1^H NMR (*d_6_*-DMSO, 60^o^C): *δ* 8.13 (d, *J* = 8.4 Hz, 4H), 7.81 (d, *J* = 7.6 Hz, 2H), 7.75 (s, 4H), 7.62 (d, *J* = 8.4 Hz, 4H), 5.72 (d, *J* = 4.4 Hz, 2H), 5.13-5.07 (m, 2H), 4.77-4.66 (m, 2H), 4.32-4.23 (m, 2H), 3.70 (unresolved dt, 2H), 3.47 (unresolved dt, 2H); ^13^C NMR (*d_6_*-DMSO): *δ* 166.2 (two C), 152.3 (two C), 146.8 (two C), 137.1 (two C), 127.9 (four C), 127.6 (four C), 123.3 (four C), 70.5 (two C), 60.8 (two C), 57.3 (two C).

**4. Synthesis of 1,4-phenylenediacrylic acid**

1,4-Phenylenediacrylic acid was synthesized for the needs of the present work, using a Doebner modification of the Knoevenagel reaction, as follows:

To a solution of terephthaldehyde (1.34 g, 10 mmol) in pyridine (15 mL), malonic acid (3.77 g, 36 mmol) and piperidine (0.2 mL) were added. The reaction mixture was heated at 100^o^C for 30 min, while a precipitate was formed. Then, it was left to attain ambient temperature and it was poured into a small amount of water. The mixture was cooled at 0^o^C and a 5N HCl solution was added carefully to pH 7. The precipitate was filtered, washed twice with water, twice with acetone and dried overnight under vacuo.

Yield: 1.90 g (87%); White solid; IR (KBr, cm^-1^): 3400-2800, 1674, 1620, 1426, 1310, 944, 826; MS (ESI, 30eV): *m/z* 437.39 [2M+H]; ^1^H NMR (*d_6_*-DMSO): *δ* 12.46 (br.s, 2H), 7.73 (s, 4H), 7.60 (d, *J* = 16.0 Hz, 2H), 6.60 (d, *J* = 16.0 Hz, 2H); ^13^C NMR (*d_6_*-DMSO): *δ* 168.4 (two C), 144.0 (two C), 136.8 (two C), 129.6 (four C), 121.2 (two C).

**4.1. ^1^H- and ^13^C-NMR spectra of 1,4-phenylenediacrylic acid**

**
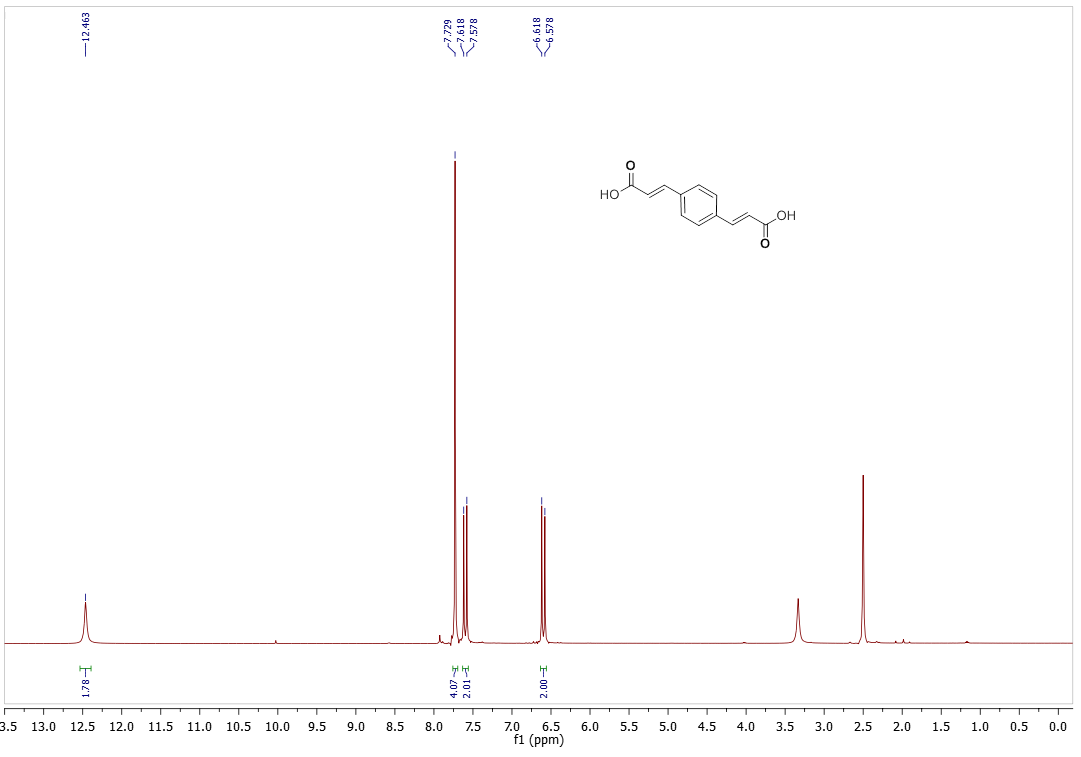
**

**
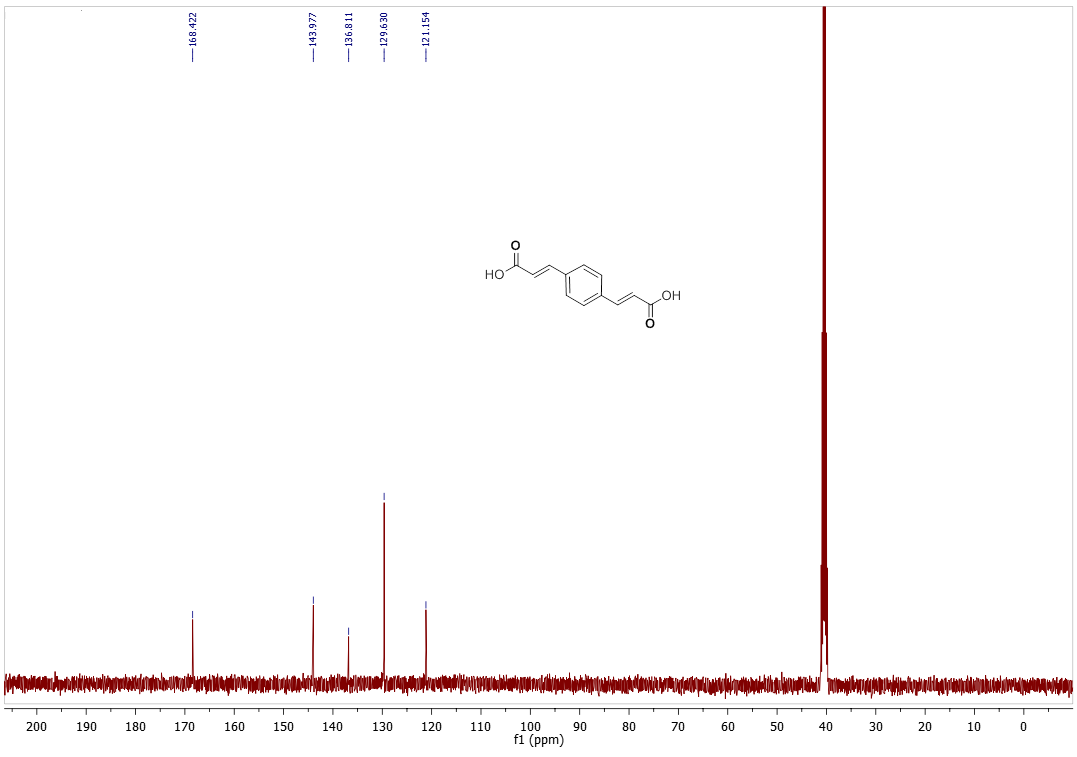
**

**5. ^1^H- and ^13^C-NMR spectra and RP-HPLC chromatograms of CAM dimers 1-8**

^1^H- and ^13^C-NMR spectra were processed using MestReNova software (Mestrelab Research), whereas HPLC chromatograms were processed using MassLynx v.4.0 software.

**5.1. *N^1^*,*N^3^*-Bis((*1R*,*2R*)-1,3-dihydroxy-1-(4-nitrophenyl)propan-2-yl)malonamide (1)**

**
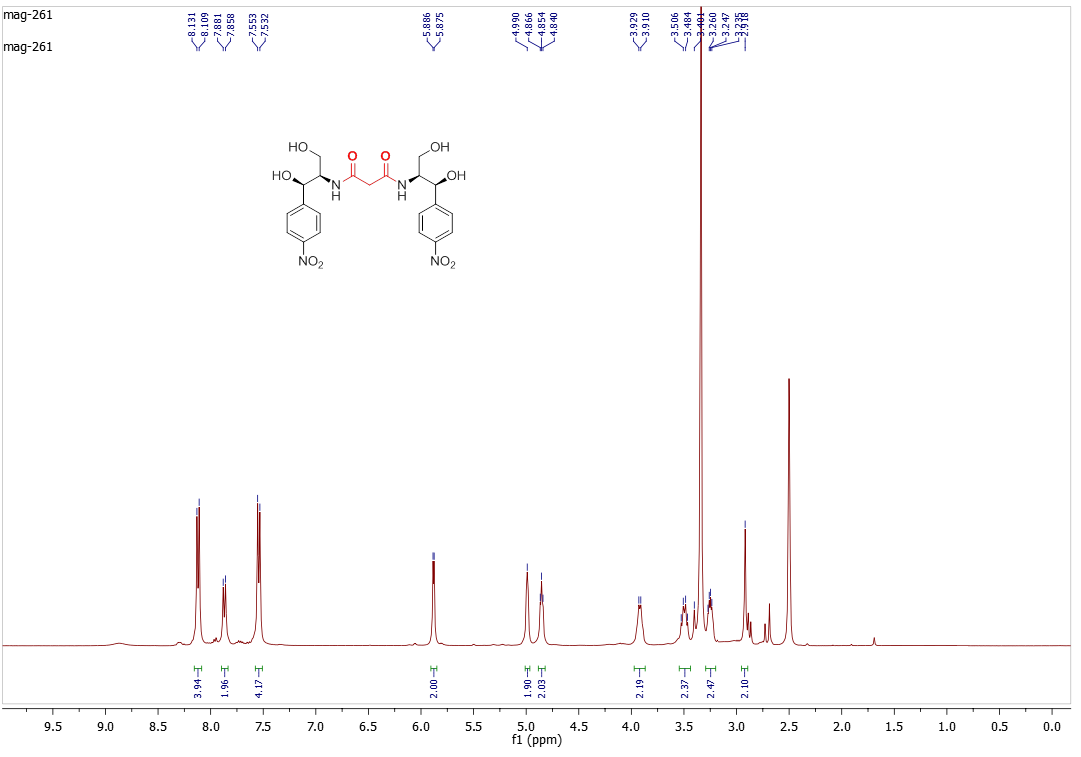
**

**
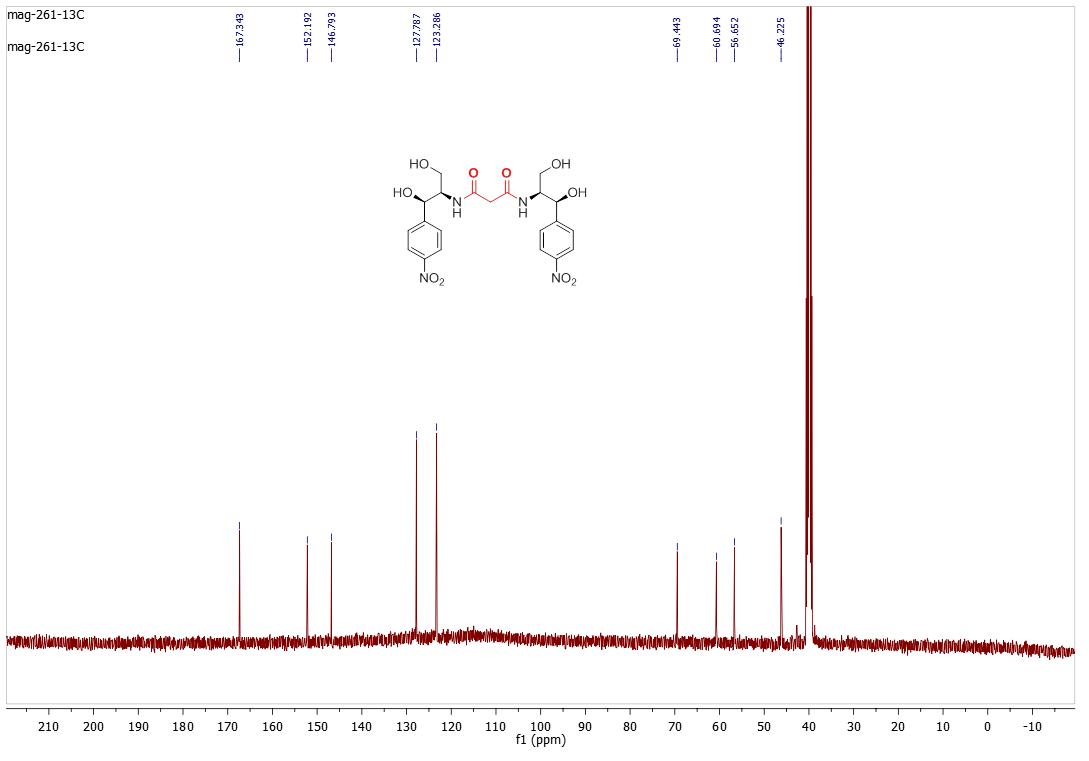
**

**
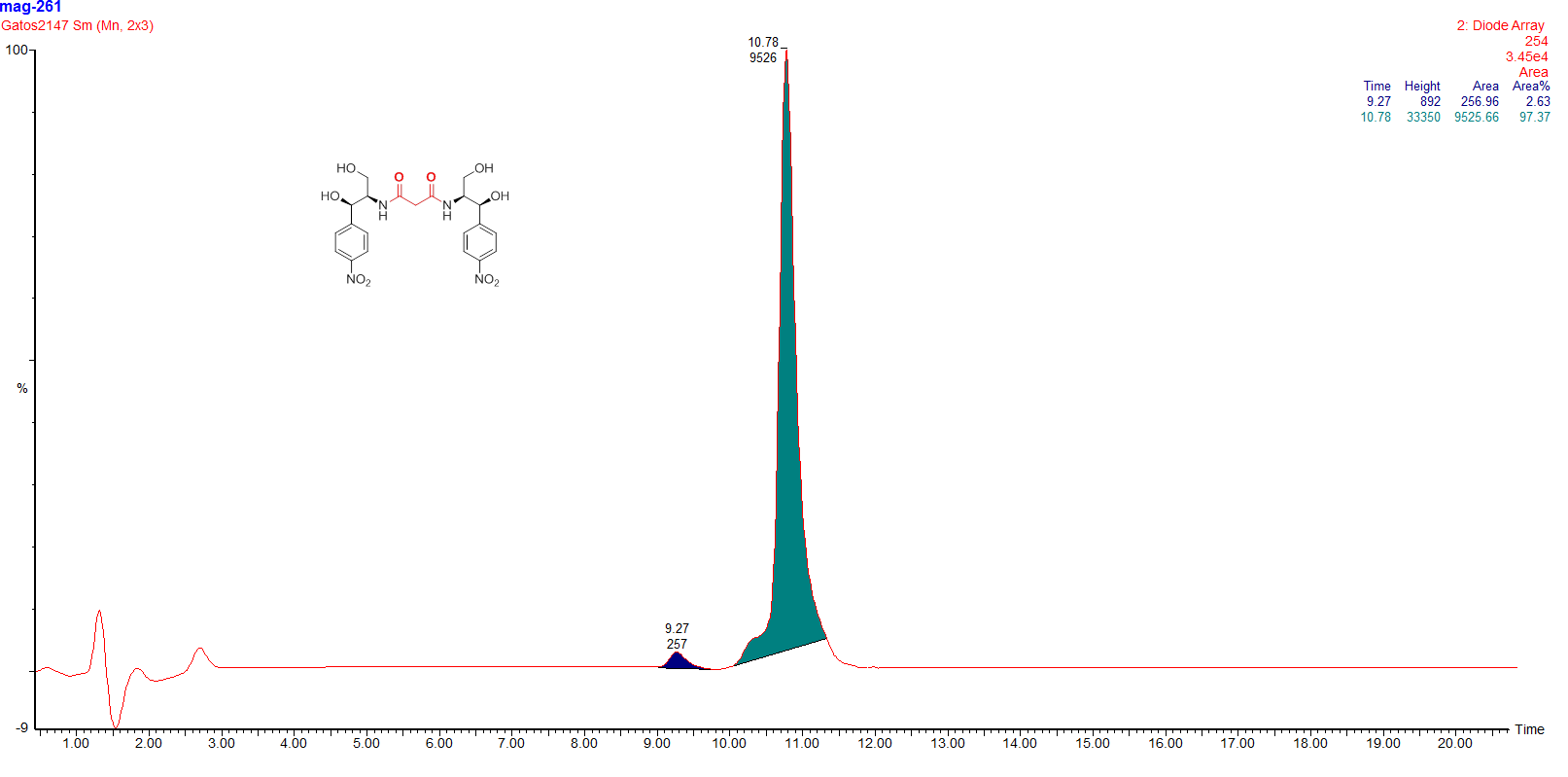
**

**5.2. *N^1^,N^4^*-Bis((*1R*,*2R*)-1,3-dihydroxy-1-(4-nitrophenyl)propan-2-yl)fumaramide (2)**

**
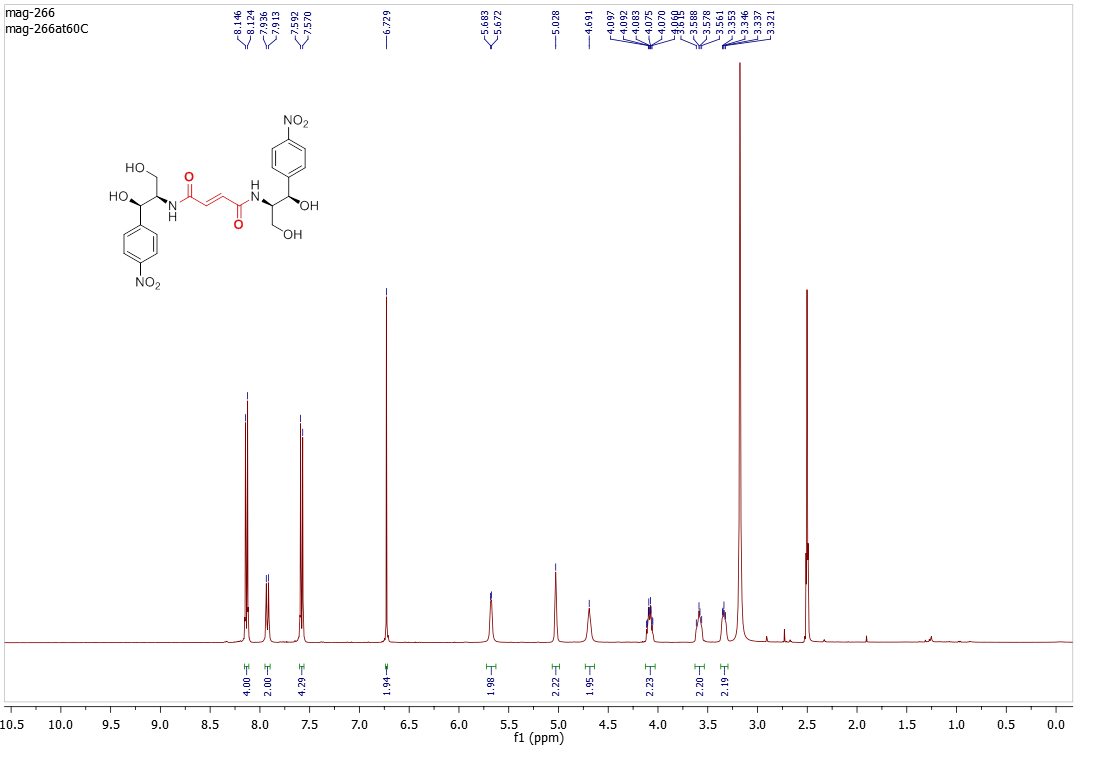
**

**
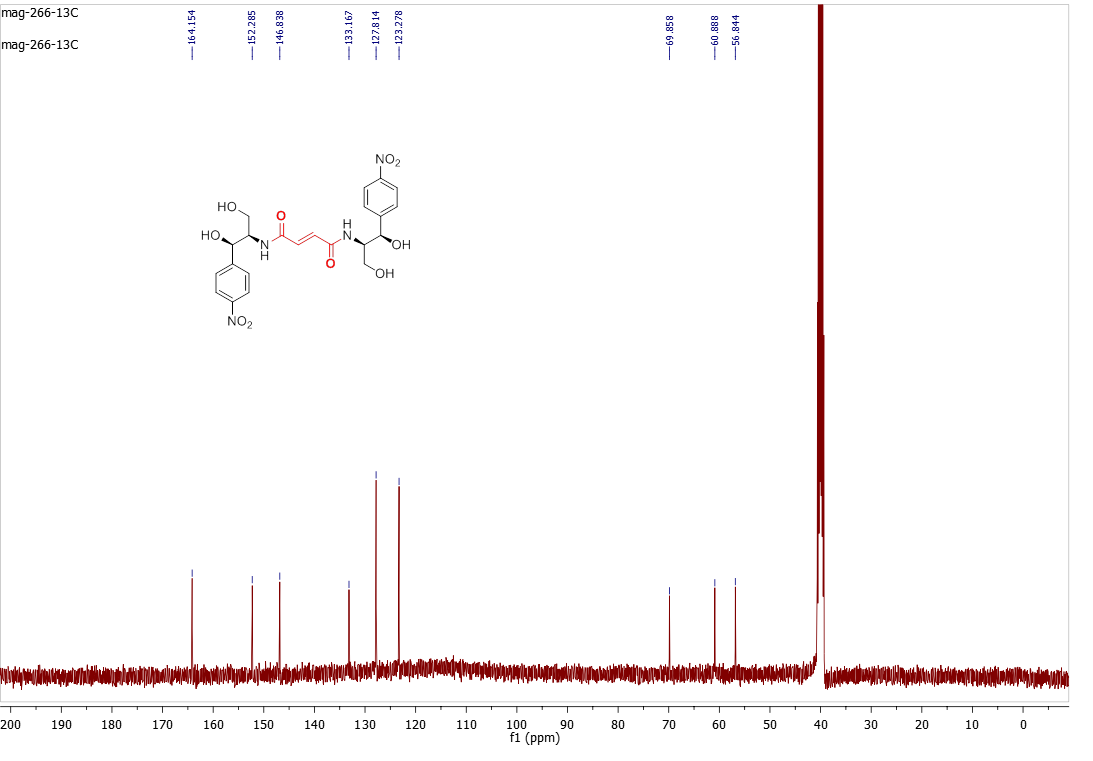
**

**
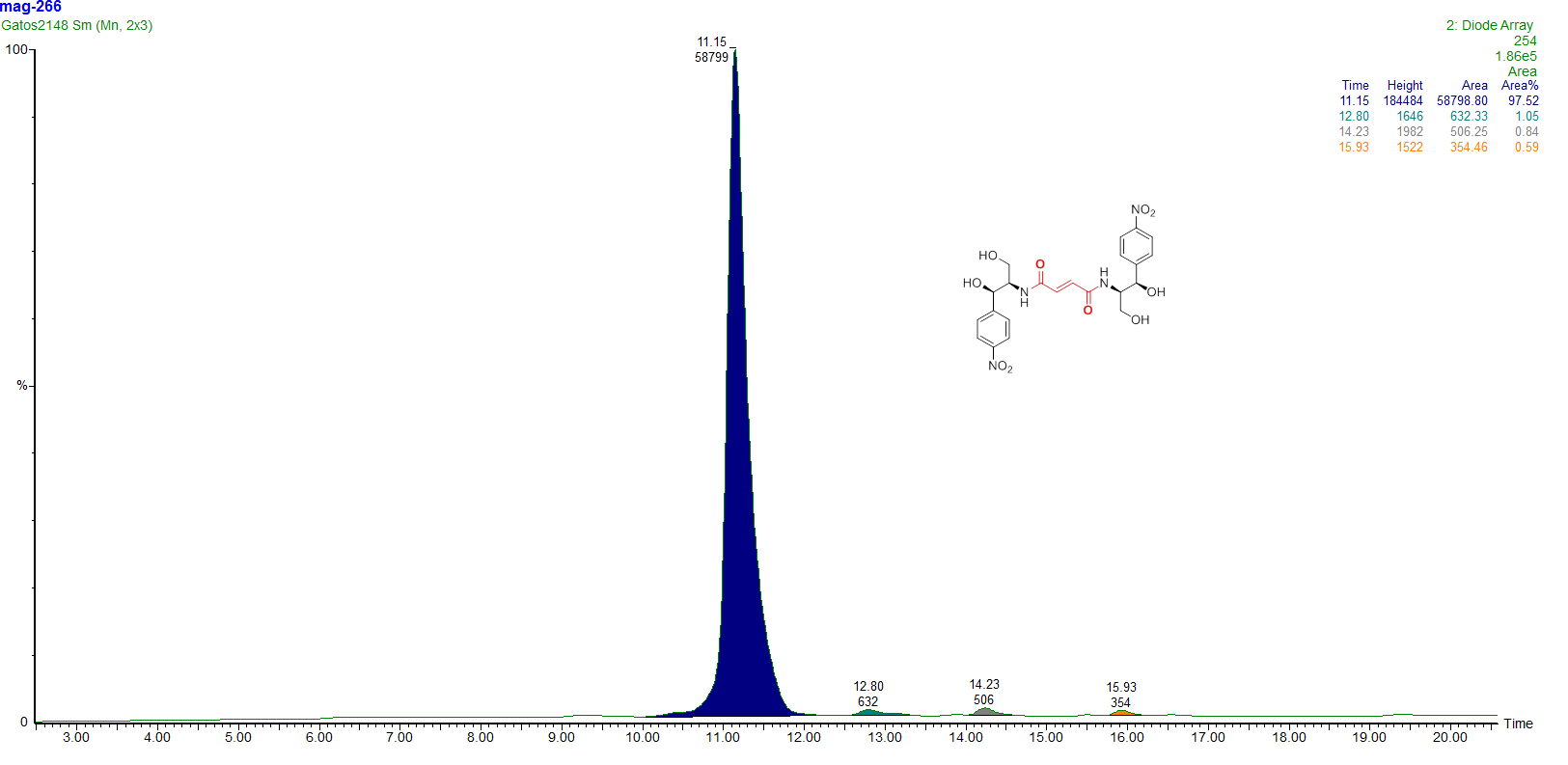
**

**5.3. *N^1^*,*N^5^*-Bis((*1R*,*2R*)-1,3-dihydroxy-1-(4-nitrophenyl)propan-2-yl)glutaramide (3)**

**
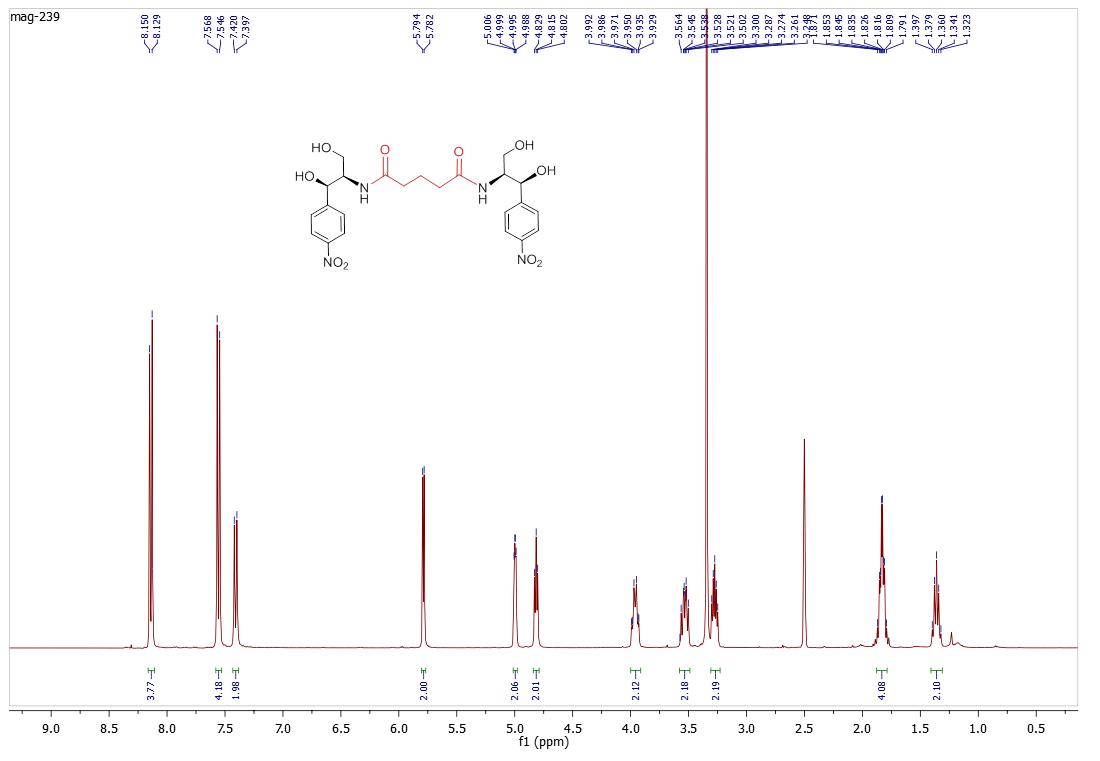
**

**
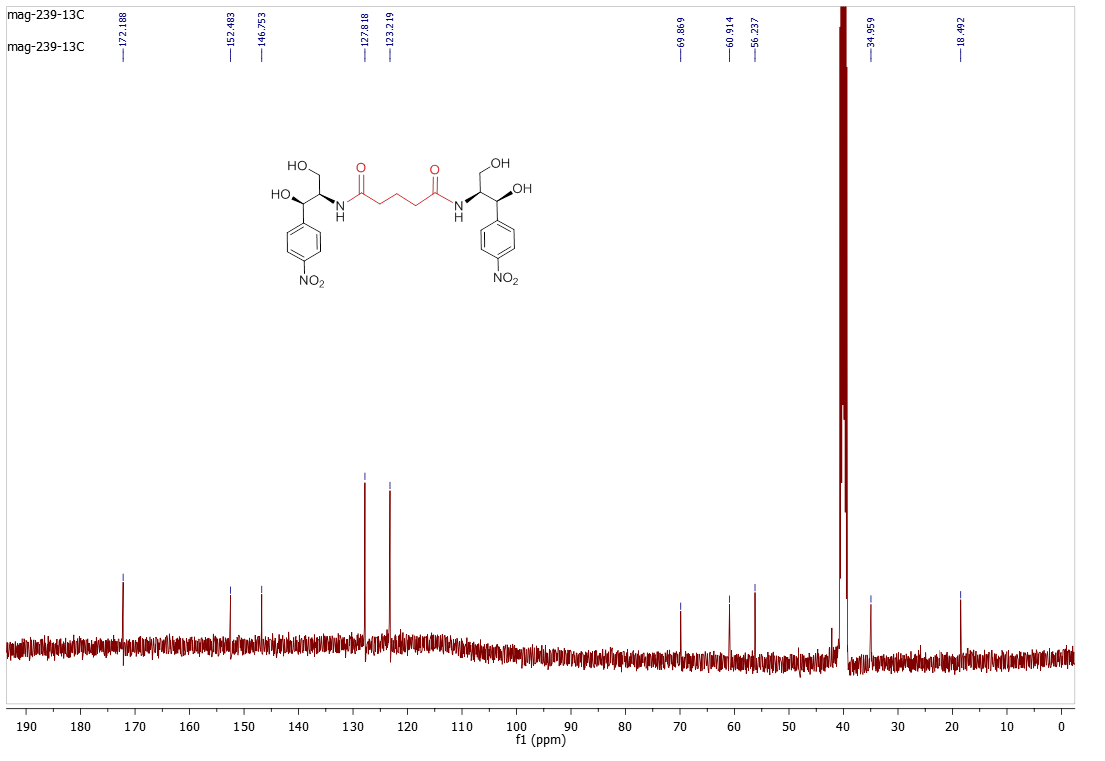
**

**
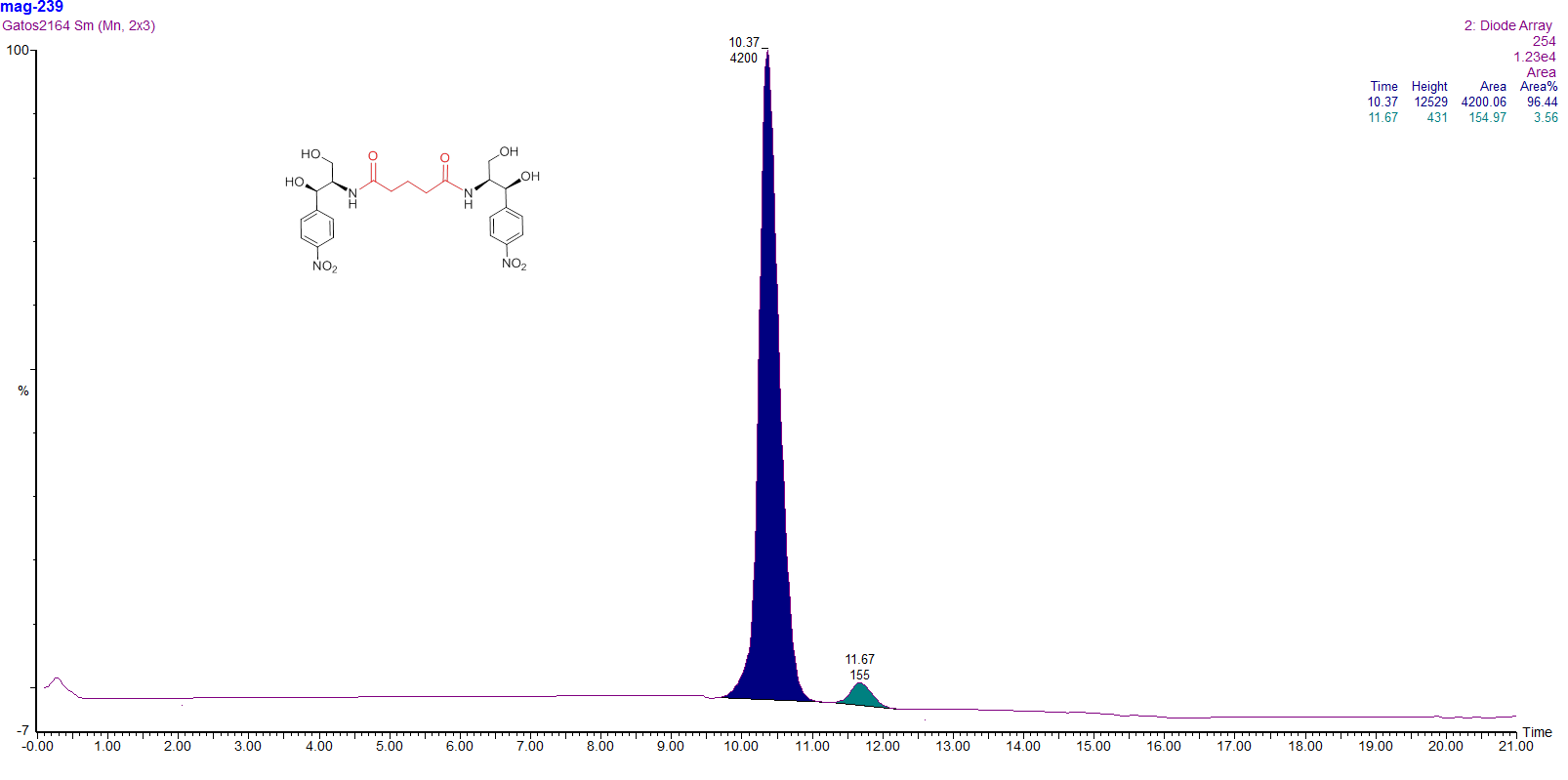
**

**5.4. *N^1^*,*N^6^*-Bis((*1R*,*2R*)-1,3-dihydroxy-1-(4-nitrophenyl)propan-2-yl)adipamide (4)**

**
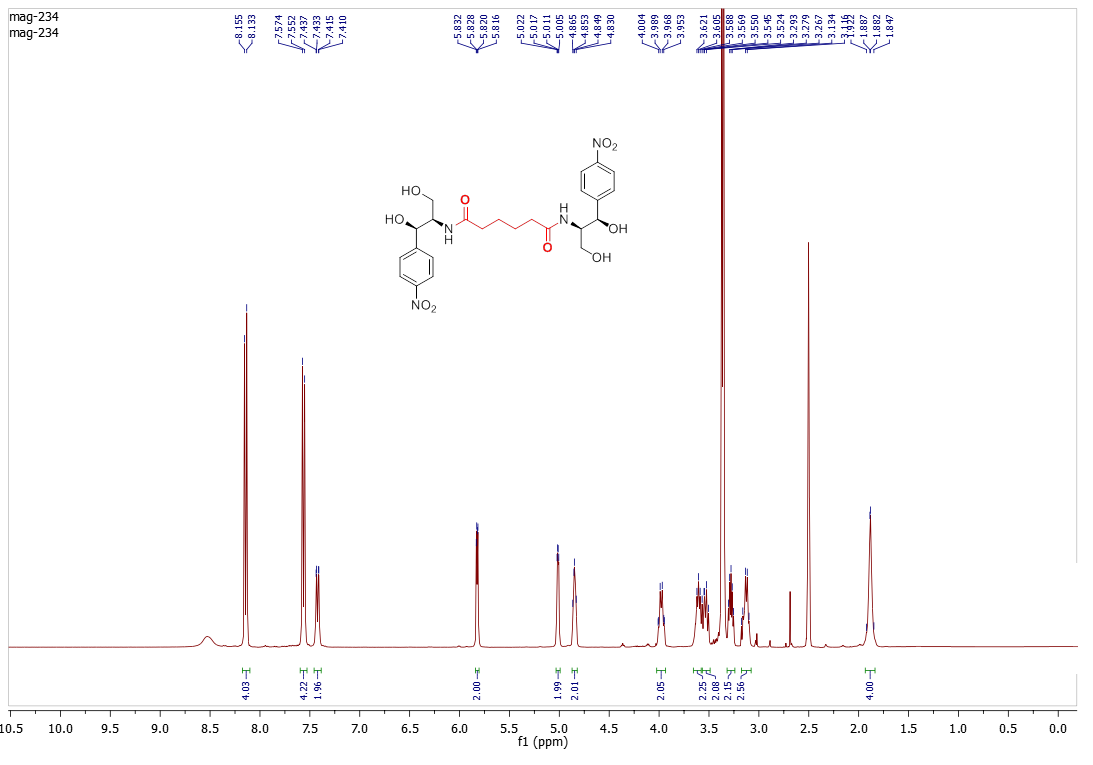
**

**
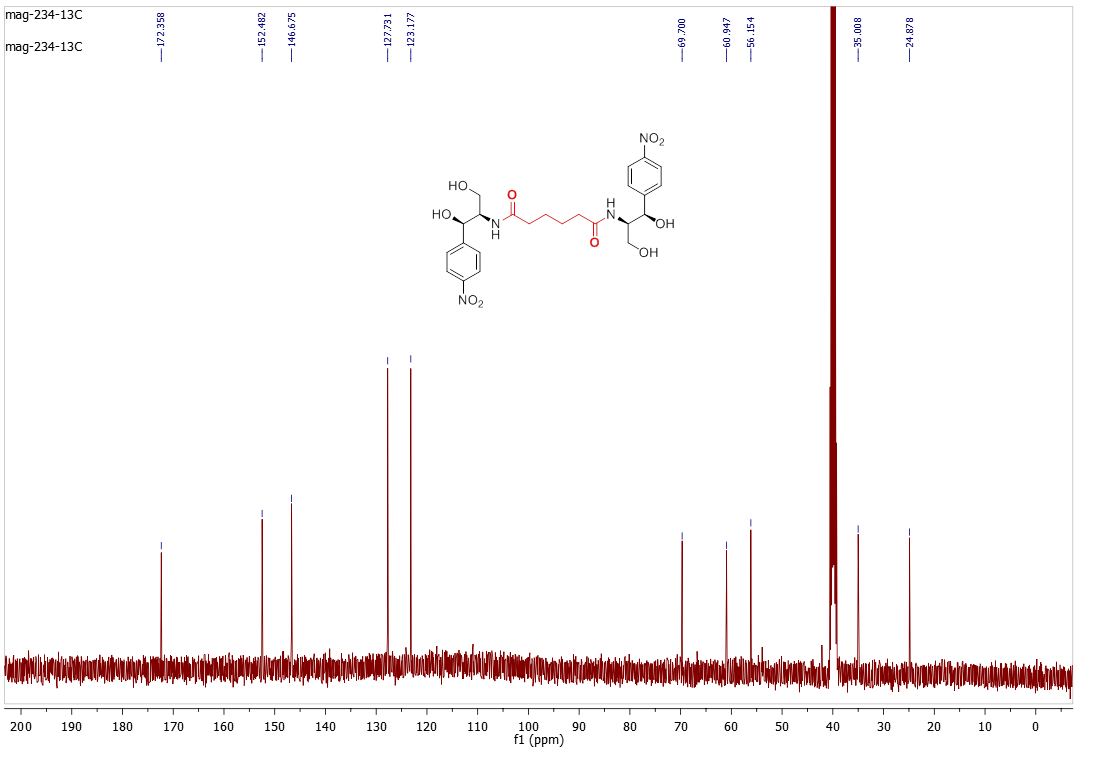
**

**
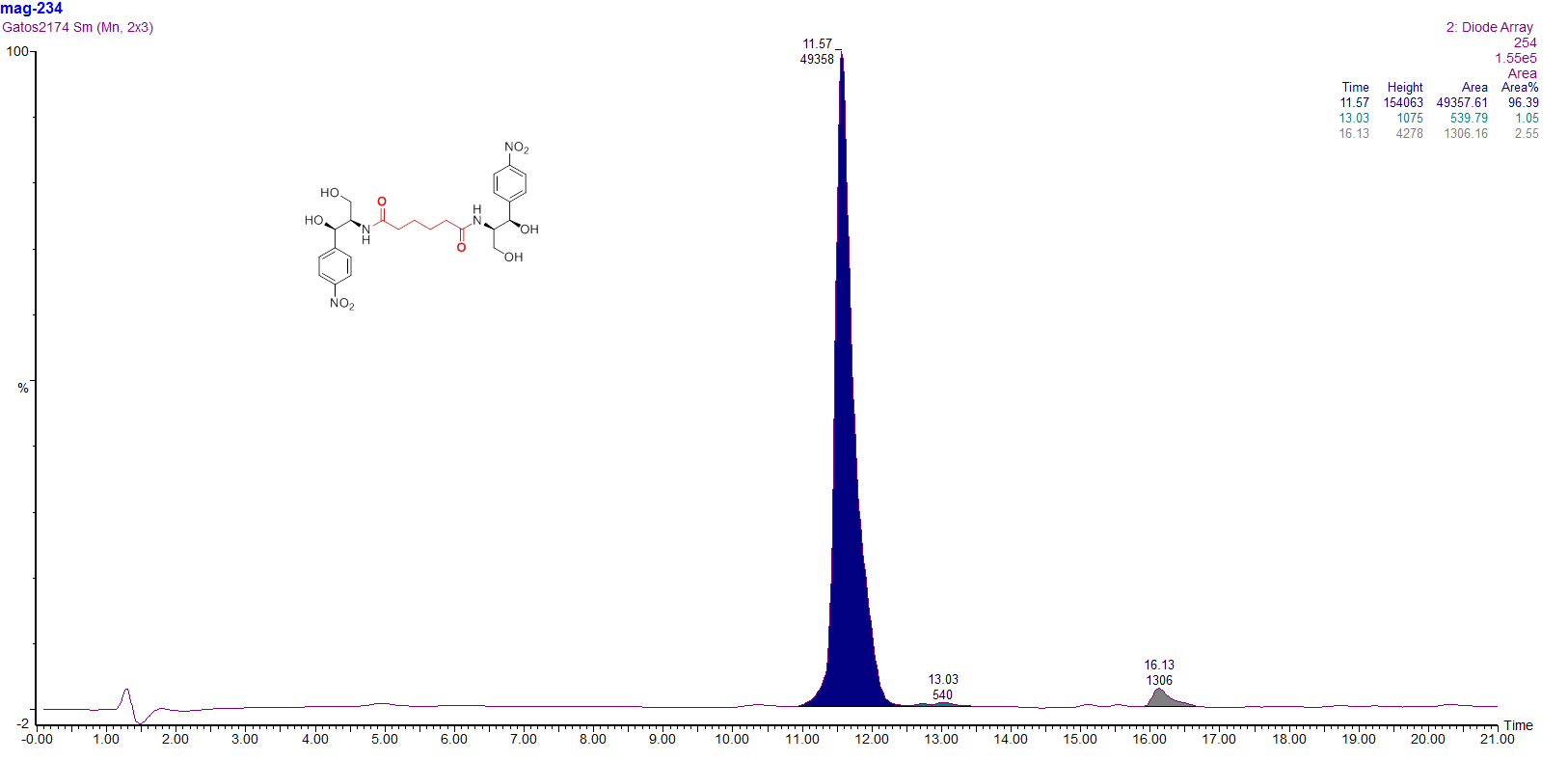
**

**5.5. *N^1^*,*N^4^*-Bis ((*1R*,*2R*)-1,3-dihydroxy-1-(4-nitrophenyl)propan-2-yl)-terephthalamide (5)**

**
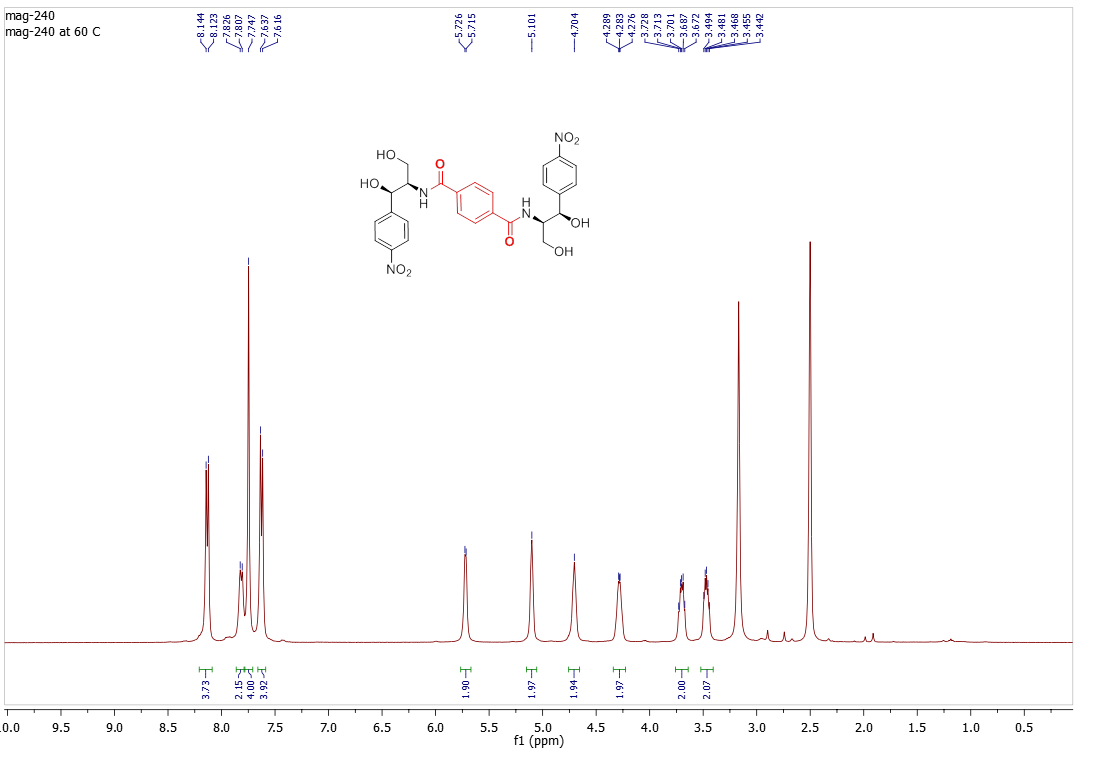
**

**
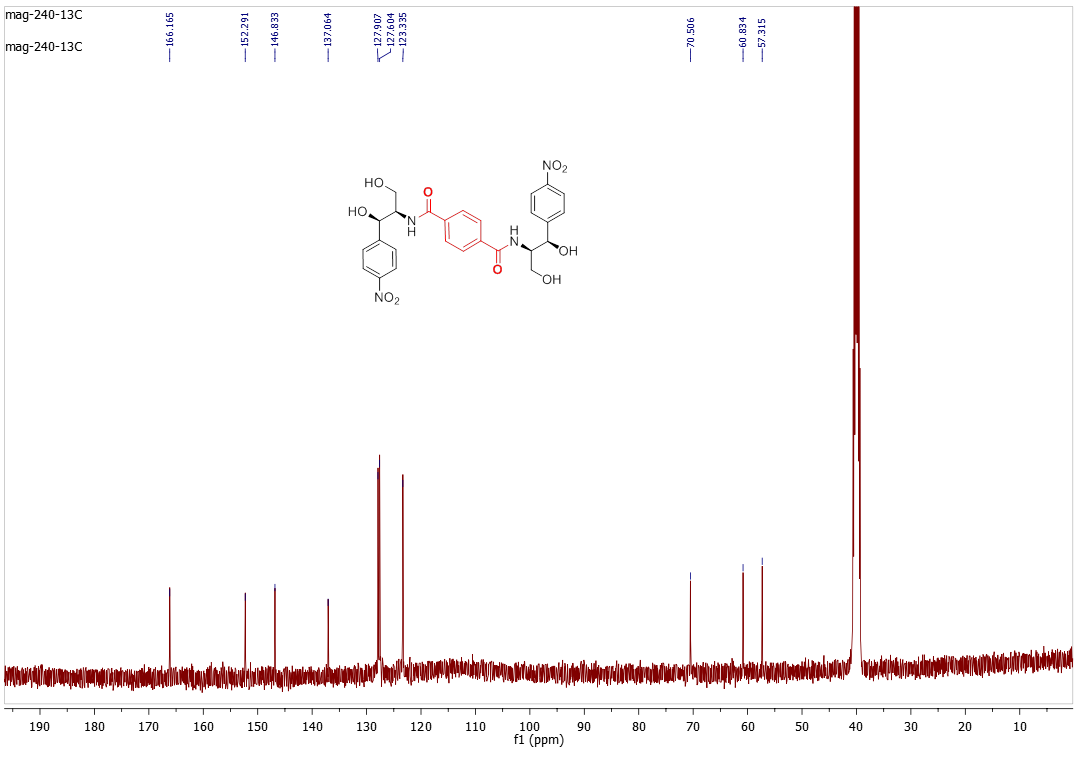
**

**
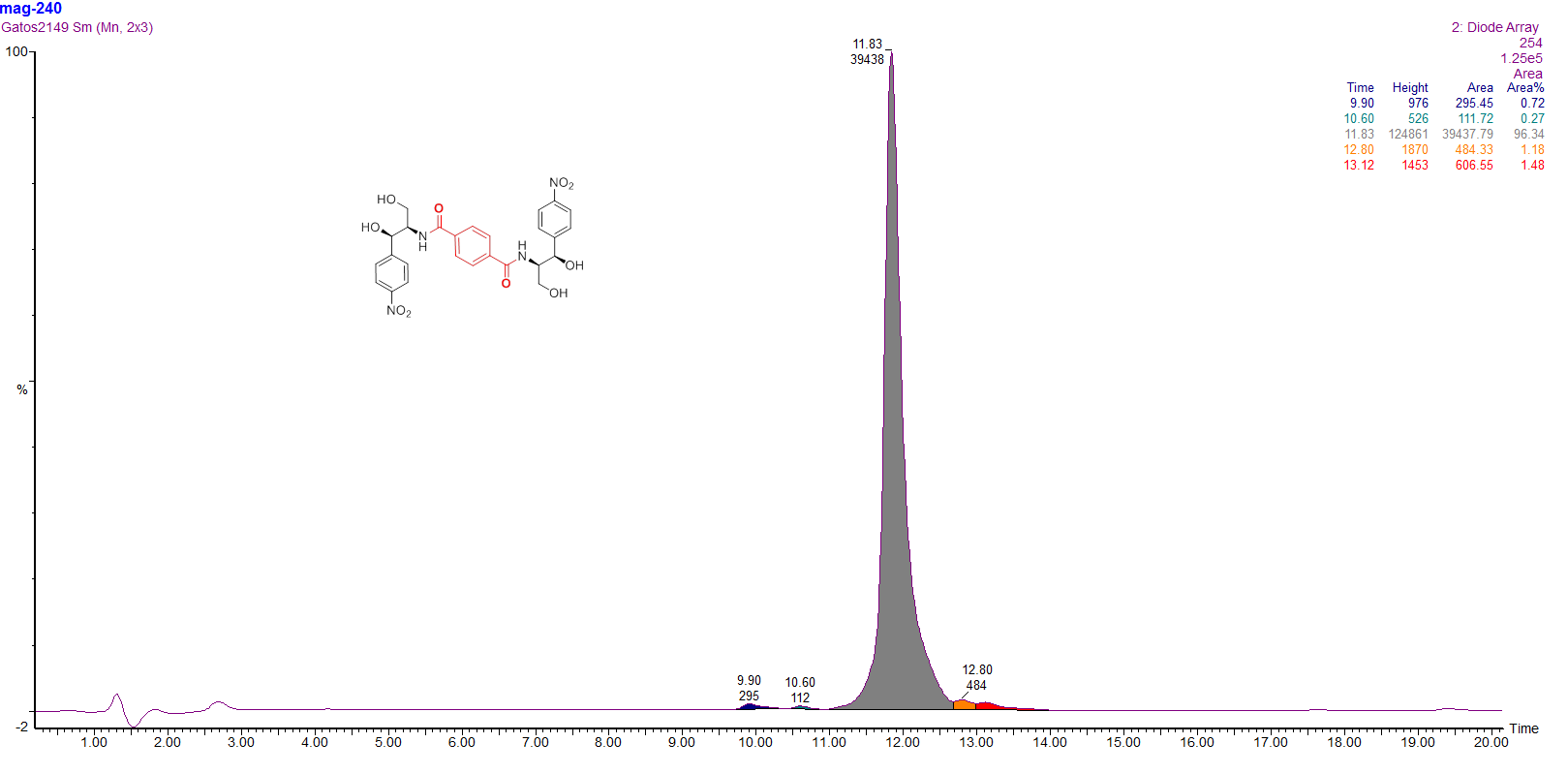
**

**5.6. *N^1^*,*N^8^*-Bis((*1R*,*2R*)-1,3-dihydroxy-1-(4-nitrophenyl)propan-2-yl)octane-diamide (6)**

**
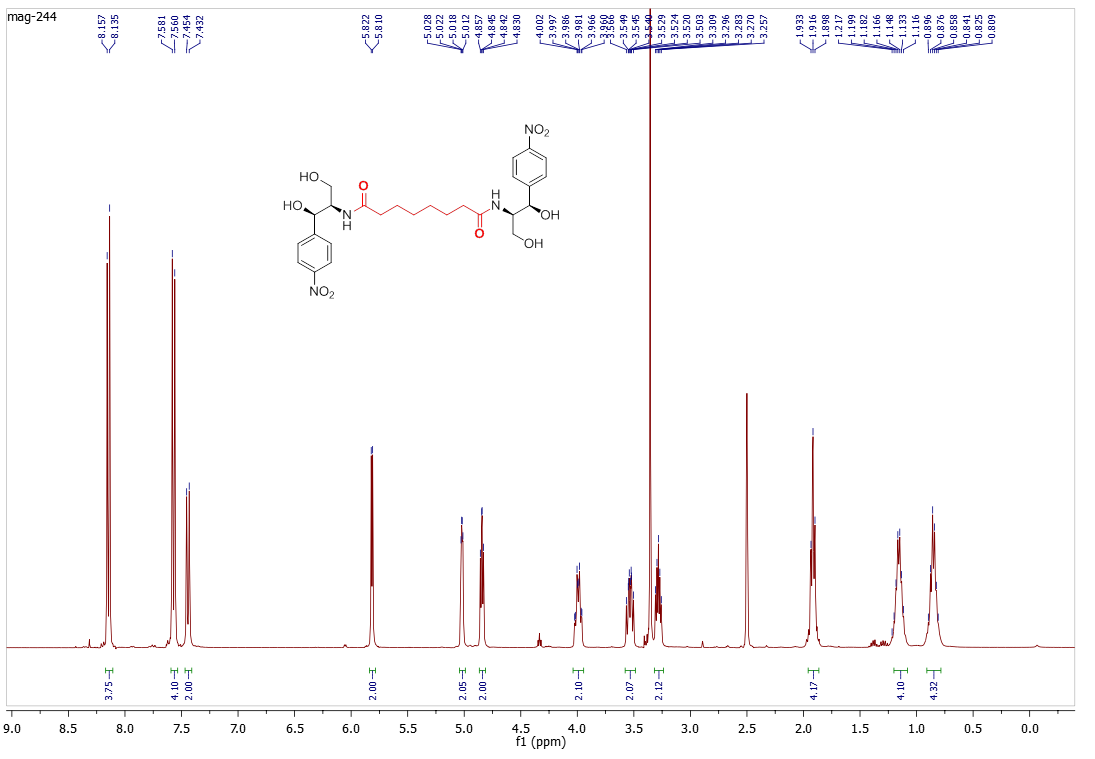
**

**
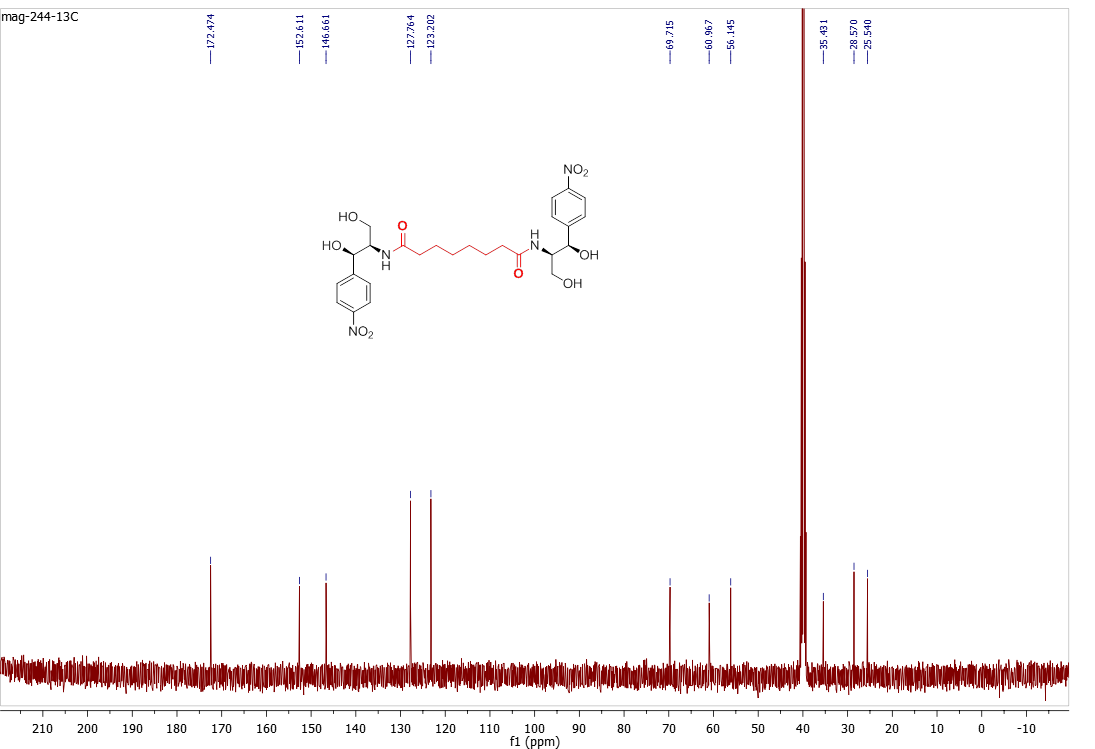
**

**
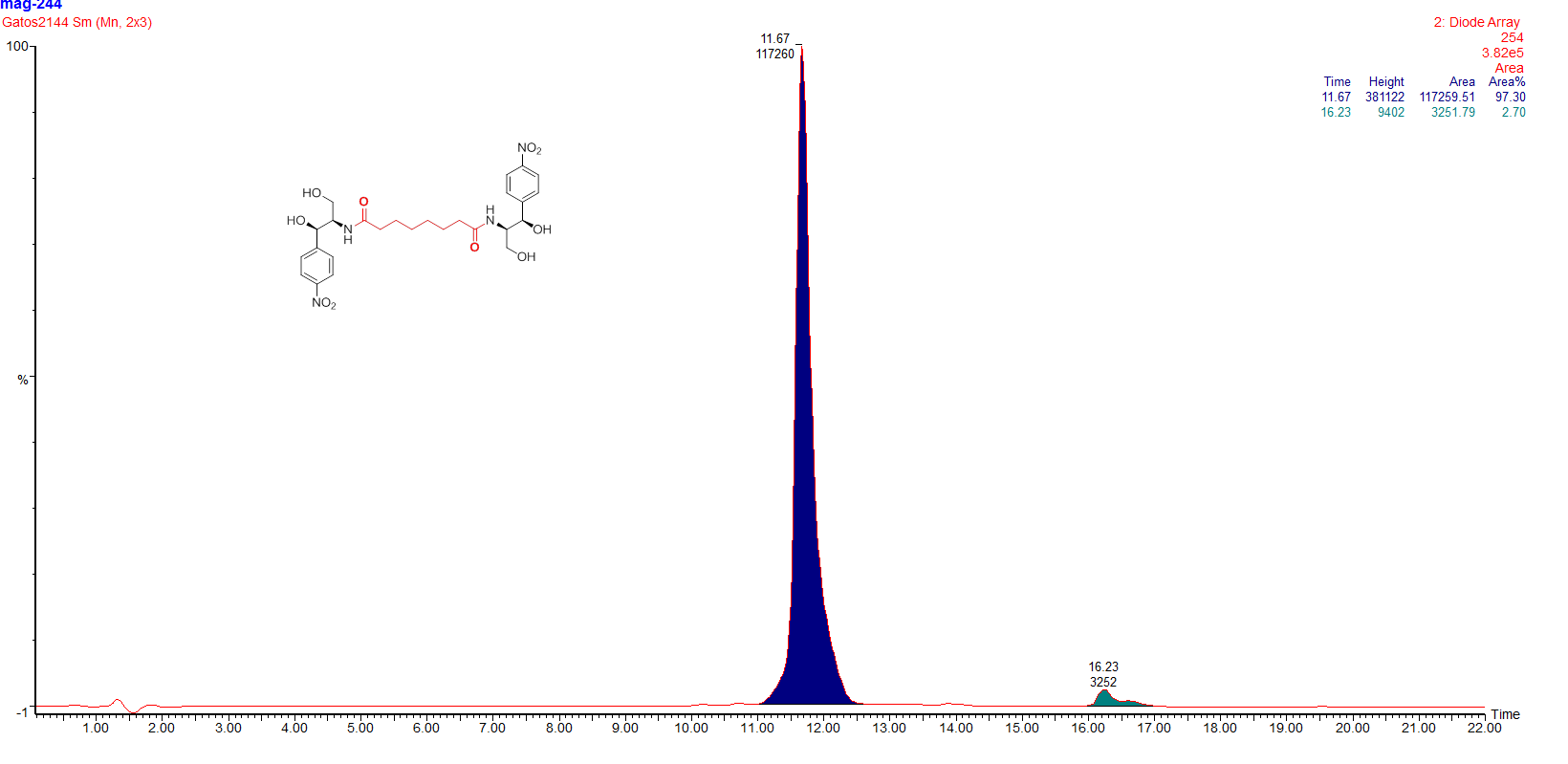
**

**5.7. *N^1^,N^9^*-Bis((*1R,2R*)-1,3-dihydroxy-1-(4-nitrophenyl)propan-2-yl)nonanediamide (7)**

**
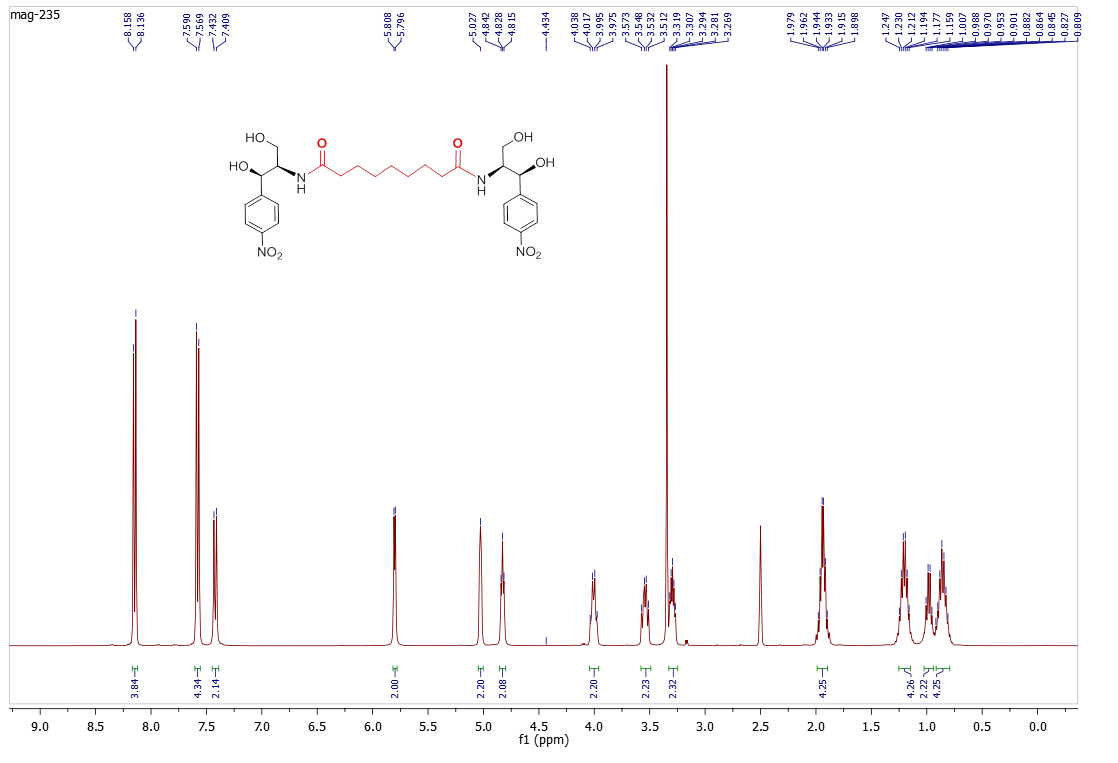
**

**
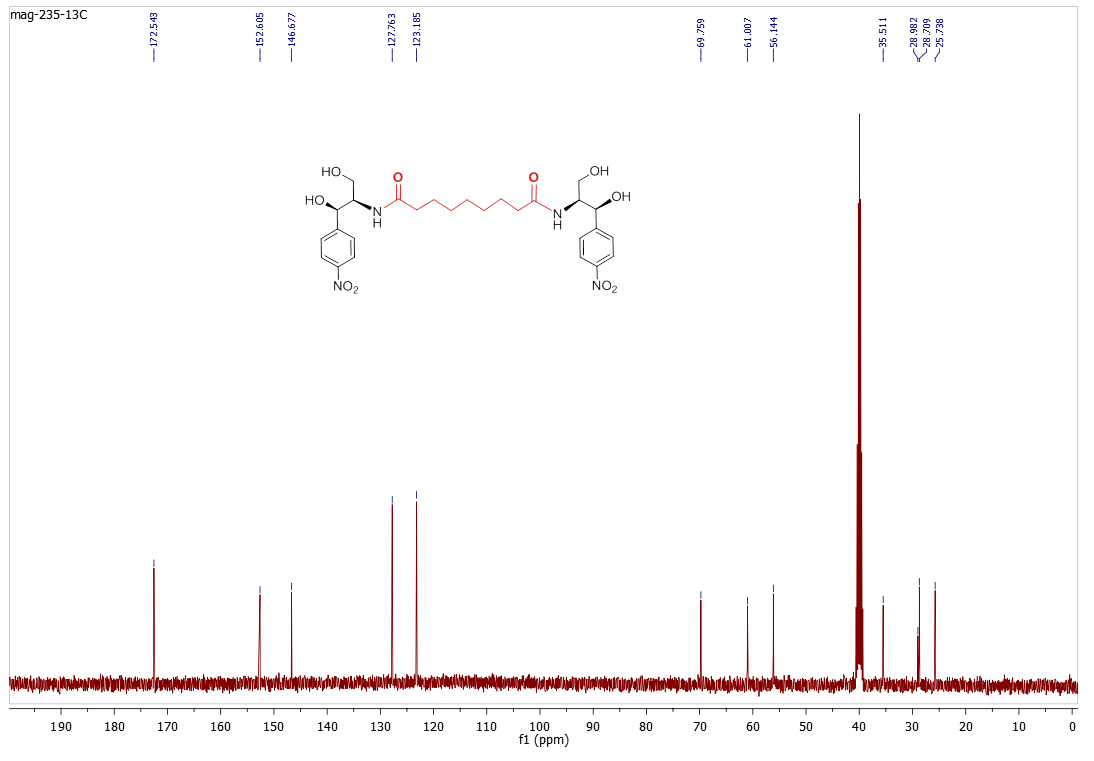
**

**
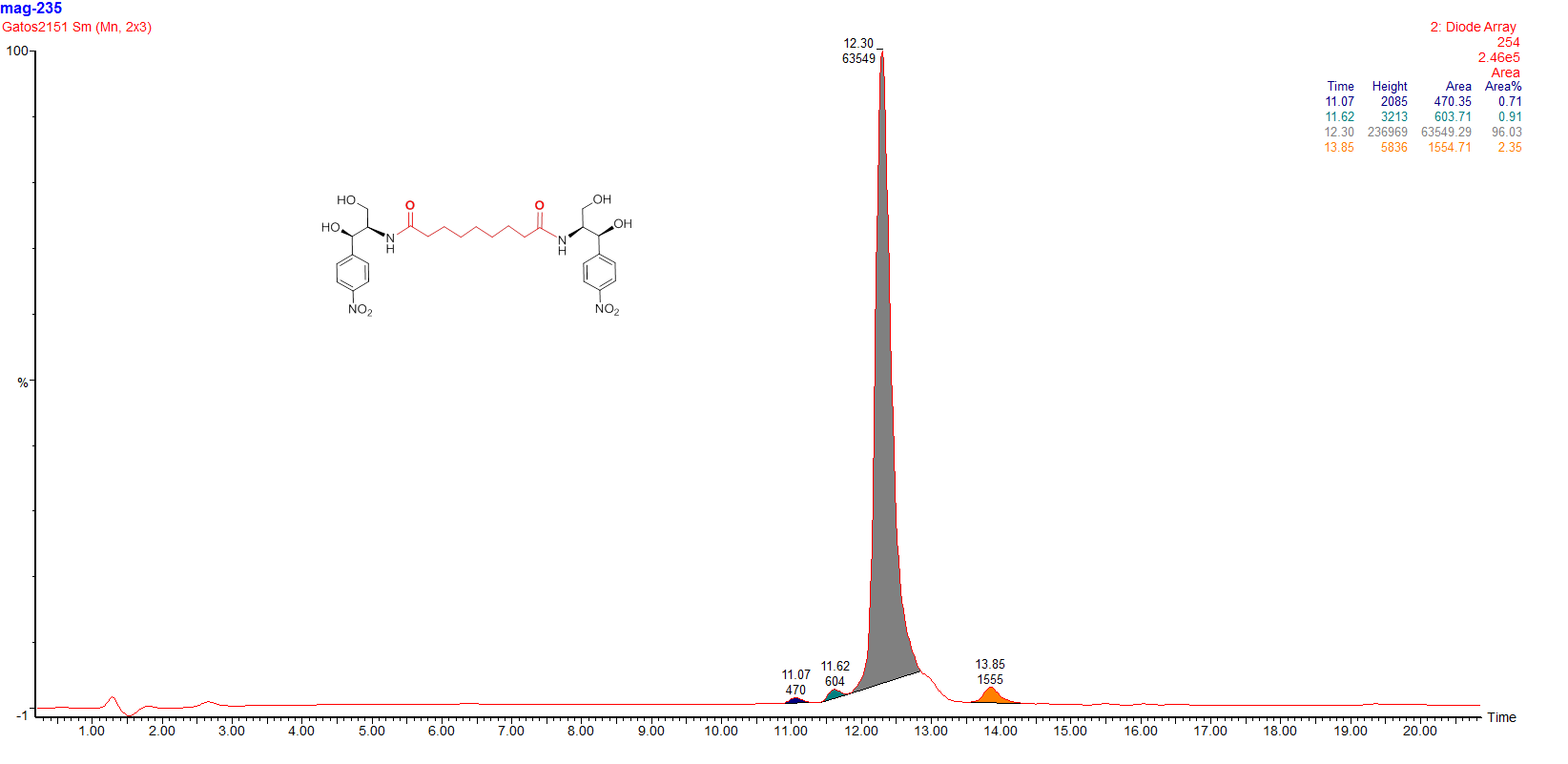
**

**5.8. *N,N^’^*-Bis((*1R,2R*)-1,3-dihydroxy-1-(4-nitrophenyl)propan-2-yl)-1,4-phenylene diacryldiamide (8)**

**
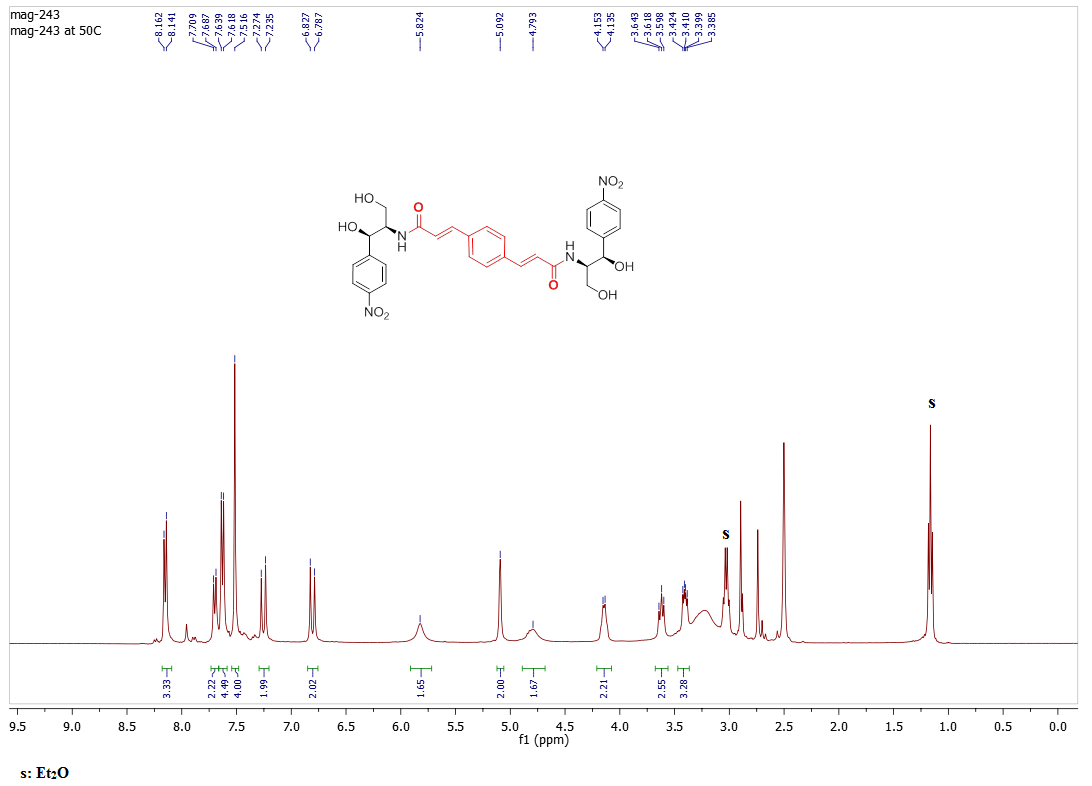
**

**
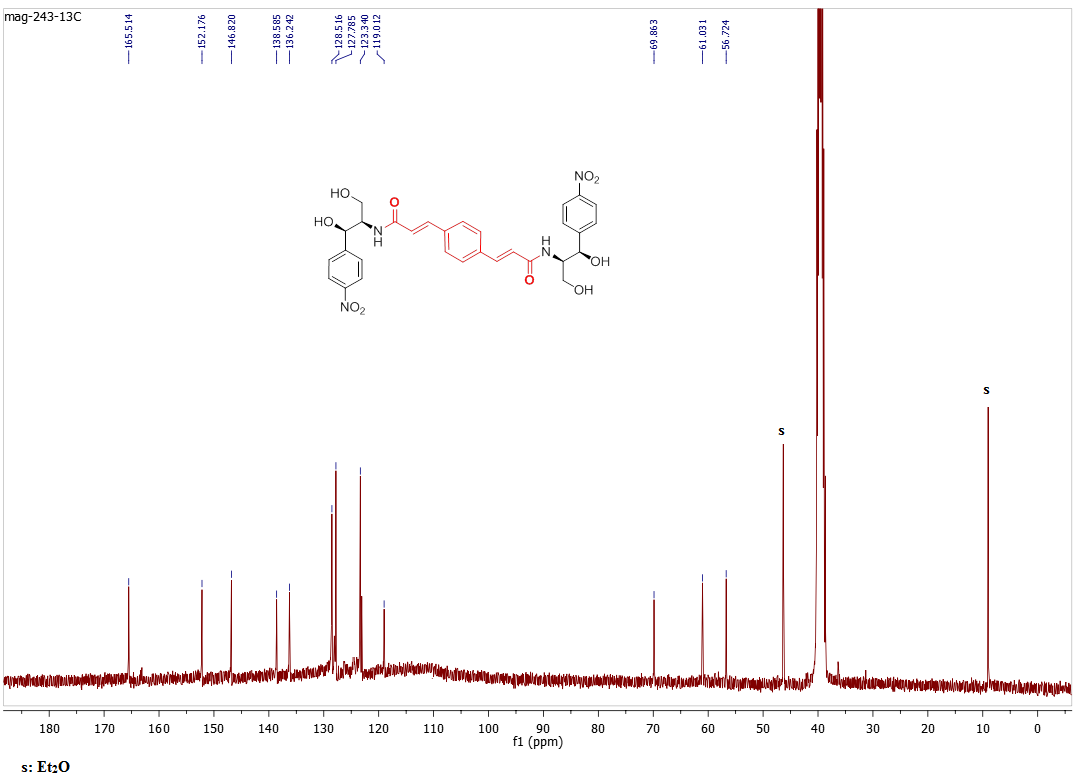
**

**
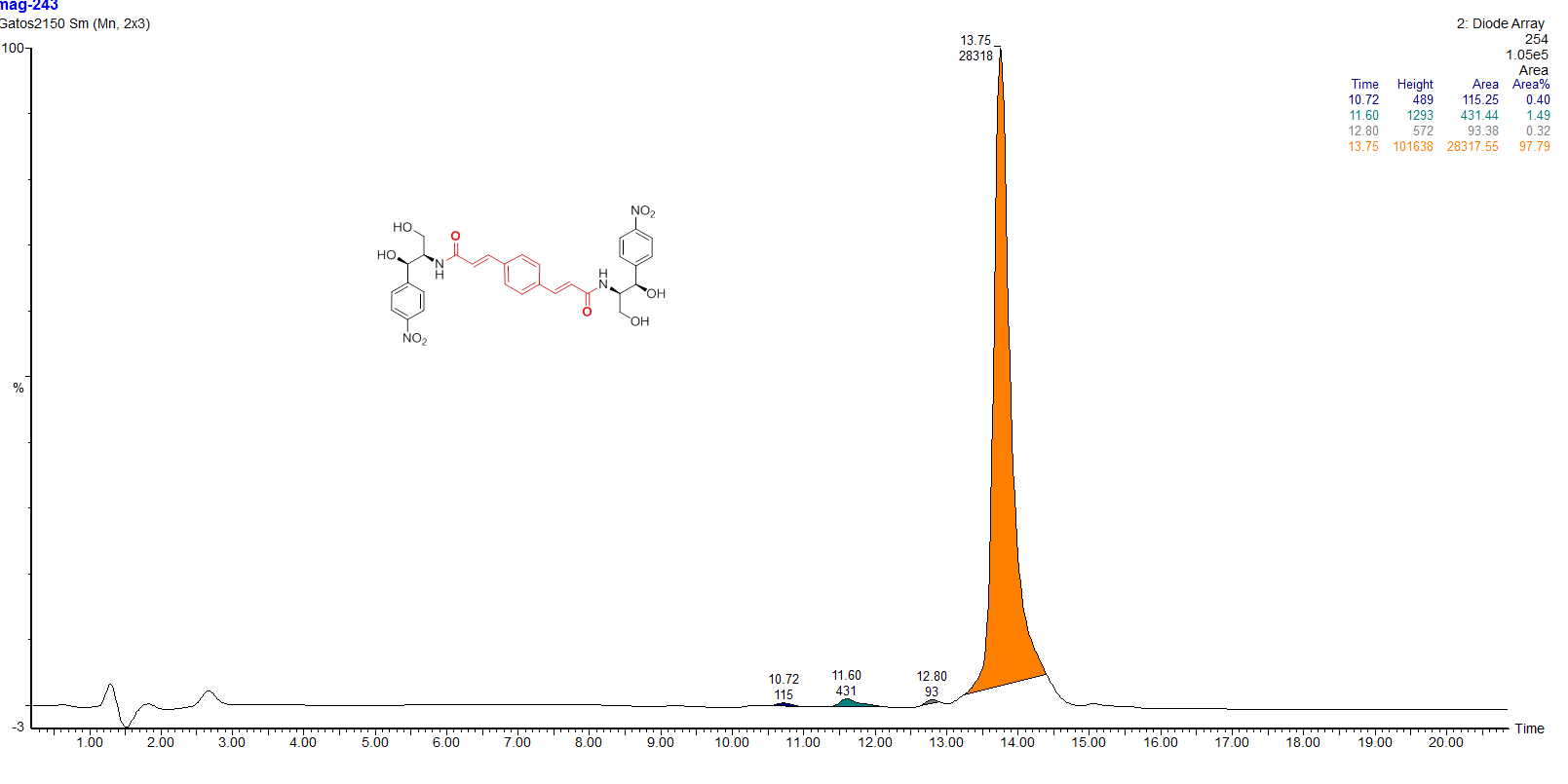
**

**6. General procedure for the synthesis of compounds 9 and 10**.

Following the procedure of Napoli et al. [1], glutaric anhydride or adipic anhydride (0.5 mmol) was added to an ice cold solution of CLB (0.106 g, 0.50 mmol) in DMF (2.5 mL), and the reaction mixture was stirred at ambient temperature for 2h. The resulting solution was diluted with EtOAc and washed twice with a 5% aqueous citric acid solution and twice with brine. The organic phase was dried over Na_2_SO_4_ and evaporated to dryness. The projected compounds were obtained pure after FCC purification using CHCl_3_/MeOH/gl. AcOH (85:10:5) as eluent.

**6.1. 5-(((1*R*,2*R*)-1,3-dihydroxy-1-(4-nitrophenyl)propan-2-yl)amino)-5-oxopentanoic acid (9).** Yield: 0.12 g (75%); White foam; R*_f_* (CHCl_3_/MeOH 7:3):0.21; IR (KBr, cm^-1^): 3400-2800, 1714, 1634, 1520, 1348, 1076, 858; MS (ESI, 30eV): *m/z* 675.23 [2M+Na], 365.35 [M+K], 349.42 [M+Na], 327.50 [M+H]; ^1^H NMR (*d_4_*-MeOH): *δ* 8.19 (d, *J* = 8.8 Hz, 2H), 7.65 (d, *J* = 8.8 Hz, 2H), 5.13 (d, *J* = 2.8 Hz, 1H), 4.23-4.17 (m, 1H), 3.78 (dd, *J* = 7.2 and 10.8 Hz, 1H), 3.58 (dd, *J* = 6.4 and 10.8 Hz, 1H), 2.17 (t, *J* = 7.6 Hz, 2H), 2.07 (t, *J* = 7.6 Hz, 2H), 1.75-1.67 (m, 2H); ^13^C NMR (*d_4_*-MeOH): *δ* 175.5, 174.0, 150.7, 147.1, 126.9 (two C), 122.7 (two C), 70.3, 61.2, 56.2, 34.5, 32.5, 20.8.

**6.2. 6-(((1*R*,2*R*)-1,3-dihydroxy-1-(4-nitrophenyl)propan-2-yl)amino)-6-oxohexanoic acid (10).** Yield: 0.124 g (73%); White foam; R*_f_* (CHCl_3_/MeOH 7:3):0.28; IR (KBr, cm^-1^): 3400-2800, 1720, 1641, 1525, 1351, 1079, 861; MS (ESI, 30eV): *m/z* 719.19 [2M+K], 703.26 [2M+Na], 379.27 [M+K], 363.33 [M+Na], 341.42 [M+H]; ^1^H NMR (*d_4_*-MeOH): *δ* 8.19 (d, *J* = 8.8 Hz, 2H), 7.65 (d, *J* = 8.8 Hz, 2H), 5.13 (d, *J* = 3.2 Hz, 1H), 4.22-4.16 (m, 1H), 3.79 (dd, *J* = 7.2 and 10.8 Hz, 1H), 3.57 (dd, *J* = 6.0 and 10.8 Hz, 1H), 2.19 (t, *J* = 6.8 Hz, 2H), 2.14 (t, *J* = 6.8 Hz, 2H), 1.50-1.42 (m, 4H); ^13^C NMR (*d_4_*-MeOH): *δ* 174.5 (two C), 150.7, 147.0, 126.9 (two C), 122.6 (two C), 70.2, 61.1, 56.1, 35.1 (two C), 25.1, 24.4.

**7. Synthesis of 4-(((1*R*,2*R*)-1,3-dihydroxy-1-(4-nitrophenyl)propan-2-yl)carbamoyl)- benzoic acid (11).**

The synthesis of this compound was realized according to Scheme S1.

**Scheme S1.** Synthesis of terephthaloyl-CAM (**11**). *Reagents and conditions*. (i) PhCOCH_2_Br, ^i^Pr_2_NEt, DMF, 0 ^o^C then RT, 12 h, 47%; (ii) CLB, Et_3_N, HBTU, DMF, 0 ^o^C then RT, 1 h, 63%; (iii) NaH, PhSH, DMF, 0 ^o^C then RT, 4 h, 55%.

**7.1.** **4-((2-oxo-2-phenylethoxy)carbonyl)benzoic acid (12)**

To an ice-cold solution of terephthalic acid (1.0 g, 6.0 mmol) and ^i^Pr_2_NEt (0.52 mL, 3.0 mmol) in DMF (18 mL), bromoacetophenone (0.6 g, 3.0 mmol) was added portion wise over 1.5 h. The resulting mixture was stirred at ambient temperature overnight. Then, a 5% aqueous citric acid solution was added and the mixture was extracted thrice with EtOAc. The combined organic phases were washed thrice with brine and evaporated to dryness. Compound **12** was obtained pure after FCC purification.

Yield: 0.4 g (47%); White solid; m.p: 232-234 ^o^C; R*_f_* (CHCl_3_/MeOH 9:1):0.31; IR (KBr, cm^-1^): 3400-2900, 1718, 1696, 1655, 1596, 1420, 1286, 1110, 956, 734; MS (ESI, 30eV): *m/z* 307.34 [M+Na], 286.51 [M+H]; ^1^H NMR (*d_6_*-DMSO): *δ* 8.14 (d, *J* = 8.8 Hz, 2H), 8.11 (d, *J* = 8.8 Hz, 2H), 8.02 (unresolved dd, 2H), 7.72 (t, *J* = 7.6 Hz, 1H), 7.59 (t, *J* = 7.6 Hz, 2H), 5.80 (s, 2H); ^13^C NMR (*d_6_*-DMSO): *δ* 192.9, 165.1, 162.6, 136.2, 134.4, 134.2, 132.8, 130.0 (two C), 129.8 (two C), 129.3 (two C), 128.2 (two C), 67.8.

**7.2.** **2-oxo-2-phenylethyl 4-(((1*R*,2*R*)-1,3-dihydroxy-1-(4-nitrophenyl)propan-2-yl) carbamoyl)benzoate (13)**

To an ice-cold solution of CLB (0.21 g, 1.0 mmol), compound **12** (0.34 g, 1.2 mmol) and Et_3_N (0.42 mL, 3.0 mmol) in DMF (1.2 mL), HBTU (0.55 g, 1.44 mmol) was added. The reaction mixture was stirred at ambient temperature for 1 h and then diluted with EtOAc. The organic phase was washed once with a 5% aqueous NaHCO_3_ solution, once with water, once with a 5% aqueous citric acid solution and once with brine and finally was dried over Na_2_SO_4_ and evaporated to dryness. Compound **13** was obtained pure after FCC purification.

Yield: 0.30 g (63%); White solid; m.p: 193-194 ^o^C; R*_f_* (CHCl_3_/MeOH 9:1):0.15; IR (KBr, cm^-1^): 3482, 3334, 2952, 1690, 1638, 1532, 1352, 1292, 1232, 1124, 864; MS (ESI, 30eV): *m/z* 517.15 [M+K], 501.24 [M+Na], 479.17 [M+H]; ^1^H NMR (*d_6_*-DMSO): *δ* 8.15 (d, *J* = 8.8 Hz, 2H), 8.09-7.99 (m, 5H), 7.90 (d, *J* = 8.4 Hz, 2H), 7.71 (t, *J* = 7.2 Hz, 1H), 7.65 (d, *J* = 8.8 Hz, 2H), 7.59 (t, *J* = 8.0 Hz, 2H), 5.79 (d, *J* = 5.6 Hz, 1H), 5.75 (s, 2H), 5.11 (unresolved dd, 1H), 4.78 (t, *J* = 5.2 Hz, 1H), 4.35-4.25 (m, 1H), 3.76-3.66 (m, 1H), 3.52-3.42 (m, 1H); ^13^C NMR (*d_6_*-DMSO): *δ* 192.9, 165.9, 165.1, 152.1, 146.7, 139.3, 134.4, 134.1, 131.5, 129.6 (two C), 129.3 (two C), 128.2 (four C), 127.8 (two C), 123.2 (two C), 70.4, 67.7, 60.7, 57.3.

**7.3. 4-(((1*R*,2*R*)-1,3-dihydroxy-1-(4-nitrophenyl)propan-2-yl)carbamoyl)benzoic acid (11)** To an ice-cold suspension of NaH (60% dispersion) (0.094 g, 2.34 mmol) in DMF (2.2 mL), thiophenol (0.24 mL, 2.34 mmol) was added and the resulting solution was stirred at ambient temperature for 15 min. Then, compound **13** (0.25 g, 0.52 mmol) was added and the reaction mixture was stirred for 4 h. After completion of the reaction, the mixture was quenched with a 5% aqueous citric acid solution and extracted thrice with EtOAc. The combined organic phases were washed twice with brine, dried over Na_2_SO_4_ and evaporated to dryness. Compound **11** was obtained pure after FCC purification.

Yield: 0.103 g (55%); White foam; R*_f_* (CHCl_3_/MeOH/gl. AcOH 9:1:0.1):0.28; IR (KBr, cm^-1^): 3400-2900, 1721, 1691, 1646, 1580, 1287, 1110, 961, 734; MS (ESI, 30eV): *m/z* 399.14 [M+K], 383.11 [M+Na], 361.19 [M+H]; ^1^H NMR (*d_4_*-MeOH): *δ* 8.18 (d, *J* = 8.8 Hz, 2H), 8.07 (d, *J* = 8.8 Hz, 2H), 7.78 (d, *J* = 8.4 Hz, 2H), 7.69 (d, *J* = 8.4 Hz, 2H), 5.23 (d, *J* = 3.2 Hz, 1H), 4.47-4.42 (m, 1H), 3.90 (dd, *J* = 6.4 and 10.8 Hz, 1H), 3.70 (dd, *J* = 6.4 and 11.2 Hz, 1H); ^13^C NMR (*d_4_*-MeOH): *δ* 168.0, 167.4, 150.4, 147.1, 138.1, 133.1, 129.2 (two C), 126.9 (two C), 126.8 (two C), 122.6 (two C), 70.6, 60.9, 57.0.

**8. ^1^H- and ^13^C-NMR spectra of compounds 9-11.**

**8.1. 5-(((1*R*,2*R*)-1,3-dihydroxy-1-(4-nitrophenyl)propan-2-yl)amino)-5-oxopentanoic acid (9).**

**
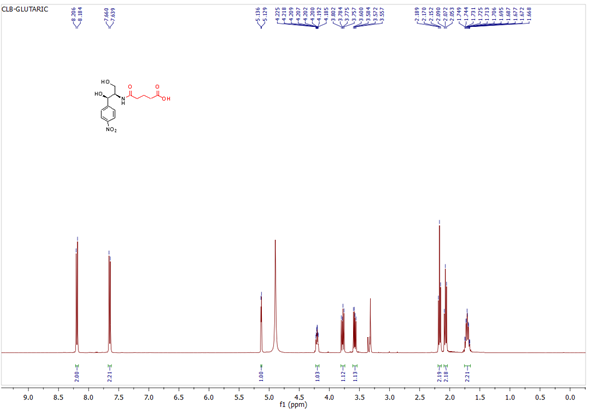
**

**
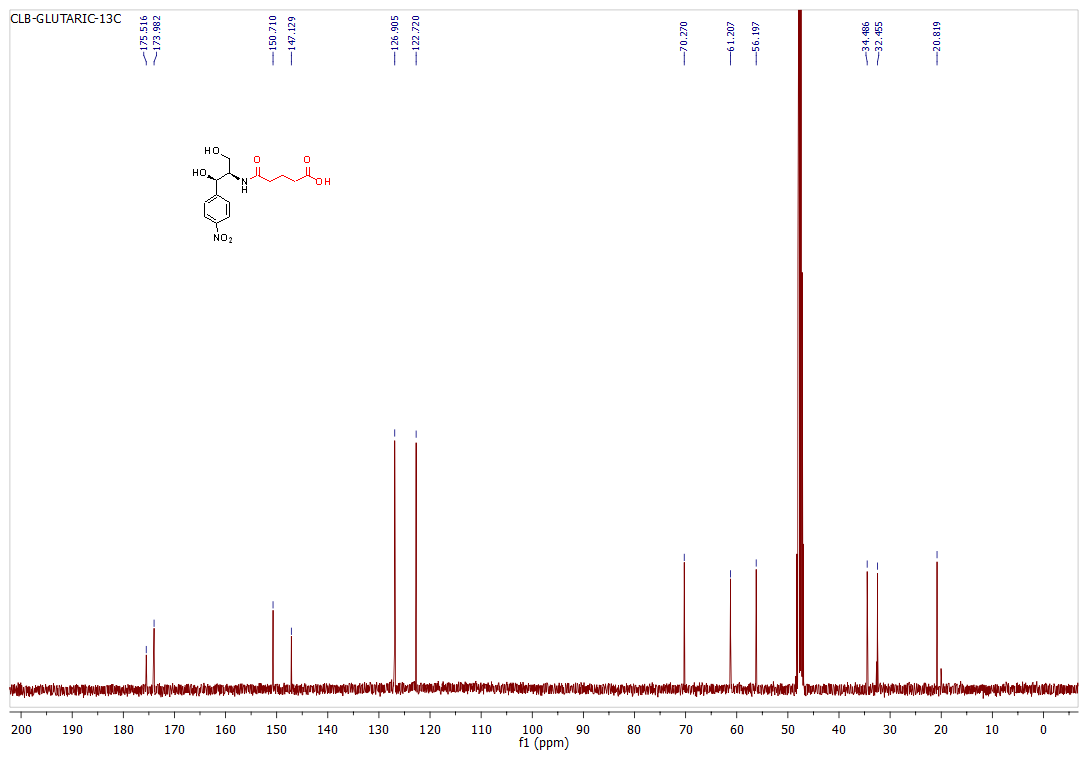
**

**8.2. 6-(((1*R*,2*R*)-1,3-dihydroxy-1-(4-nitrophenyl)propan-2-yl)amino)-6-oxohexanoic acid (10).**

**
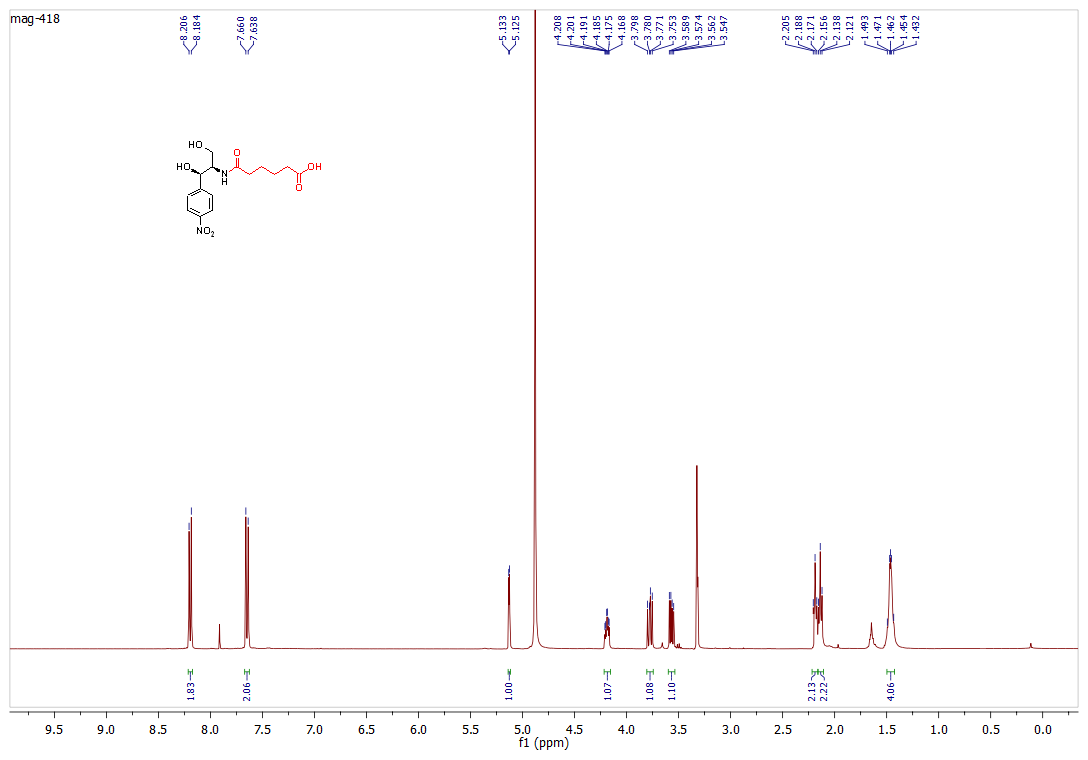

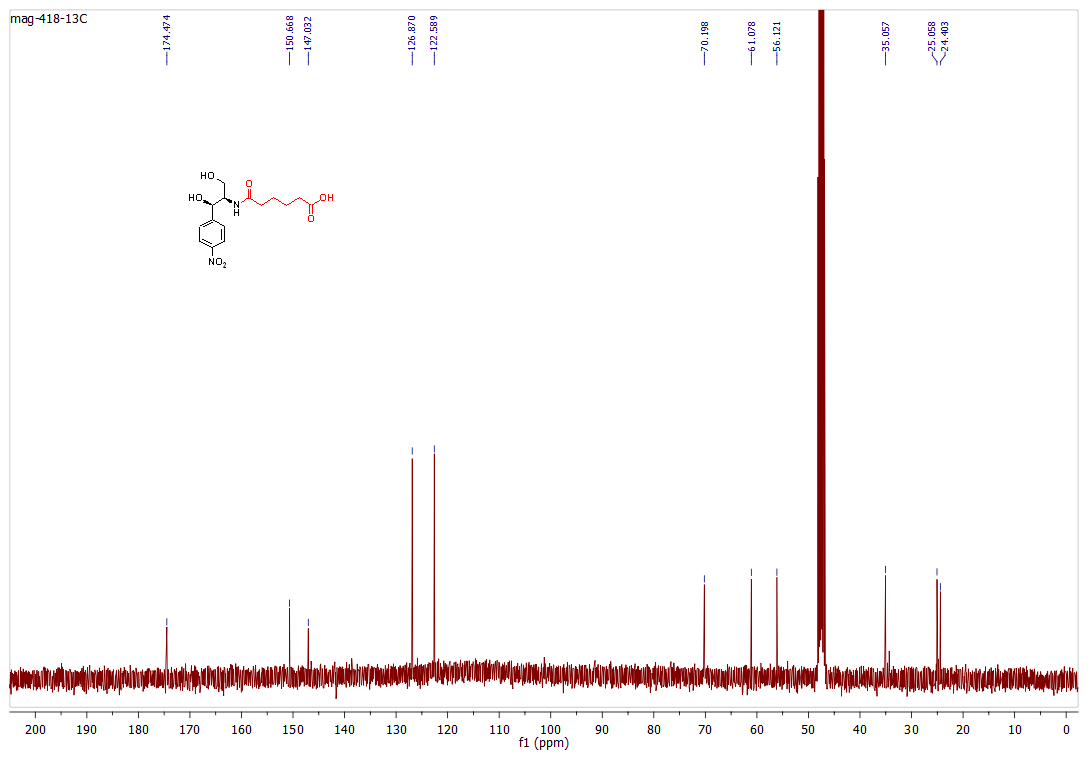
**

**8.3. 4-(((1*R*,2*R*)-1,3-dihydroxy-1-(4-nitrophenyl)propan-2-yl)carbamoyl)benzoic acid (11)**

**
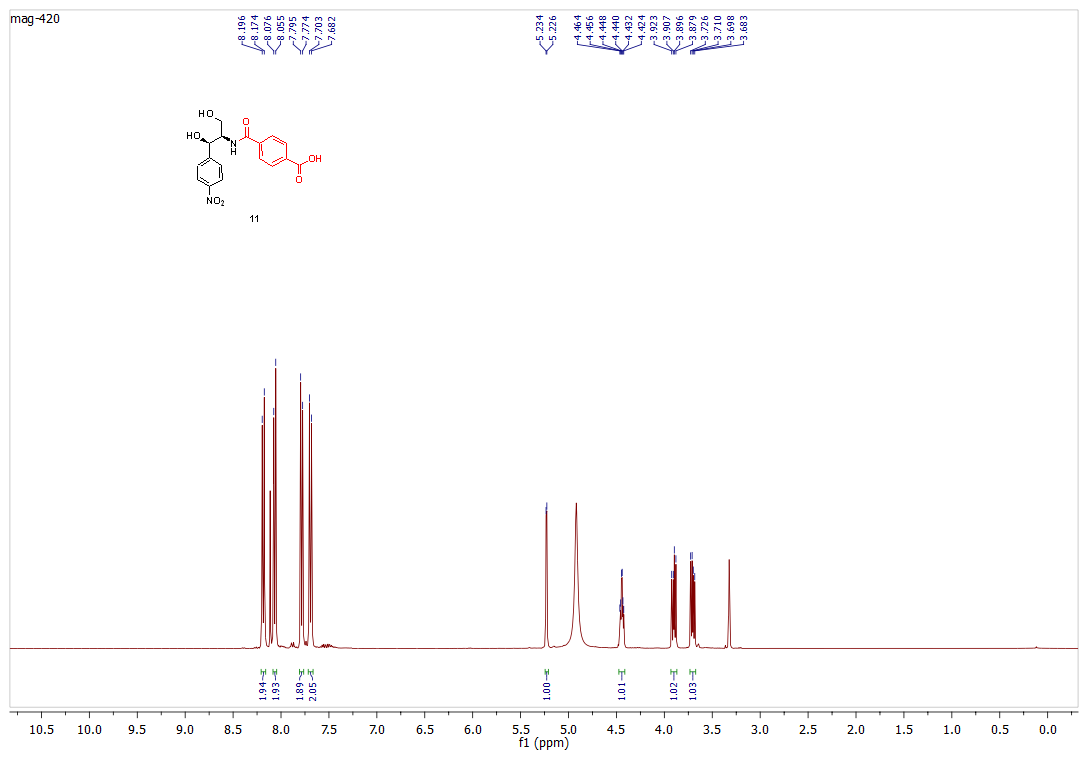
**

**
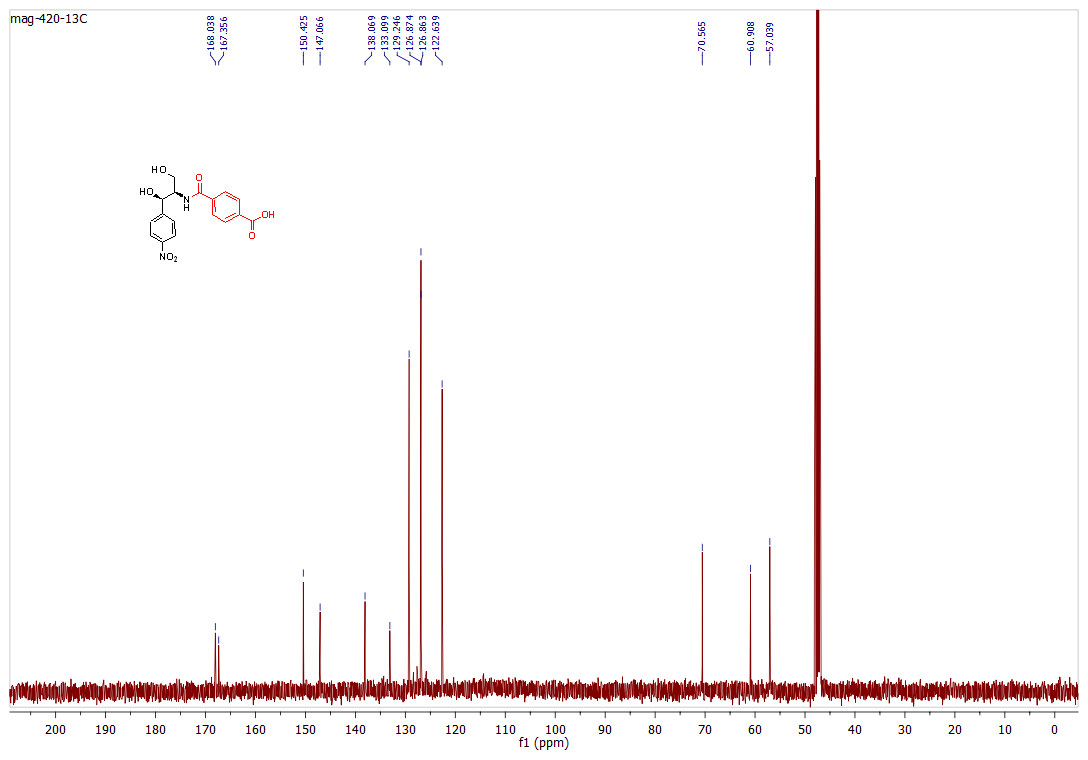
**

**Supplemental Reference**

# Napoli A, Athanassopoulos CM, Moschidis P, Aiello D, Di Donna L, Mazzotti F, Sindona G. Solid phase isobaric mass tag reagent for simultaneous protein identification and assay. Anal Chem. 2010; 82: 5552-5560.
