# Supplementary material for: Synthesis and Evaluation of Chloramphenicol Homodimers: Molecular Target, Antimicrobial Activity, and Toxicity against Human Cells
Source: PLoS One. 2015 Aug 12;10(8):e0134526. doi: 10.1371/journal.pone.0134526 (PMC4533973; doi:10.1371/journal.pone.0134526)
Supplement: S1 Table — (DOCX) [file pone.0134526.s006.docx]

**S1 Table.** Kinetic parameters of the puromycin reaction carried out in the presence of CAM attached to linkers indicated by red

| **Compounds** | **K_i_**  (μΜ) | **K_i_^*^**  (μΜ) | **k_6_/k_7_** | **k_6_**  (min^-1^) | **k_7_**  (min^-1^) |
| --- | --- | --- | --- | --- | --- |
|  | 9.50 ± 0.85 | 3.75 ± 0.30 | 1.53 ± 0.18 | 2.50 ± 0.33 | 1.63 ± 0.12 |
|  | 13.09 ± 1.05 | 4.03 ± 0.36 | 2.25 ± 0.27 | 2.52 ± 0.40 | 1.11 ± 0.12 |
|  | 5.33 ± 0.42 | 2.03 ± 0.18 | 1.63 ± 0.31 | 2.31 ± 0.48 | 1.42 ± 0.13 |
