# Supplementary material for: Synthesis and Evaluation of Chloramphenicol Homodimers: Molecular Target, Antimicrobial Activity, and Toxicity against Human Cells
Source: PLoS One. 2015 Aug 12;10(8):e0134526. doi: 10.1371/journal.pone.0134526 (PMC4533973; doi:10.1371/journal.pone.0134526)
Supplement: S2 Table — (DOCX) [file pone.0134526.s007.docx]

**S2 Table.** Bacterial strains and mechanism of resistance or hypersensitivity to antibiotics

| **Abbreviated name** | **Strain** | **Resistance or hypersensitivity phenotype** | **Mechanism of resistance or hypersensitivity to CAM** | **Source or reference** |
| --- | --- | --- | --- | --- |
| *E. faecium*  (GRE 5152) | *Enterococcus faecium* clinical isolate, *vanA* positive | Sensitive to: CAM, LZ, DAPT  Resistant to: VAN, TEIC, AMP, PEN | No resistance or hypersensitivity | Kindly offered by Prof. I. Spiliopoulou, University of Patras, Greece |
| MRSA  (GRE 2272) | Methicillin-resistant, Community-associated *Staphylococcus aureus* isolate belonging to clone ST80 and carrying the type IV staphylococcal chromosome cassette (SCC*mec*) | Sensitive to: VAN, TEIC, DAPT, QUIP/DALFP, RIF, SXT, CAM  Resistant to: MET, OXA, CEF, KAN, GM, TET, ERY, CLINDA, FA | No resistance or hypersensitivity | National Reference Laboratory for Staphylococci, School of Medicine, University of Patras  (Prof. I. Spiliopoulou) |
| MRSA  (GRE 2691) | Methicillin-resistant, Hospital-associated *Staphylococcus aureus* isolate belonging to clone ST80 and carrying the type IV staphylococcal chromosome cassette (SCC*mec*) | Sensitive to: DAPT, RIF, SXT, QUIP/DALF, CAM CLINDA  Resistant to: MET, OXA, CEF, KAN, GM, TET, ERY, FA | No resistance or hypersensitivity | National Reference Laboratory for Staphylococci, School of Medicine, University of Patras  (Prof. I. Spiliopoulou) |
| *S. aureus*  (WT) | *Staphylococcus aureus,* strain 3399, wild type | Sensitive to CAM | No resistance or hypersensitivity | Kindly offered by Prof. I. Spiliopoulou, University of Patras, Greece  [1] |
| *E. coli*  (WT) | *Escherichia coli,* strain K12,  wild-type | Sensitive to CAM | No resistance or hypersensitivity | Sigma-Aldrich |
| *E. coli*  (A2058G) | *Escherichia coli*, strain TA531 lacking *rrn* alleles and carrying plasmid pKK3535 that contains a complete copy of the rRNA operon mutated at A2058 of 23S rRNA | Reduced susceptible to CAM | Target mutation | Kindly offered by Prof. A.S. Mankin, University of Illinois  [2,3,4,5] |
| *E. coli*  (A2503C) | *Escherichia coli*, strain TA531 lacking *rrn* alleles and carrying plasmid pKK3535 that contains a complete copy of the rRNA operon mutated at A2503 of 23S rRNA | Resistant to CAM | Target mutation | Kindly offered by Prof. A.S. Mankin, University of Illinois  [2,3,6] |
| *E. coli*  (Δ*ΤolC*) | *Escherichia coli*, strain BL21 DE3- Δ*ΤolC* | Hypersensitive to CAM | Impaired AcrAB-TolC MDR efflux pump | Kindly offered by Dr D.N. Wilson, University of Munich |
| *E. coli*  Rosetta(DE3)pLysS | *Escherichia coli*, BL21 DE3 derivative carrying plasmid pLysS that contains the *cat* gene  Genotype: F^-^ *ompT hsdS*_B_(r_B_^-^ m_B_^-^) *gal dcm* (DE3) pLysSRARE (Cam^R^) | Resistant to CAM | Expression of CAM acetyltransferase gene (*cat*) that inactivates CAM by acetylation | Novagen  [7] |
| *P. aeruginosa*  (GRE 5288) | *Pseudomonas aeruginosa*  clinical isolate | Sensitive to: AMI, NET, TOB, CIPRO, GM, COL  Resistant to: PIPE, CEF, IMI,  AZR, TIR/CLAV, CAM | Intrinsic low permeability of the outer membrane,  intrinsic or induced by CAM efflux pump(s) | Kindly offered by Prof. I. Spiliopoulou, University of Patras, Greece  [8,9] |

Abbreviations: MET, methicillin; OXA, oxacillin; VAN, vancomycin; TEIC, teicoplanin; DAPT, daptomycin; QUIP/DALFP, quinapristin/ dalfopristin; CEF, cefoxitin; KAN, kanamycin; GM, gentamicin; TET, tetracycline; RIF, rifampisin; ERY, erythromycin; CLINDA, clindamycin; FA, fusidic acid; SXT, trimethoprim/sulfamethoxazole; LZ, linezolid; CAM, chloramphenicol; AMP, ampicillin, PEN, penicillin; AMI, amikasin; NET, netilmicin; TOB, tobramycin; CIPRO, ciprofloxacin; COL, colistin; PIPE, piperacillin; CEF, cefepim; CEFT, ceftazidime; IMI, imipenem; AZR, aztreonam; TIR/CLAV, ticarcillin/clavulanic acid.

**References to Table B**

1. Drougka E, Foka A, Liakopoulos A, Doudoulakakis A, Jelastopulu E, Chini V, Spiliopoulou A, Levidiotou S, Panagea T, Vogiatzi A, Lebessi E, Petinaki E, Spiliopoulou I. A 12-year survey of methicillin-resistant *Staphylococcus aureus* infections in Greece: ST80-IV epidemic? Clin Microbiol Infect. 2014; 20: 796-803.
2. Xiong L, Korkhin Y, Mankin AS. Binding site of the bridged macrolides in the *Escherichia coli* ribosome. Antimicrob Agents Chemother. 2005; 49: 281-288.
3. Asai T, Zaporojets D, Squires C, Squires CL. An *Escherichia coli* strain with all chromosomal rRNA operons inactivated: Complete exchange of rRNA genes between bacteria. Proc Natl Acad Sci USA 1999; 96: 1971-1976.
4. Brosius J, Ullrich A, Raker MA, Gray A, Dull TJ, Gutell RR, Noller HF. Construction and fine mapping of recombinant plasmids containing the *rrn*B ribosomal operon of *E. coli.* Plasmids 1981; 6: 112-118.
5. Douthwaite S. Functional interactions within 23S rRNA involving the peptidyltransferase center. J Bacteriol. 1992; 174: 1333-1338.
6. Vester B, Garrett RA. The importance of highly conserved nucleotides in the binding region of chloramphenicol at the peptidyl transferase centre of *Escherichia coli* 23S ribosomal RNA. EMBO J. 1988; 7: 3577-3587.
7. Shaw WV. Chloramphenicol acetyltransferase: enzymology and molecular biology. CRC Crit Rev Biochem. 1983; 14: 1-46.
8. Li X-Z, Livermore DM, Nikaido H. Role of efflux pump(s) in intrinsic resistance of *Pseudomonas aeruginosa*: Resistance to tetracycline, chloramphenicol and norfloxacin. Antimicrob Agents Chemother. 1994; 38: 1732-1741.
9. Morita Y, Tomida J, Kawamura Y. Responses of *Pseudomonas aeruginosa* to antimicrobials. Front Microbiol. 2014; 4: 422.
