# Supplementary material for: Synthesis and Evaluation of Chloramphenicol Homodimers: Molecular Target, Antimicrobial Activity, and Toxicity against Human Cells
Source: PLoS One. 2015 Aug 12;10(8):e0134526. doi: 10.1371/journal.pone.0134526 (PMC4533973; doi:10.1371/journal.pone.0134526)
Supplement: S3 Table — (DOCX) [file pone.0134526.s008.docx]

**S3 Table.** Determination of the ratio EC_50(cell growth)_/IC_50(puro)_ in wild-type *E. coli* for CAM and CAM dimers *^a,b^*

| **Compound** | **EC_50(cell growth)_/IC_50(puro)_** |
| --- | --- |
| **CAM** | 0.84 ± 0.07 |
| **1** | >55.5 |
| **2** | >44.4 |
| **3** | 7.28 ± 1.08 |
| **4** | 7.45 ± 0.81 |
| **5** | 14.06 ± 1.75 |
|  |  |
|  |  |
| **6** | >14.5 |
| **7** | >11.9 |

*^a^*Data represent the mean±SE values obtained from three independently performed experiments, with two replicates per experiment.

*^b^*IC_50(cell growth)_ values were taken from Table 3. The term IC_50(puro)_ is defined as the compound concentration causing 50% inhibition in peptide-bond formation at the presence of 2 mM puromycin, and its value was calculated through the relationship,

$${IC}_{50\left( puro \right)}=K_{i}^{*}(1+ \frac{\left[ S \right]}{K_{S}} )$$
